# Supplementary material for: Synthesis and Evaluation on the Fungicidal Activity of S-Alkyl Substituted Thioglycolurils
Source: Int J Mol Sci. 2023 Mar 17;24(6):5756. doi: 10.3390/ijms24065756 (PMC10051383; doi:10.3390/ijms24065756)

Supplementary Information

Table of Contents

1. Spectra ^{1}H and ^{13}C NMR of compounds **4a-f** S2
2. Spectra ^{1}H and ^{13}C NMR of compounds **5a-f** S14
3. Spectra ^{1}H and ^{13}C NMR of compounds **6a-h** S26
4. LC-MS data of compound **4f** S42
5. Anticancer Screening Data of 60 Cancer Cell Lines of **4b**, **5d**, **5e**, **5f** S48

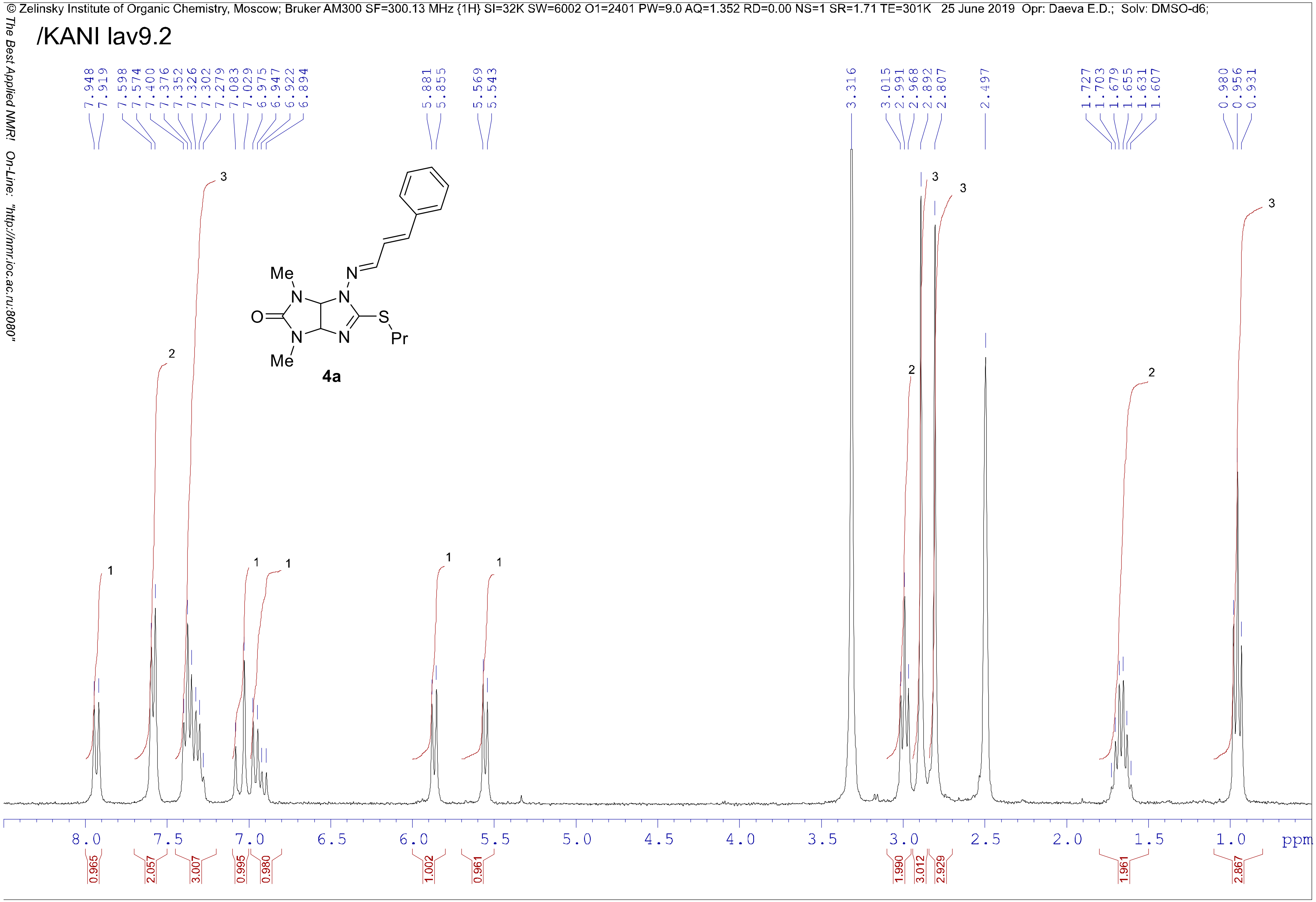
© Zelinsky Institute of Organic Chemistry, Moscow; Bruker AM300 SF=300.13 MHz {1H} SI=32K SW=6002 O1=2401 PW=9.0 AQ=1.352 RD=0.00 NS=1 SR=1.71 TE=301K 25 June 2019 Opr: Daeva E.D.; Solv: DMSO-d6;
The Best Applied NMR! On-Line: "http://nmr.ioc.ac.ru:8080"
/KANI lav9.2
7.948 7.919 7.598 7.574 7.400 7.376 7.352 7.326 7.302 7.279 7.083 7.029 6.975 6.947 6.922 6.894
5.881 5.855 5.569 5.543
3.316 3.015 2.991 2.968 2.892 2.807 2.497
1.727 1.703 1.679 1.655 1.631 1.607 0.980 0.956 0.931
Me
N
O
N
Me
N
N
S
Pr
4a
8.0 7.5 7.0 6.5 6.0 5.5 5.0 4.5 4.0 3.5 3.0 2.5 2.0 1.5 1.0 ppm
0.965 2.057 3.007 0.995 0.980 1.002 0.961 1.990 3.012 2.929 1.961 2.867

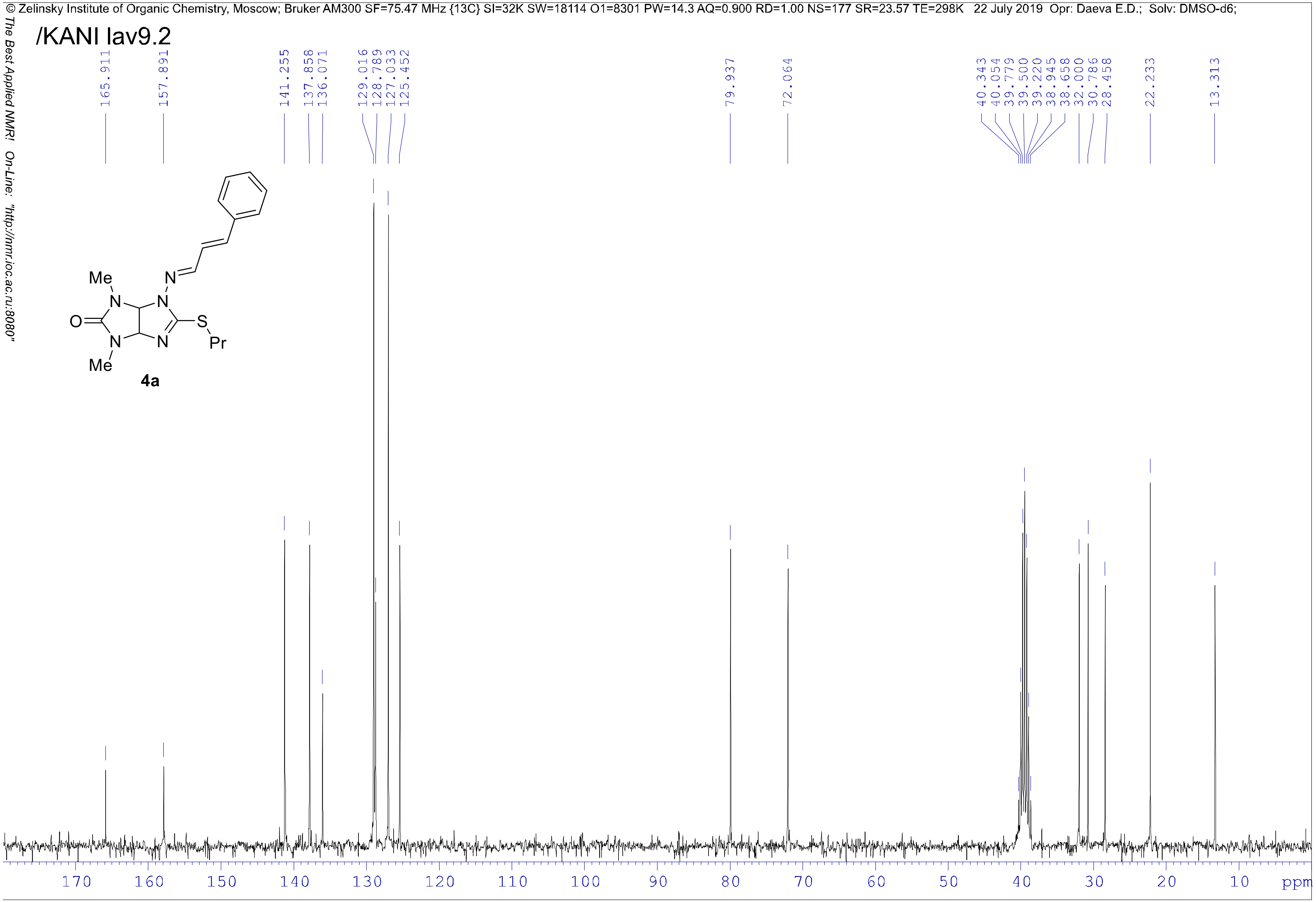
© Zelinsky Institute of Organic Chemistry, Moscow; Bruker AM300 SF=75.47 MHz {13C} SI=32K SW=18114 O1=8301 PW=14.3 AQ=0.900 RD=1.00 NS=177 SR=23.57 TE=298K 22 July 2019 Opr: Daeva E.D.; Solv: DMSO-d6;
The Best Applied NMR! On-Line: "http://nmr.ioc.ac.ru:8080"
/KANI lav9.2
165.911
157.891
141.255
137.858
136.071
129.016
128.789
127.033
125.452
79.937
72.064
40.343
40.054
39.779
39.500
39.220
38.945
38.658
32.000
30.786
28.458
22.233
13.313
170
160
150
140
130
120
110
100
90
80
70
60
50
40
30
20
10
ppm
Me
N
O
N
Me
N
N
N
S
Pr
4a

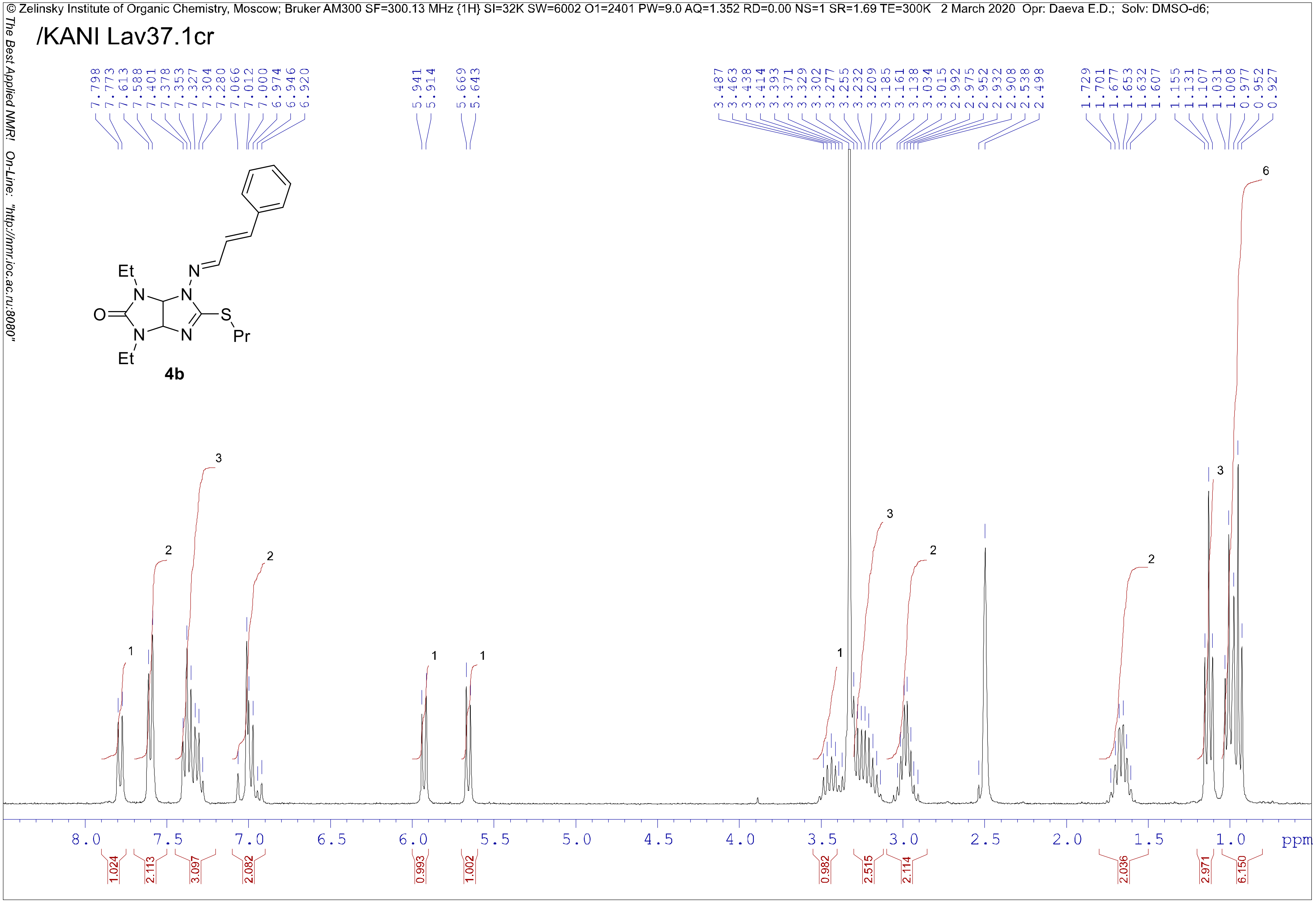
© Zelinsky Institute of Organic Chemistry, Moscow; Bruker AM300 SF=300.13 MHz {1H} SI=32K SW=6002 O1=2401 PW=9.0 AQ=1.352 RD=0.00 NS=1 SR=1.69 TE=300K 2 March 2020 Opr: Daeva E.D.; Solv: DMSO-d6;
/KANI Lav37.1cr
The Best Applied NMR! On-Line: "http://nmr.ioc.ac.ru:8080"
7.798 7.773 7.613 7.588 7.401 7.378 7.353 7.327 7.304 7.280 7.066 7.012 7.000 6.974 6.946 6.920
5.941 5.914 5.669 5.643
3.487 3.463 3.438 3.414 3.393 3.371 3.329 3.302 3.277 3.255 3.232 3.209 3.185 3.161 3.138 3.034 3.015 2.992 2.975 2.952 2.932 2.908 2.538 2.498
1.729 1.701 1.677 1.653 1.632 1.607 1.155 1.131 1.107 1.031 1.008 0.977 0.952 0.927
4b
ppm
1.024 2.113 3.097 2.082 0.993 1.002 0.982 2.515 2.114 2.036 2.971 6.150

© Zelinsky Institute of Organic Chemistry, Moscow; Bruker AM300 SF=75.47 MHz {13C} SI=32K SW=18114 O1=8301 PW=14.3 AQ=0.900 RD=1.00 NS=181 SR=36.70 TE=300K 3 March 2020 Opr: Daeva E.D.; Solv: DMSO-d6;

The Best Applied NMR! On-Line: "http://nmr.ioc.ac.ru:8080"

/KANI Lav37.1cr

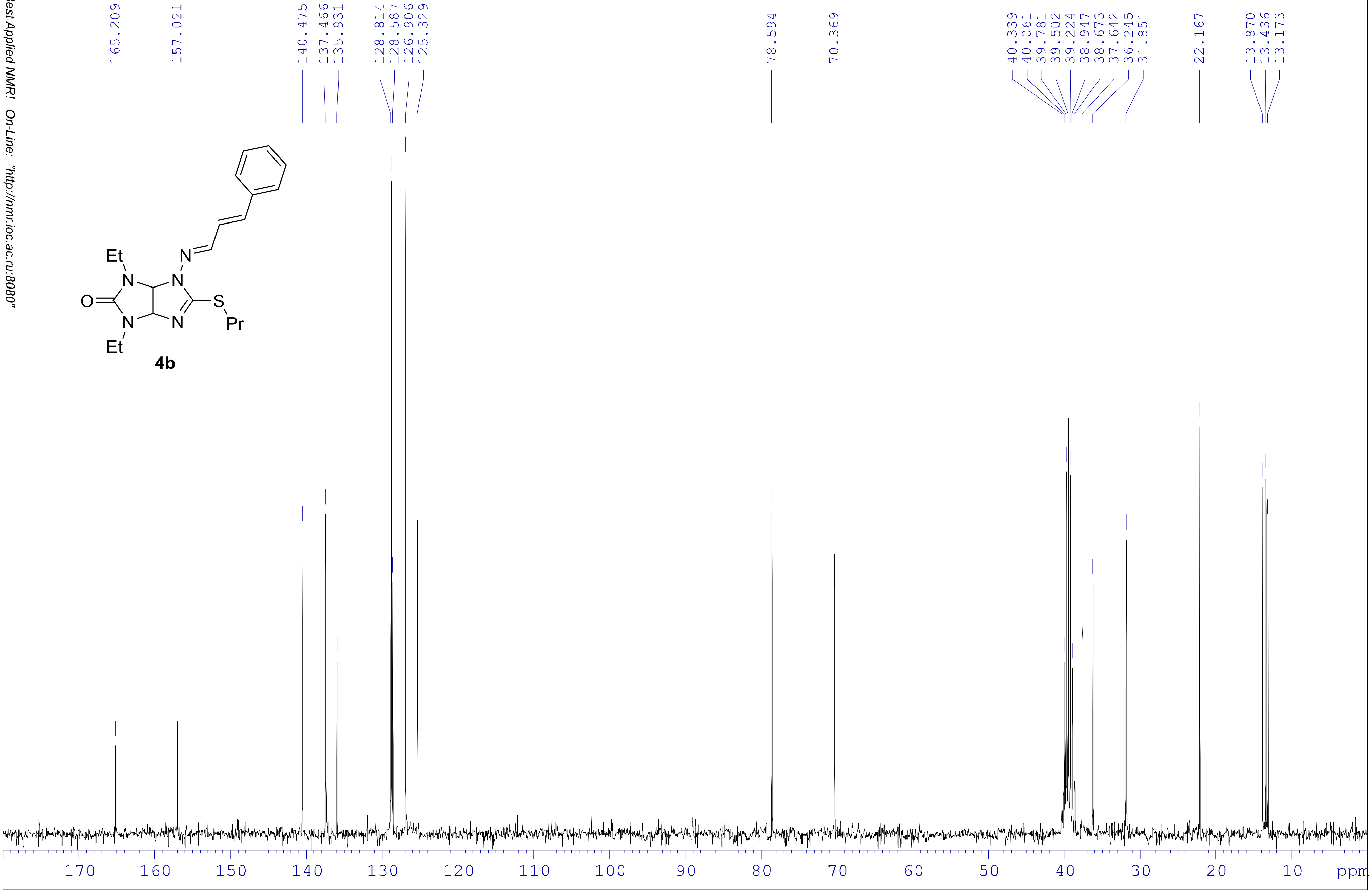

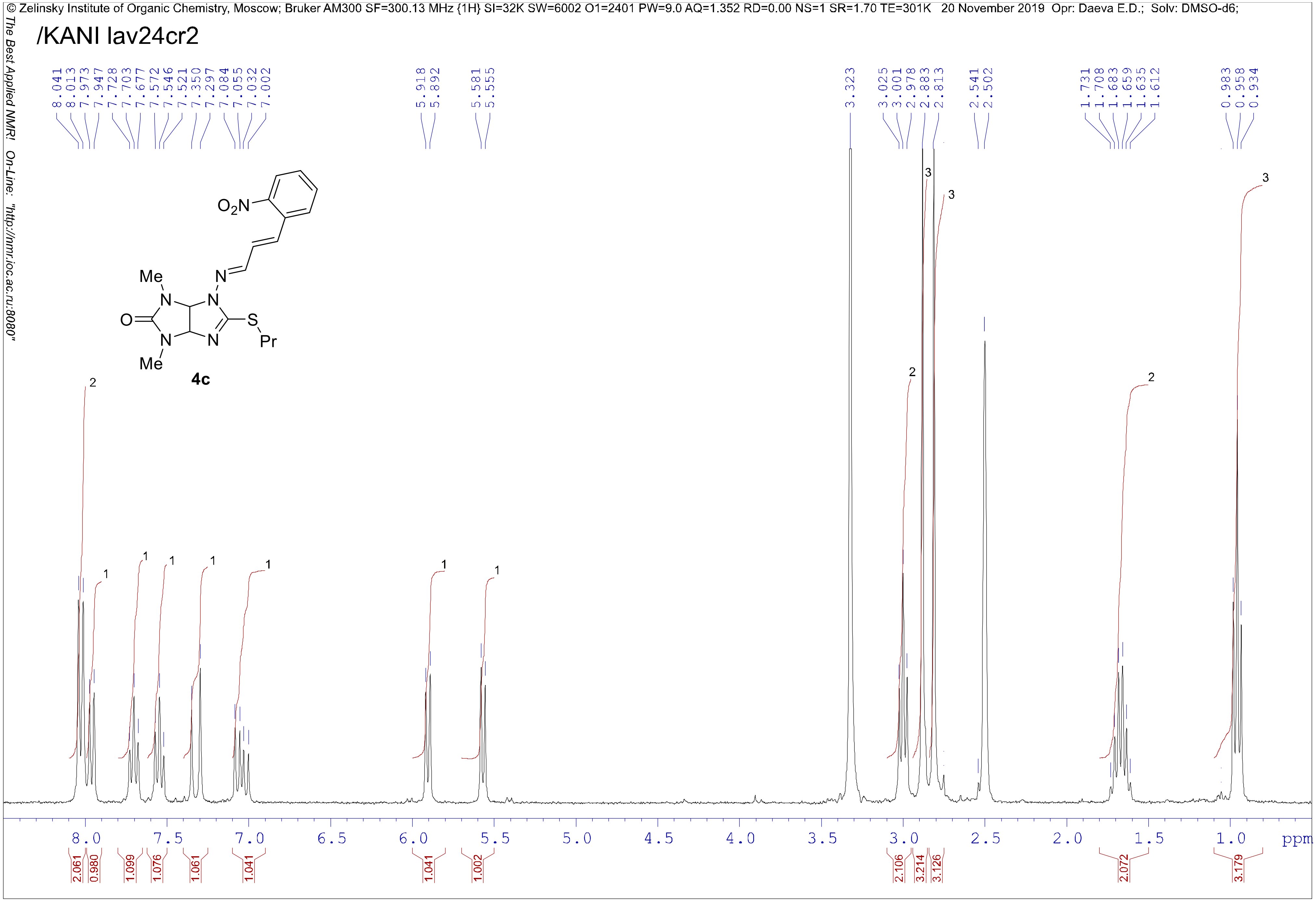
© Zelinsky Institute of Organic Chemistry, Moscow; Bruker AM300 SF=300.13 MHz {1H} SI=32K SW=6002 O1=2401 PW=9.0 AQ=1.352 RD=0.00 NS=1 SR=1.70 TE=301K 20 November 2019 Opr: Daeva E.D.; Solv: DMSO-d6;
The Best Applied NMR! On-Line: "http://nmr.ioc.ac.ru:8080"
/KANI lav24cr2
8.041 8.013 7.973 7.947 7.728 7.703 7.677 7.572 7.546 7.521 7.350 7.297 7.084 7.055 7.032 7.002
5.918 5.892
5.581 5.555
3.323
3.025 3.001 2.978 2.883 2.813
2.541 2.502
1.731 1.708 1.683 1.659 1.635 1.612
0.983 0.958 0.934
O2N
N
Me
N
N
O
N
N
S
Pr
Me
4c
ppm
8.0 7.5 7.0 6.5 6.0 5.5 5.0 4.5 4.0 3.5 3.0 2.5 2.0 1.5 1.0
2.061 0.980 1.099 1.076 1.061 1.041 1.041 1.002 2.106 3.214 3.126 2.072 3.179

© Zelinsky Institute of Organic Chemistry, Moscow; Bruker AM300 SF=75.47 MHz {13C} SI=32K SW=18114 O1=8301 PW=14.3 AQ=0.900 RD=1.00 NS=224 SR=38.41 TE=302K 21 October 2019 Opr: Daeva E.D.; Solv: DMSO-d6;

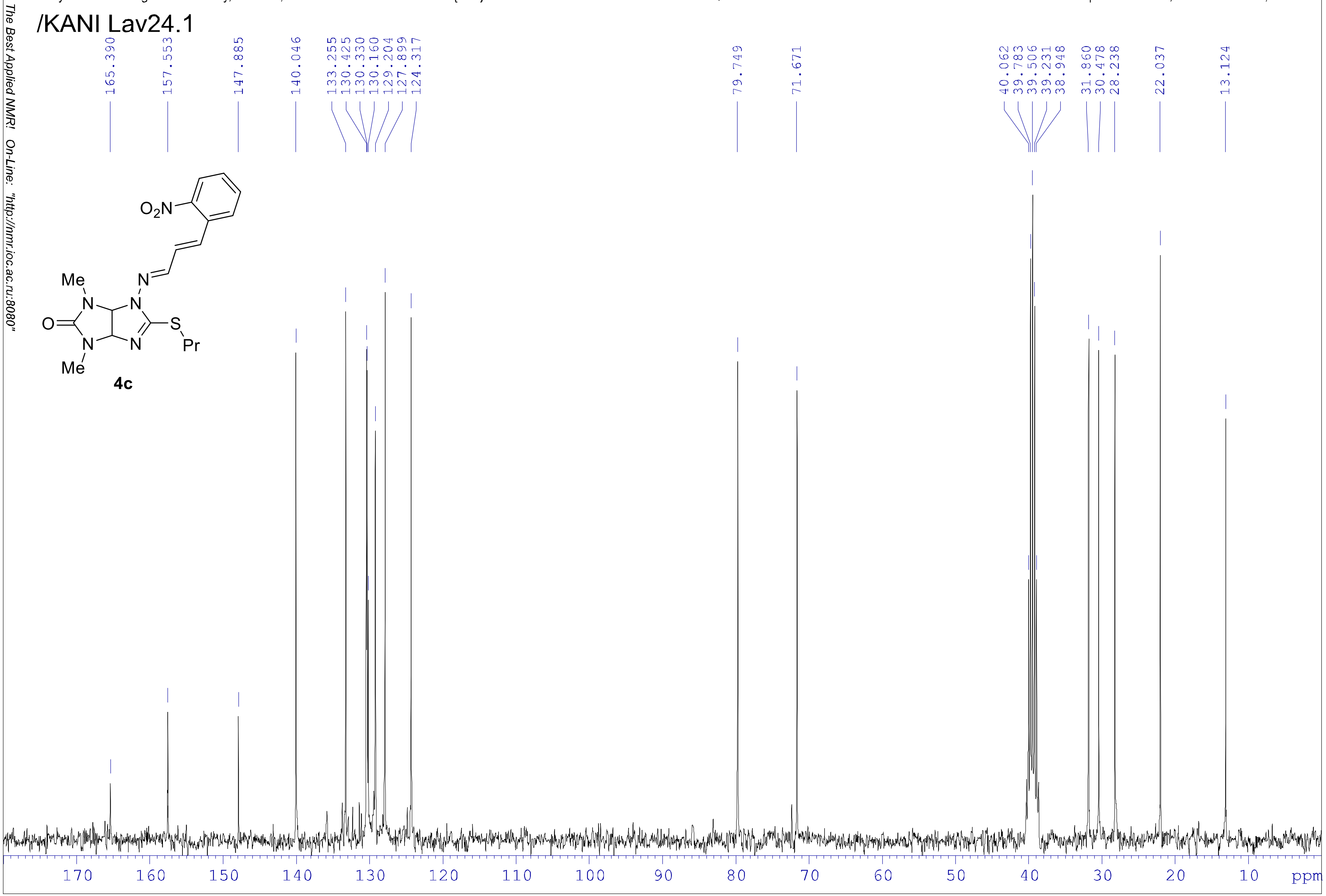

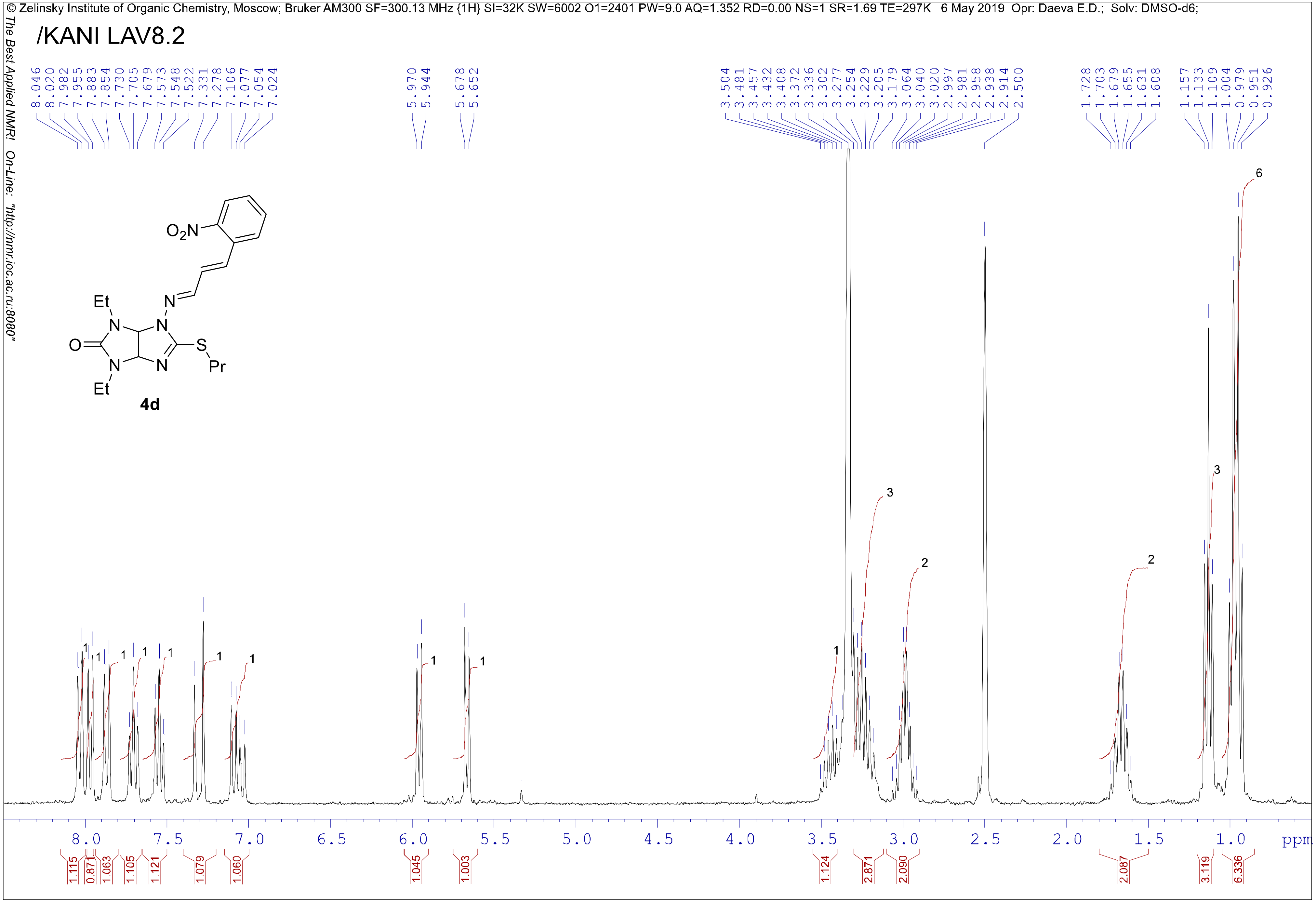
© Zelinsky Institute of Organic Chemistry, Moscow; Bruker AM300 SF=300.13 MHz {1H} SI=32K SW=6002 O1=2401 PW=9.0 AQ=1.352 RD=0.00 NS=1 SR=1.69 TE=297K 6 May 2019 Opr: Daeva E.D.; Solv: DMSO-d6;
/KANI LAV8.2
The Best Applied NMR! On-Line: "http://nmr.ioc.ac.ru:8080"
8.046 8.020 7.982 7.955 7.883 7.854 7.730 7.705 7.679 7.573 7.548 7.522 7.331 7.278 7.106 7.077 7.054 7.024
5.970 5.944 5.678 5.652
3.504 3.481 3.457 3.432 3.408 3.372 3.336 3.302 3.277 3.254 3.229 3.205 3.179 3.064 3.040 3.020 2.997 2.981 2.958 2.938 2.914 2.500
1.728 1.703 1.679 1.655 1.631 1.608 1.157 1.133 1.109 1.004 0.979 0.951 0.926
O2N
Et
N
O
S
Pr
4d
ppm
8.0 7.5 7.0 6.5 6.0 5.5 5.0 4.5 4.0 3.5 3.0 2.5 2.0 1.5 1.0
1.115 0.871 1.063 1.105 1.121 1.079 1.060 1.045 1.003 1.124 2.871 2.090 2.087 3.119 6.336

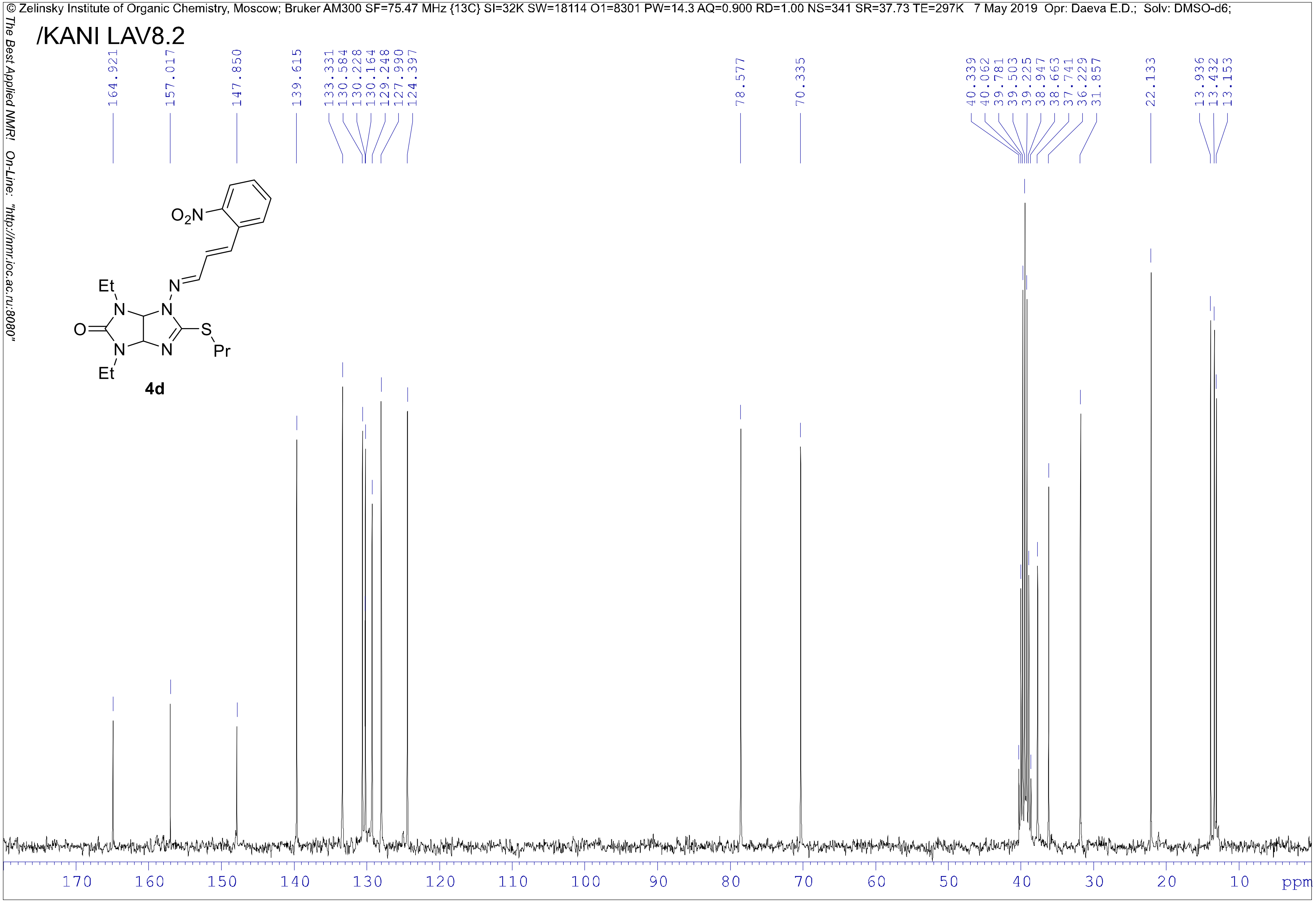
© Zelinsky Institute of Organic Chemistry, Moscow; Bruker AM300 SF=75.47 MHz {13C} SI=32K SW=18114 O1=8301 PW=14.3 AQ=0.900 RD=1.00 NS=341 SR=37.73 TE=297K 7 May 2019 Opr: Daeva E.D.; Solv: DMSO-d6;
The Best Applied NMR! On-Line: "http://nmr.ioc.ac.ru:8080"
/KANI LAV8.2
164.921
157.017
147.850
139.615
133.331
130.584
130.228
130.164
129.248
127.990
124.397
78.577
70.335
40.339
40.062
39.781
39.503
39.225
38.947
38.663
37.741
36.229
31.857
22.133
13.936
13.432
13.153
O_2N
N
Et
N
N
O
S
N
Pr
N
Et
4d
170
160
150
140
130
120
110
100
90
80
70
60
50
40
30
20
10
ppm

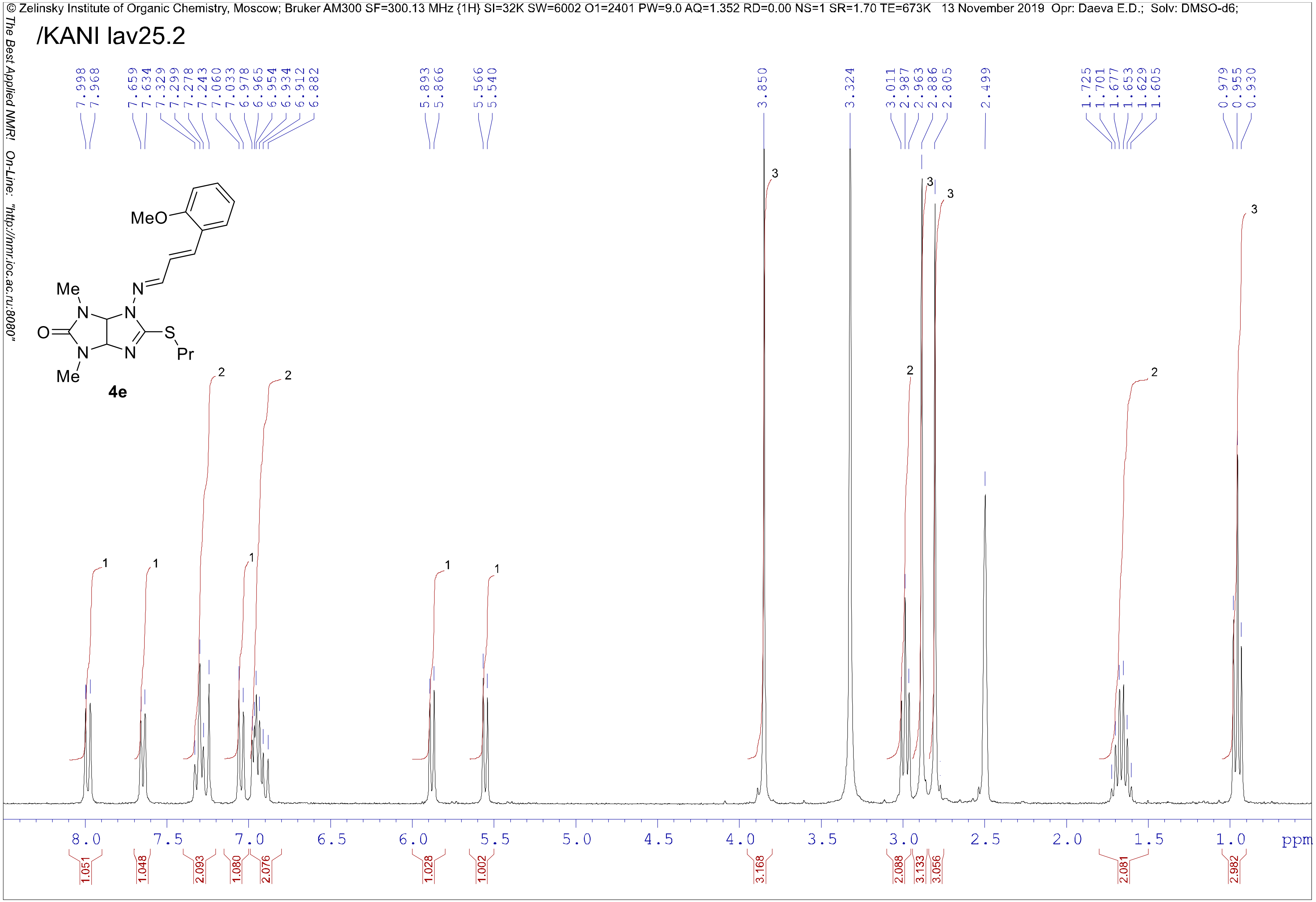

© Zelinsky Institute of Organic Chemistry, Moscow; Bruker AM300 SF=300.13 MHz {1H} SI=32K SW=6002 O1=2401 PW=9.0 AQ=1.352 RD=0.00 NS=1 SR=1.70 TE=673K 13 November 2019 Opr: Daeva E.D.; Solv: DMSO-d6;
/KANI lav25.2
The Best Applied NMR! On-Line: "http://nmr.ioc.ac.ru:8080"
7.998 7.968 7.659 7.634 7.329 7.299 7.278 7.243 7.060 7.033 6.978 6.965 6.954 6.934 6.912 6.882
5.893 5.866 5.566 5.540
3.850 3.324 3.011 2.987 2.963 2.886 2.805 2.499
1.725 1.701 1.677 1.653 1.629 1.605 0.979 0.955 0.930
MeO
Me
N
O
Me
S
Pr
4e
ppm
1.051 1.048 2.093 1.080 2.076 1.028 1.002 3.168 2.088 3.133 3.056 2.081 2.982

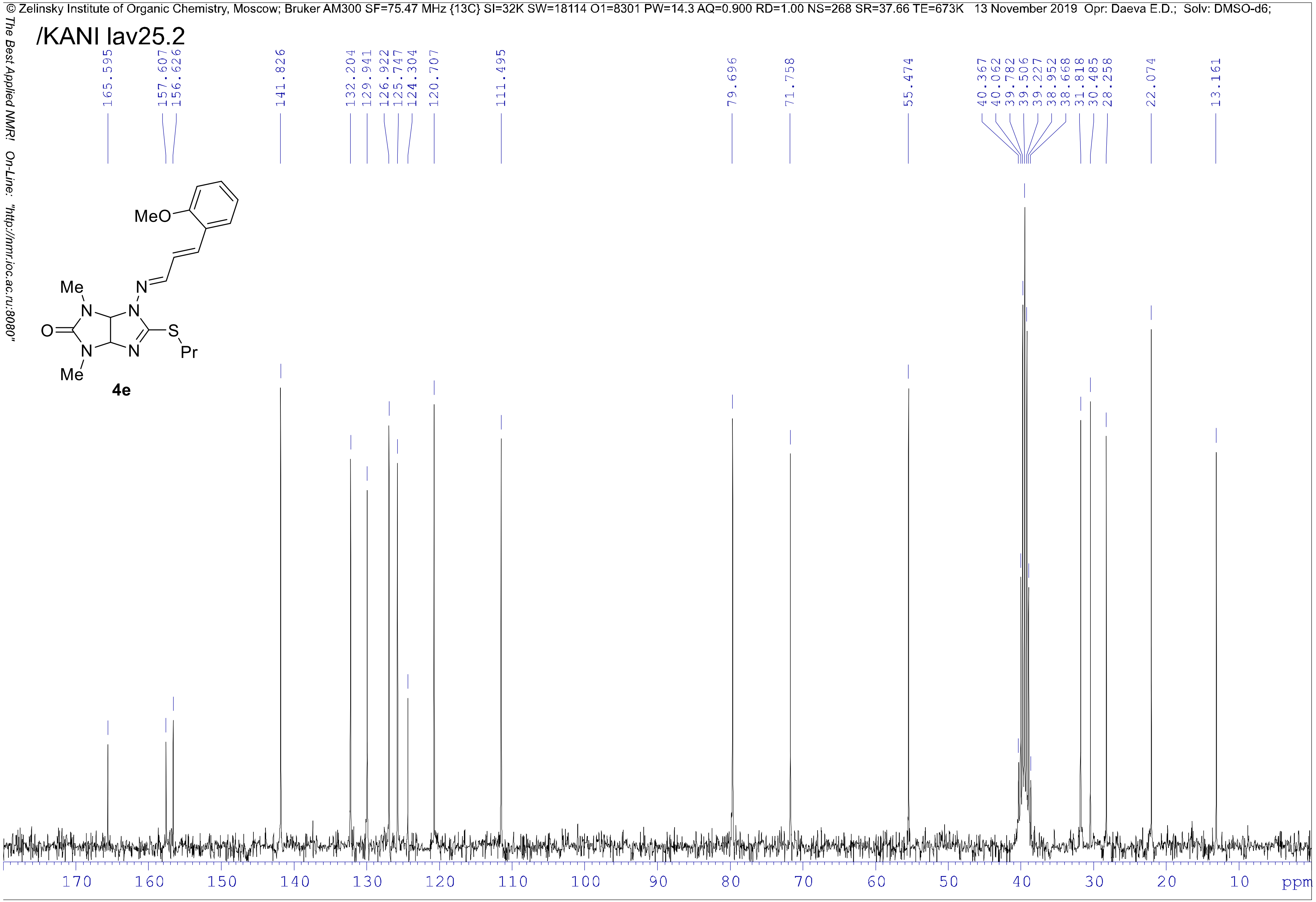
© Zelinsky Institute of Organic Chemistry, Moscow; Bruker AM300 SF=75.47 MHz {13C} SI=32K SW=18114 O1=8301 PW=14.3 AQ=0.900 RD=1.00 NS=268 SR=37.66 TE=673K 13 November 2019 Opr: Daeva E.D.; Solv: DMSO-d6;
The Best Applied NMR! On-Line: "http://nmr.ioc.ac.ru:8080"
/KANI lav25.2
165.595
157.607
156.626
141.826
132.204
129.941
126.922
125.747
124.304
120.707
111.495
79.696
71.758
55.474
40.367
40.062
39.782
39.506
39.227
38.952
38.668
31.818
30.485
28.258
22.074
13.161
MeO
Me
N
N
N
O
S
Pr
Me
4e
170
160
150
140
130
120
110
100
90
80
70
60
50
40
30
20
10
ppm

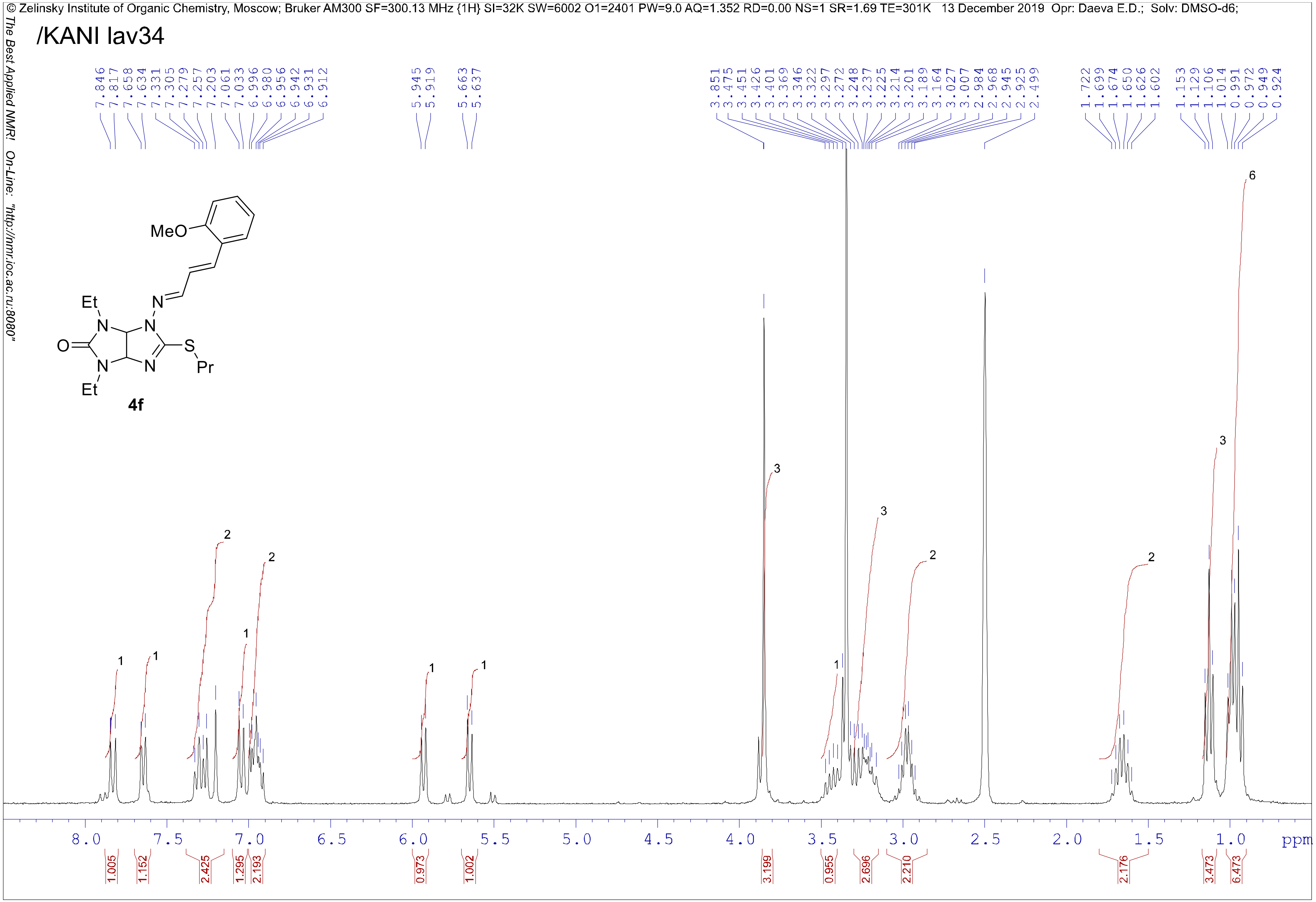
© Zelinsky Institute of Organic Chemistry, Moscow; Bruker AM300 SF=300.13 MHz {1H} SI=32K SW=6002 O1=2401 PW=9.0 AQ=1.352 RD=0.00 NS=1 SR=1.69 TE=301K 13 December 2019 Opr: Daeva E.D.; Solv: DMSO-d6;
The Best Applied NMR! On-Line: "http://nmr.ioc.ac.ru:8080"
/KANI lav34
MeO
Et
N
O
S
Pr
4f
ppm

© Zelinsky Institute of Organic Chemistry, Moscow; Bruker AM300 SF=75.47 MHz {13C} SI=32K SW=18114 O1=8301 PW=14.3 AQ=0.900 RD=1.00 NS=405 SR=33.77 TE=302K 23 December 2019 Opr: Daeva E.D.; Solv: DMSO-d6;

The Best Applied NMR! On-Line: "http://nmr.ioc.ac.ru:8080"

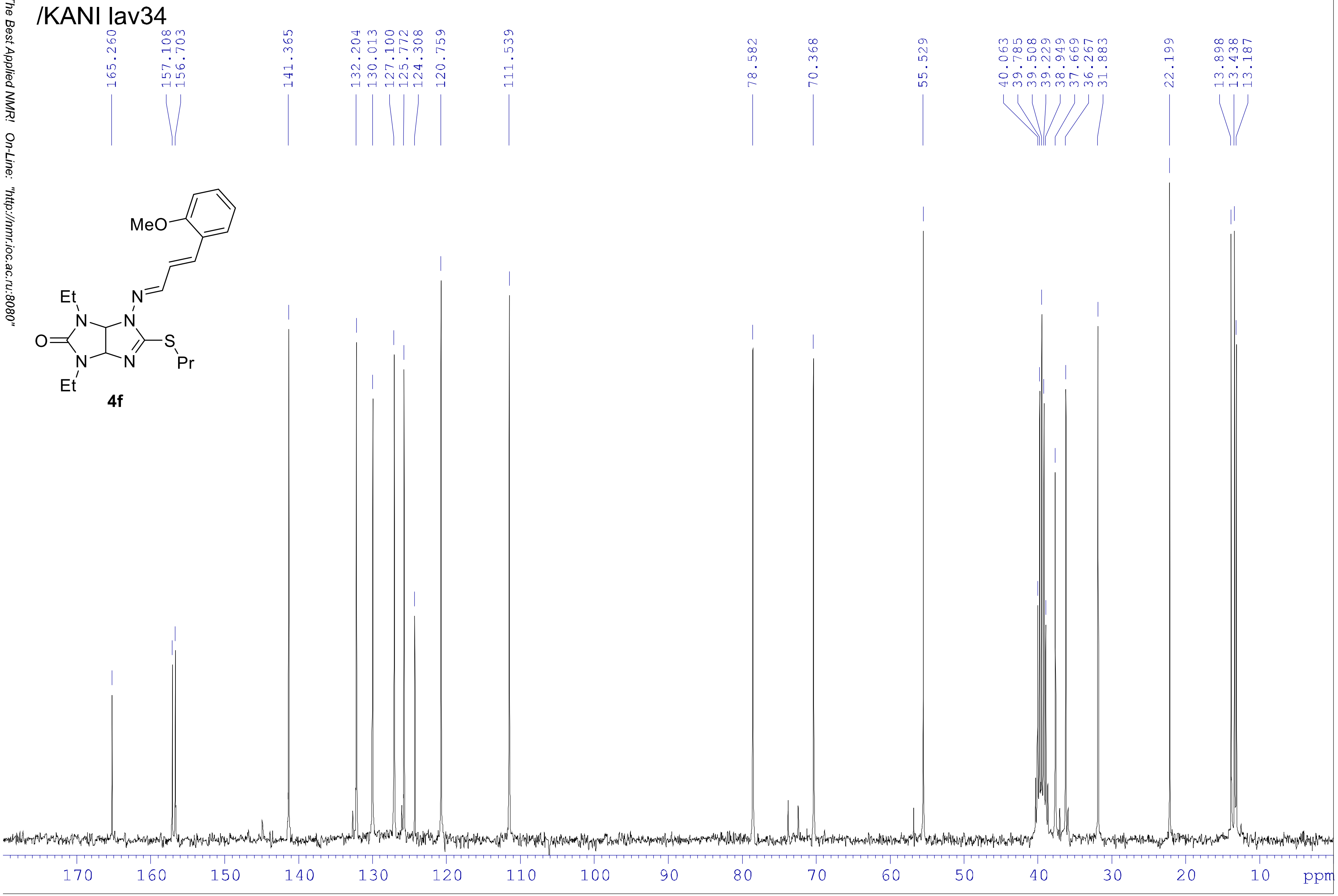

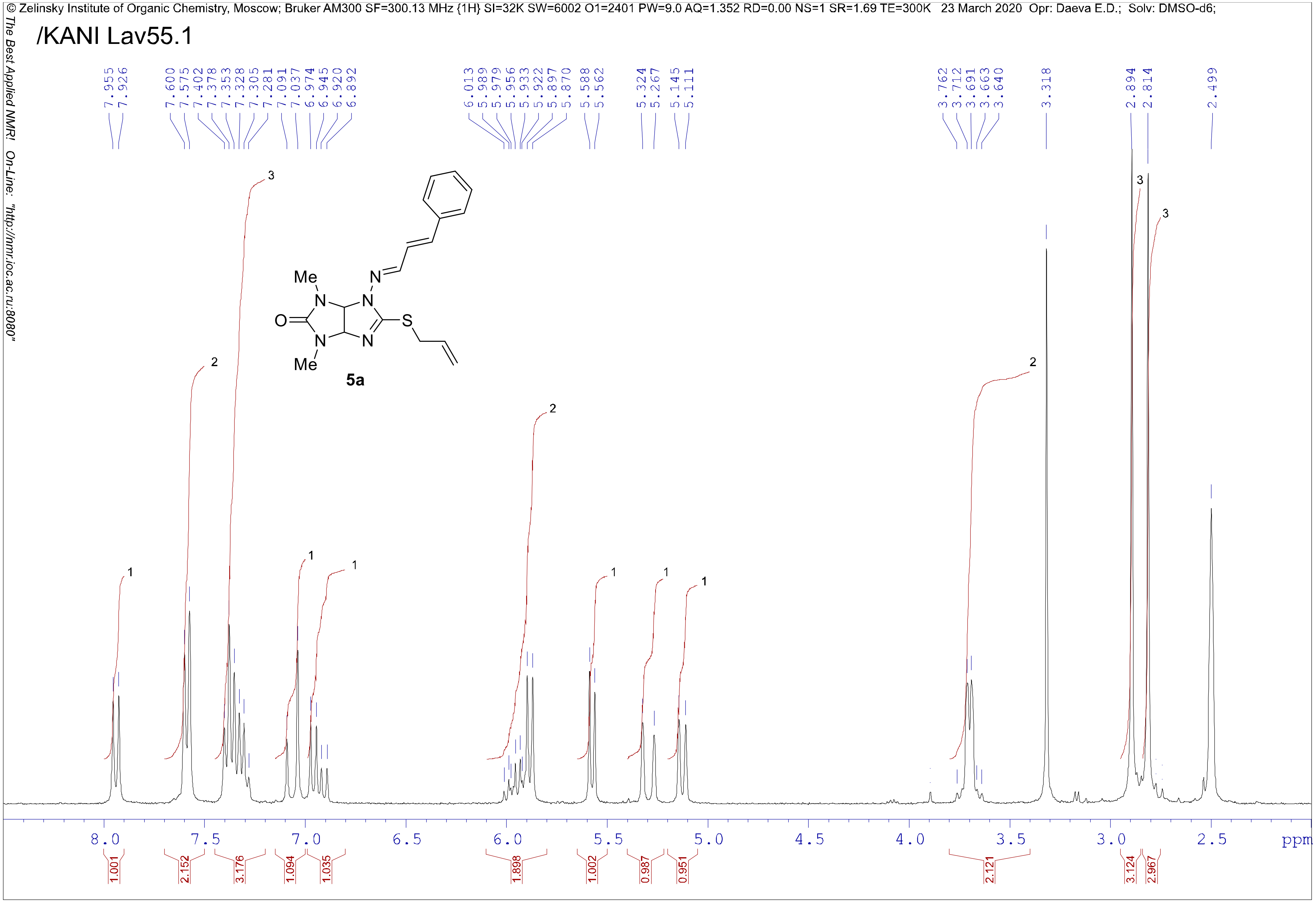
© Zelinsky Institute of Organic Chemistry, Moscow; Bruker AM300 SF=300.13 MHz {1H} SI=32K SW=6002 O1=2401 PW=9.0 AQ=1.352 RD=0.00 NS=1 SR=1.69 TE=300K 23 March 2020 Opr: Daeva E.D.; Solv: DMSO-d6;
/KANI Lav55.1
The Best Applied NMR! On-Line: "http://nmr.ioc.ac.ru:8080"
7.955 7.926 7.600 7.575 7.402 7.378 7.353 7.328 7.305 7.281 7.091 7.037 6.974 6.945 6.920 6.892
6.013 5.989 5.979 5.956 5.933 5.922 5.897 5.870 5.588 5.562 5.324 5.267 5.145 5.111
3.762 3.712 3.691 3.663 3.640 3.318 2.894 2.814 2.499
Me
N
O
N
Me
N
N
N
S
5a
8.0 7.5 7.0 6.5 6.0 5.5 5.0 4.5 4.0 3.5 3.0 2.5 ppm
1.001 2.152 3.176 1.094 1.035 1.898 1.002 0.987 0.951 2.121 3.124 2.967

© Zelinsky Institute of Organic Chemistry, Moscow; Bruker AM300 SF=75.47 MHz {13C} SI=32K SW=18114 O1=8301 PW=14.3 AQ=0.900 RD=1.00 NS=243 SR=37.36 TE=299K 24 March 2020 Opr: Daeva E.D.; Solv: DMSO-d6;

The Best Applied NMR! On-Line: "http://nmr.ioc.ac.ru:8080"

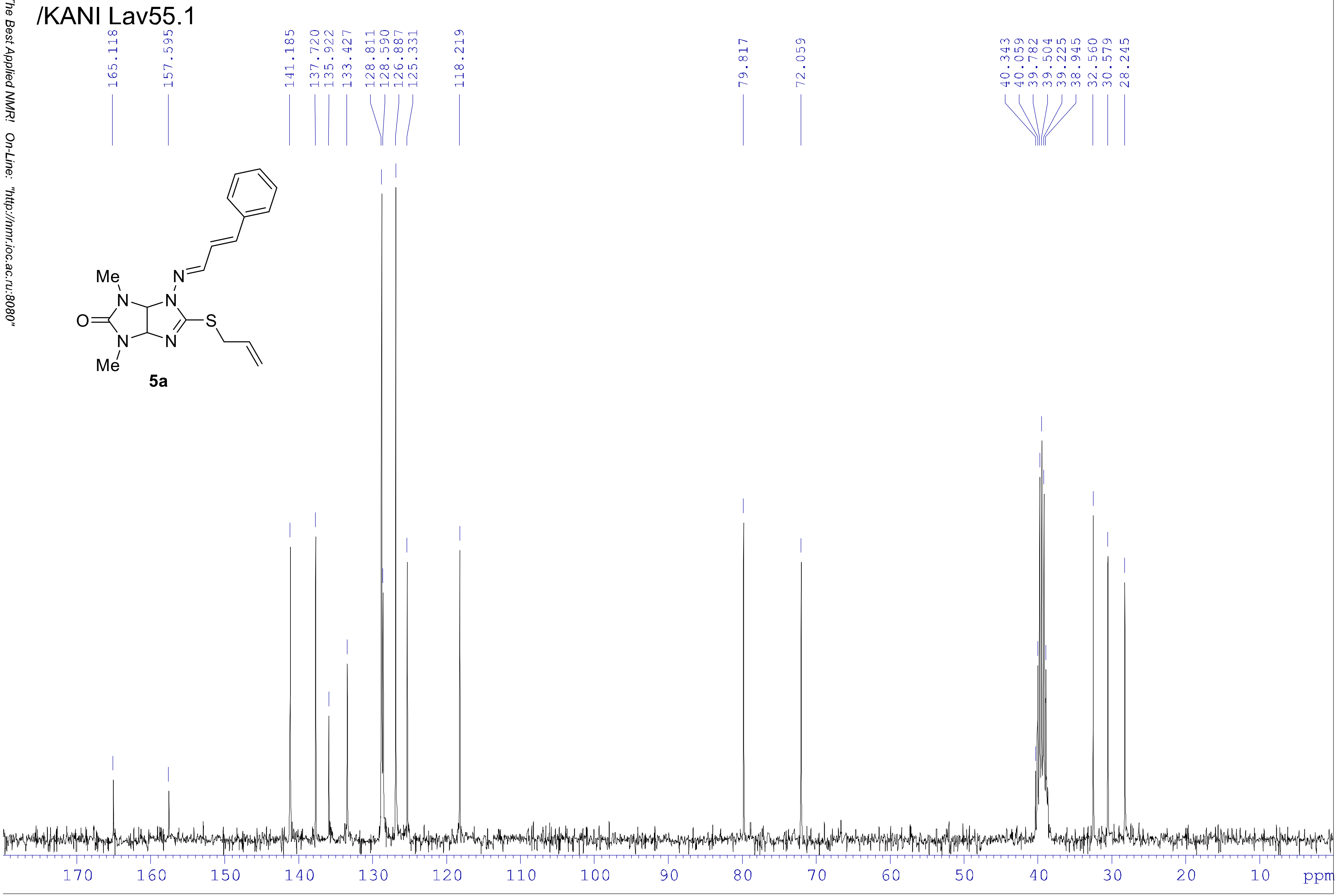

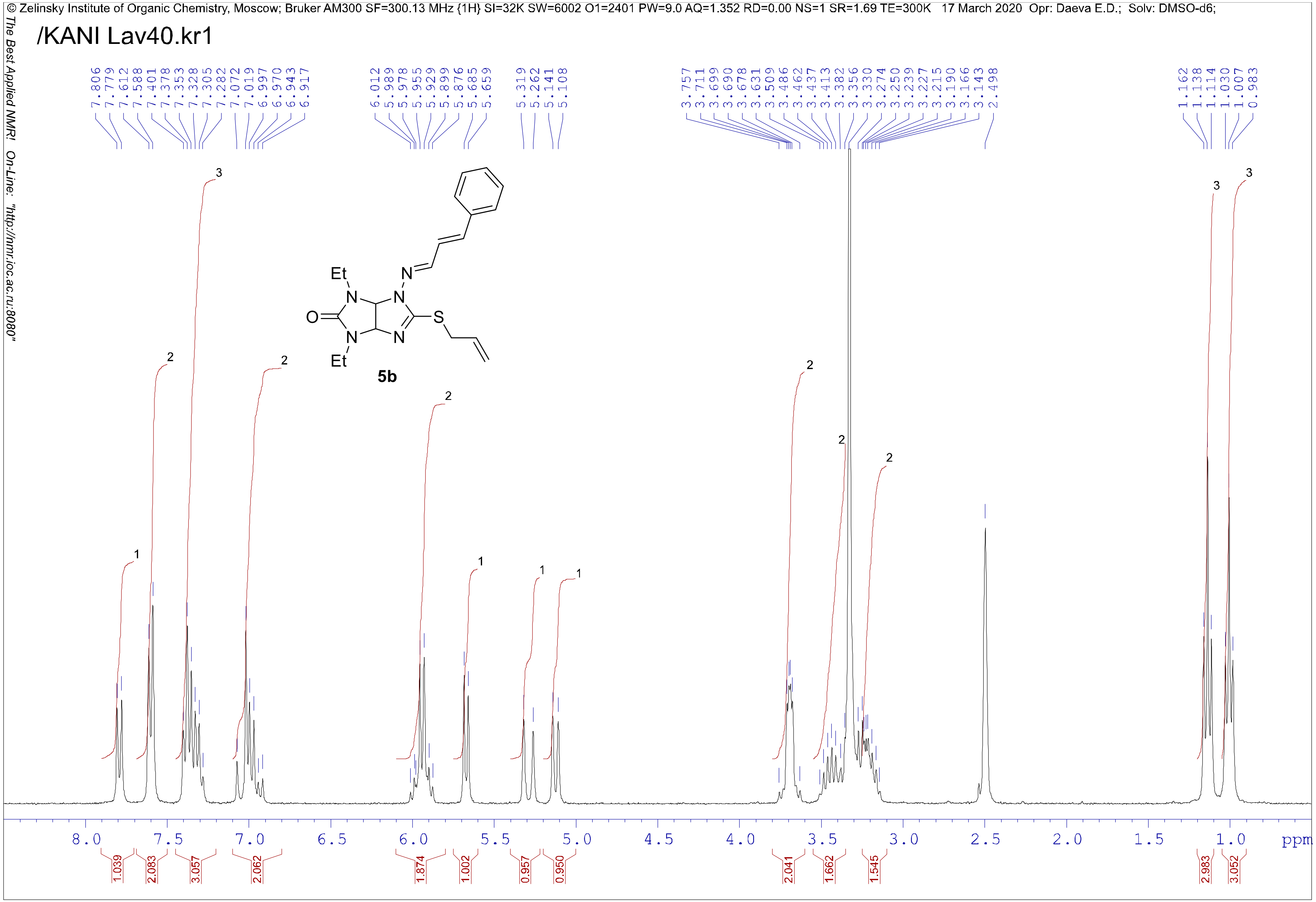
© Zelinsky Institute of Organic Chemistry, Moscow; Bruker AM300 SF=300.13 MHz {1H} SI=32K SW=6002 O1=2401 PW=9.0 AQ=1.352 RD=0.00 NS=1 SR=1.69 TE=300K 17 March 2020 Opr: Daeva E.D.; Solv: DMSO-d6;
/KANI Lav40.kr1
The Best Applied NMR! On-Line: "http://nmr.ioc.ac.ru:8080"
7.806 7.779 7.612 7.588 7.401 7.378 7.353 7.328 7.305 7.282 7.072 7.019 6.997 6.970 6.943 6.917
6.012 5.989 5.978 5.955 5.929 5.899 5.876 5.685 5.659
5.319 5.262 5.141 5.108
3.757 3.711 3.699 3.690 3.678 3.631 3.509 3.486 3.462 3.437 3.413 3.382 3.356 3.330 3.274 3.250 3.239 3.227 3.215 3.190 3.166 3.143 2.498
1.162 1.138 1.114 1.030 1.007 0.983
5b
8.0 7.5 7.0 6.5 6.0 5.5 5.0 4.5 4.0 3.5 3.0 2.5 2.0 1.5 1.0 ppm
1.039 2.083 3.057 2.062 1.874 1.002 0.957 0.950 2.041 1.662 1.545 2.983 3.052

© Zelinsky Institute of Organic Chemistry, Moscow; Bruker AM300 SF=75.47 MHz {13C} SI=32K SW=18114 O1=8301 PW=14.3 AQ=0.900 RD=1.00 NS=197 SR=38.01 TE=300K 18 March 2020 Opr: Daeva E.D.; Solv: DMSO-d6;

The Best Applied NMR! On-Line: "http://nmr.ioc.ac.ru:8080"

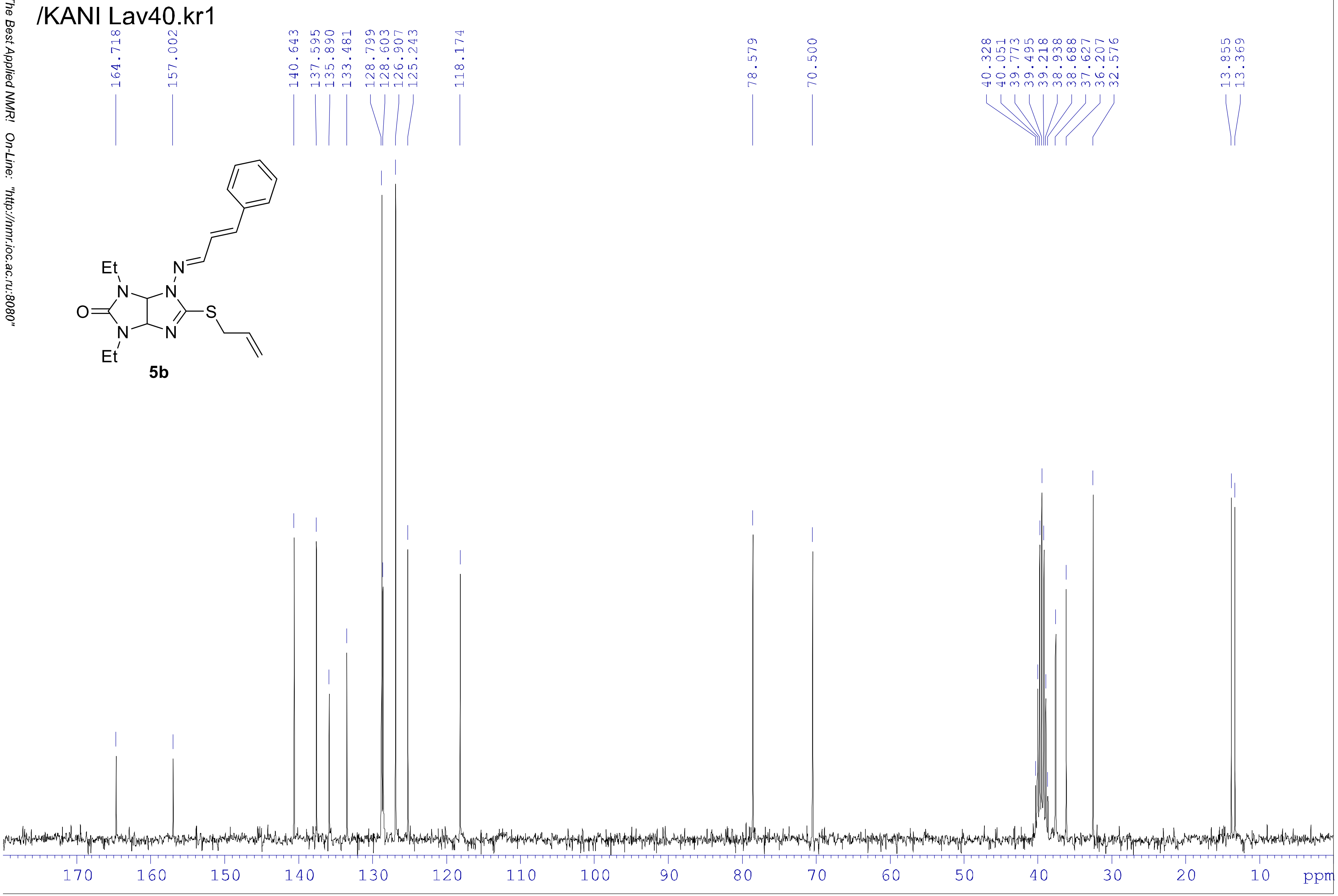

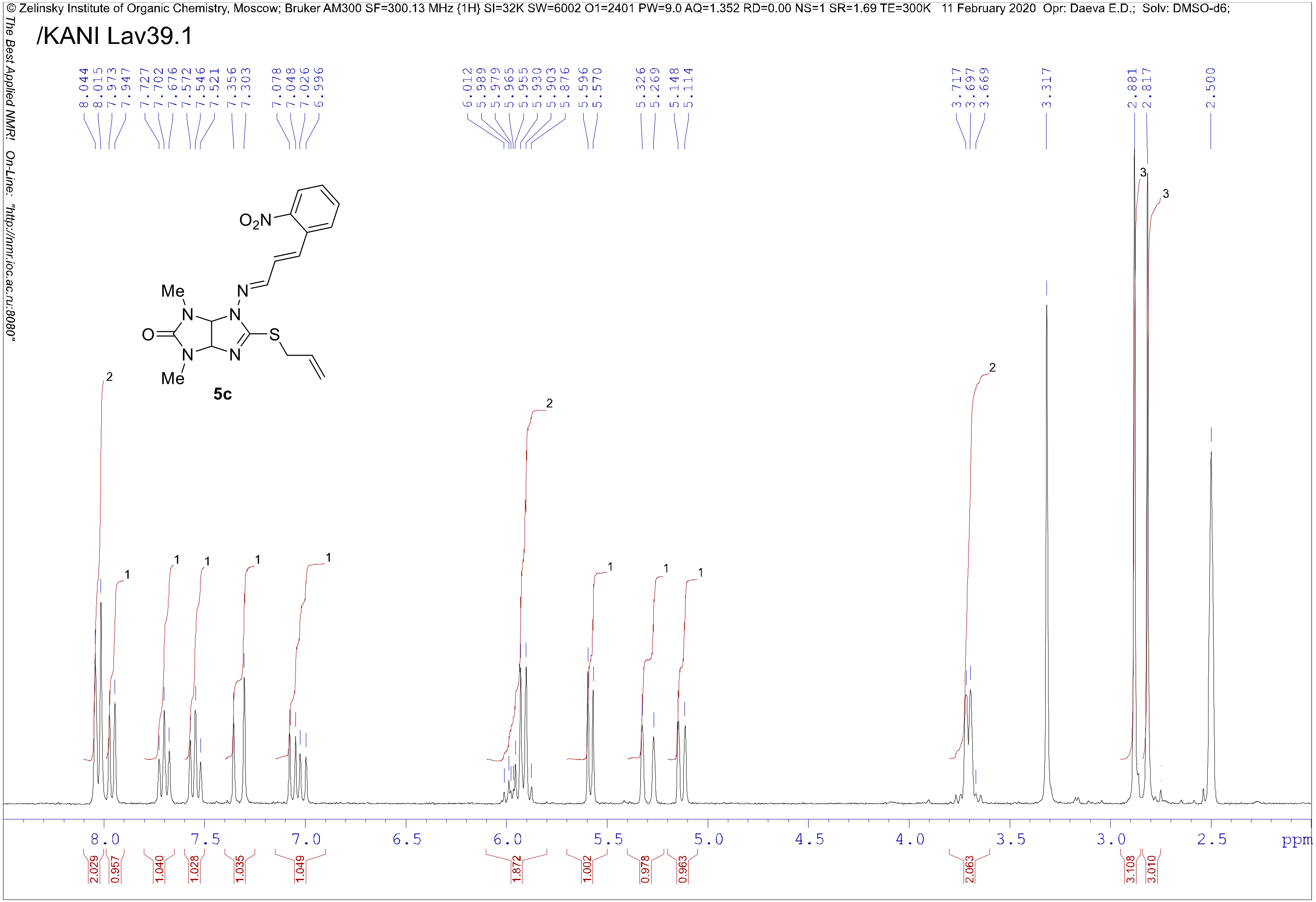
© Zelinsky Institute of Organic Chemistry, Moscow; Bruker AM300 SF=300.13 MHz {1H} SI=32K SW=6002 O1=2401 PW=9.0 AQ=1.352 RD=0.00 NS=1 SR=1.69 TE=300K 11 February 2020 Opr: Daeva E.D.; Solv: DMSO-d6;
The Best Applied NMR! On-Line: "http://nmr.ioc.ac.ru:8080"
/KANI Lav39.1
8.044 8.015 7.973 7.947 7.727 7.702 7.676 7.572 7.546 7.521 7.356 7.303 7.078 7.048 7.026 6.996
6.012 5.989 5.979 5.965 5.955 5.930 5.903 5.876 5.596 5.570 5.326 5.269 5.148 5.114
3.717 3.697 3.669 3.317 2.881 2.817 2.500
O_2N
Me
N
O
S
5c
ppm
8.0 7.5 7.0 6.5 6.0 5.5 5.0 4.5 4.0 3.5 3.0 2.5
2.029 0.957 1.040 1.028 1.035 1.049 1.872 1.002 0.978 0.963 2.063 3.108 3.010

© Zelinsky Institute of Organic Chemistry, Moscow; Bruker AM300 SF=75.47 MHz {13C} SI=32K SW=18114 O1=8301 PW=14.3 AQ=0.900 RD=1.00 NS=291 SR=37.58 TE=300K 12 February 2020 Opr: Daeva E.D.; Solv: DMSO-d6;

The Best Applied NMR! On-Line: "http://nmr.ioc.ac.ru:8080"

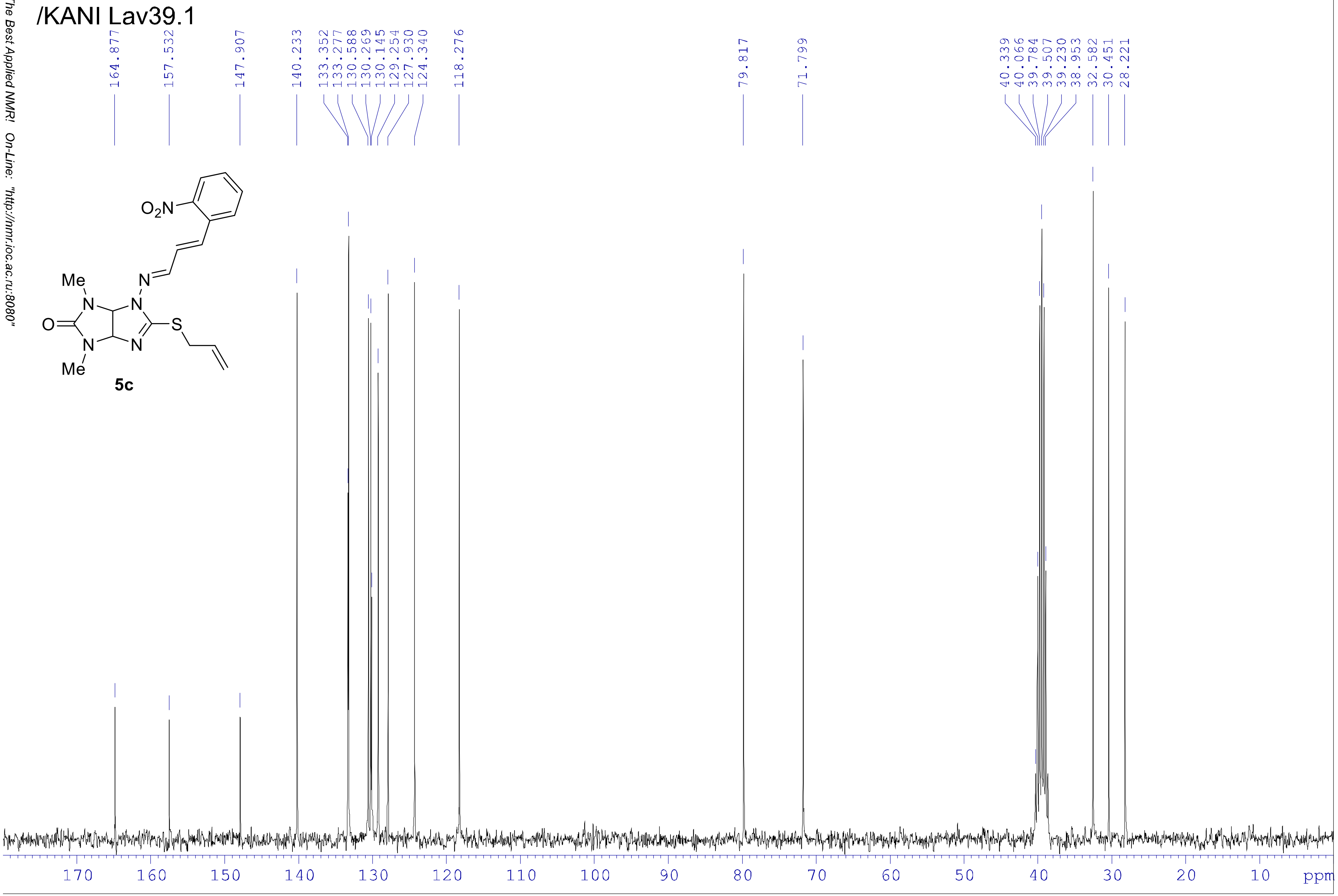

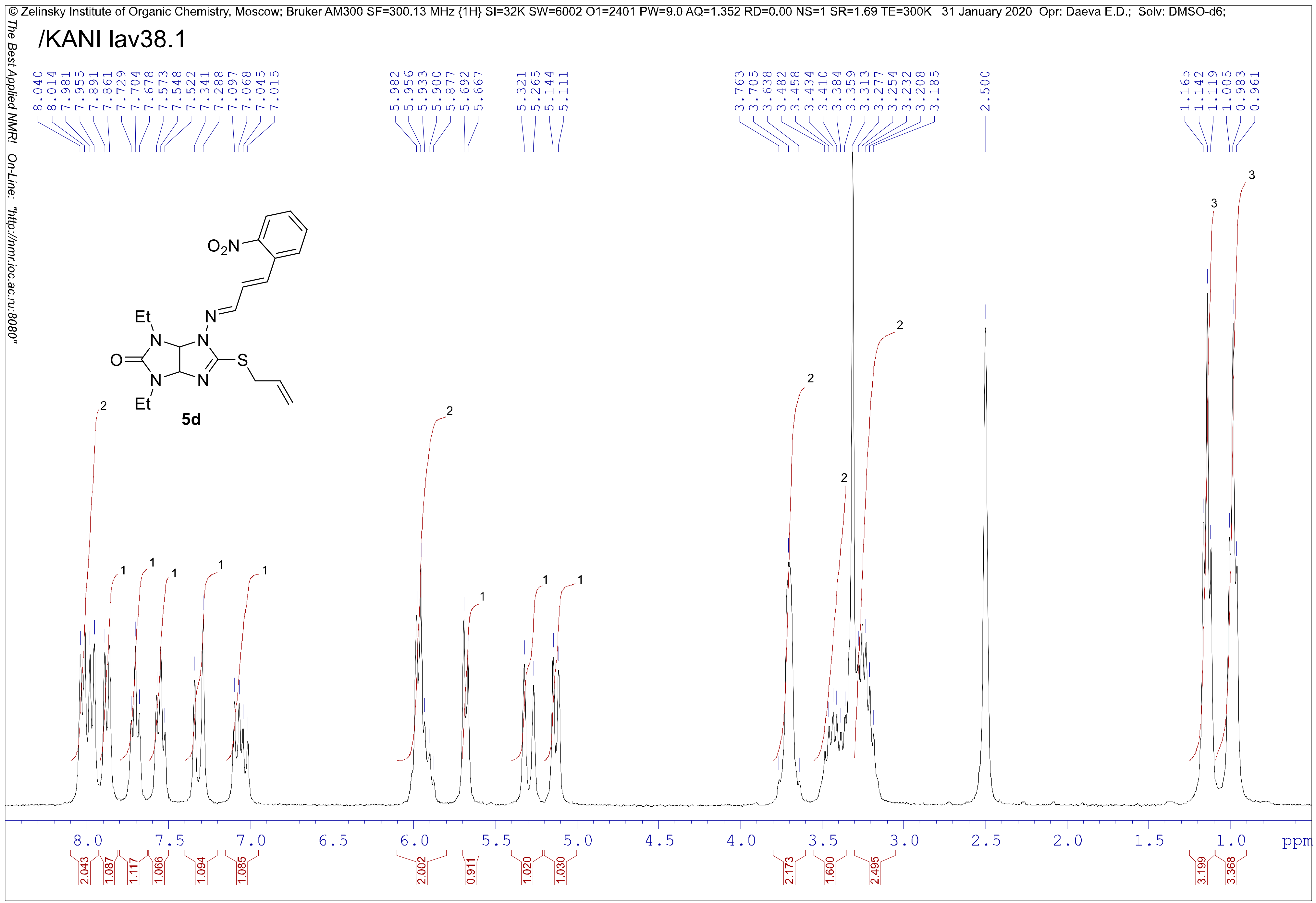
© Zelinsky Institute of Organic Chemistry, Moscow; Bruker AM300 SF=300.13 MHz {1H} SI=32K SW=6002 O1=2401 PW=9.0 AQ=1.352 RD=0.00 NS=1 SR=1.69 TE=300K 31 January 2020 Opr: Daeva E.D.; Solv: DMSO-d6;
/KANI lav38.1
The Best Applied NMR! On-Line: "http://nmr.ioc.ac.ru:8080"
8.040 8.014 7.981 7.955 7.891 7.861 7.729 7.704 7.678 7.573 7.548 7.522 7.341 7.288 7.097 7.068 7.045 7.015
5.982 5.956 5.933 5.900 5.877 5.692 5.667
5.321 5.265 5.144 5.111
3.763 3.705 3.638 3.482 3.458 3.434 3.410 3.384 3.359 3.313 3.277 3.254 3.232 3.208 3.185
2.500
1.165 1.142 1.119 1.005 0.983 0.961
O_2N
Et
N
N
O
N
N
S
N
Et
5d
8.0 7.5 7.0 6.5 6.0 5.5 5.0 4.5 4.0 3.5 3.0 2.5 2.0 1.5 1.0 ppm
2.043 1.087 1.117 1.066 1.094 1.085 2.002 0.911 1.020 1.030 2.173 1.600 2.495 3.199 3.368

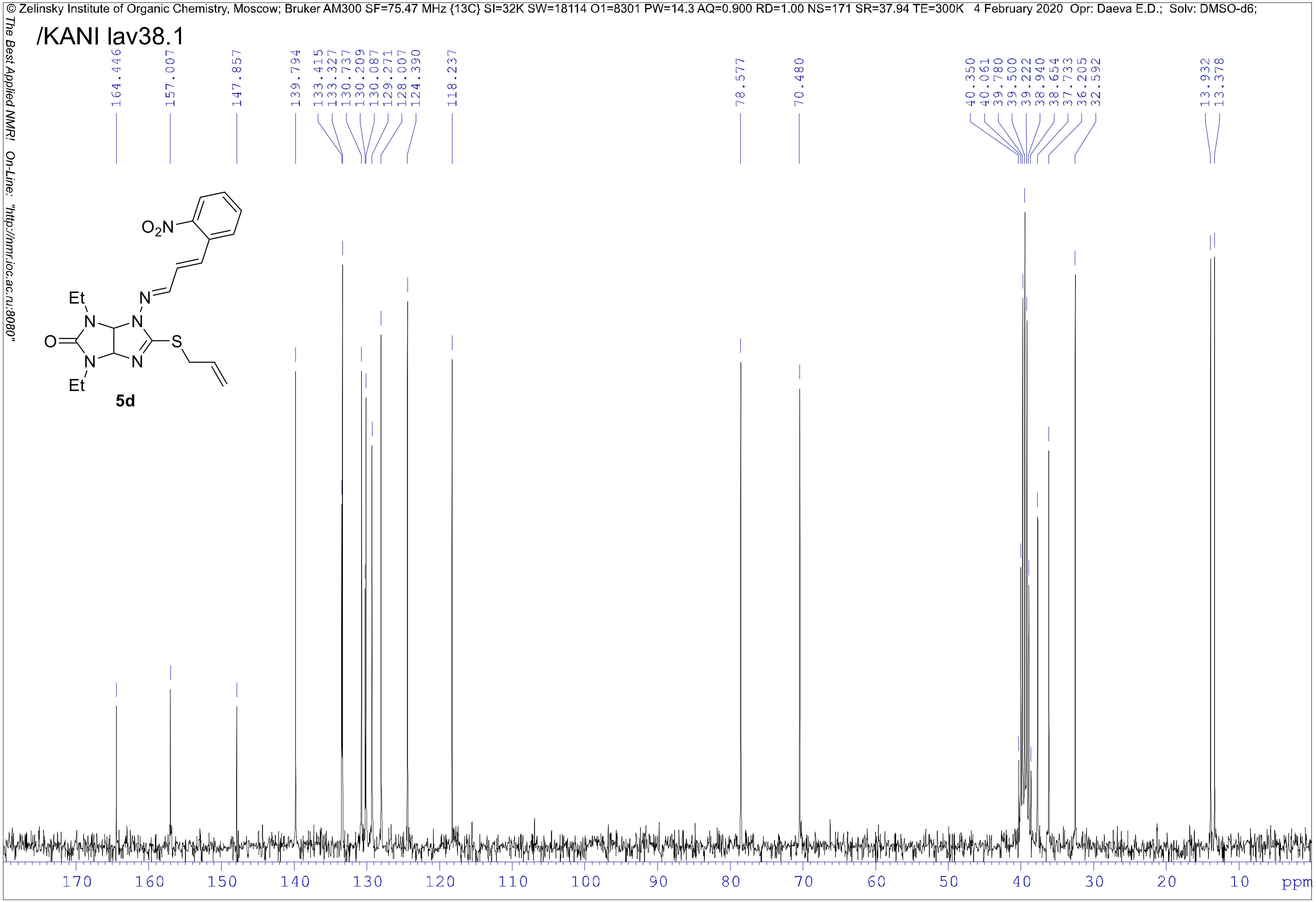

© Zelinsky Institute of Organic Chemistry, Moscow; Bruker AM300 SF=75.47 MHz {13C} SI=32K SW=18114 O1=8301 PW=14.3 AQ=0.900 RD=1.00 NS=171 SR=37.94 TE=300K 4 February 2020 Opr: Daeva E.D.; Solv: DMSO-d6;
/KANI lav38.1
The Best Applied NMR! On-Line: "http://nmr.ioc.ac.ru:8080"
164.446
157.007
147.857
139.794
133.415
133.327
130.737
130.209
130.087
129.271
128.007
124.390
118.237
78.577
70.480
40.350
40.061
39.780
39.500
39.222
38.940
38.654
37.733
36.205
32.592
13.932
13.378
O_2N
Et
N
N
O
N
N
S
Et
5d
170
160
150
140
130
120
110
100
90
80
70
60
50
40
30
20
10
ppm

© Zelinsky Institute of Organic Chemistry, Moscow; Bruker AM300 SF=300.13 MHz {1H} SI=32K SW=6002 O1=2401 PW=9.0 AQ=1.352 RD=0.00 NS=1 SR=1.69 TE=300K 10 March 2020 Opr: Daeva E.D.; Solv: DMSO-d6;

The Best Applied NMR! On-Line: "http://nmr.ioc.ac.ru:8080"

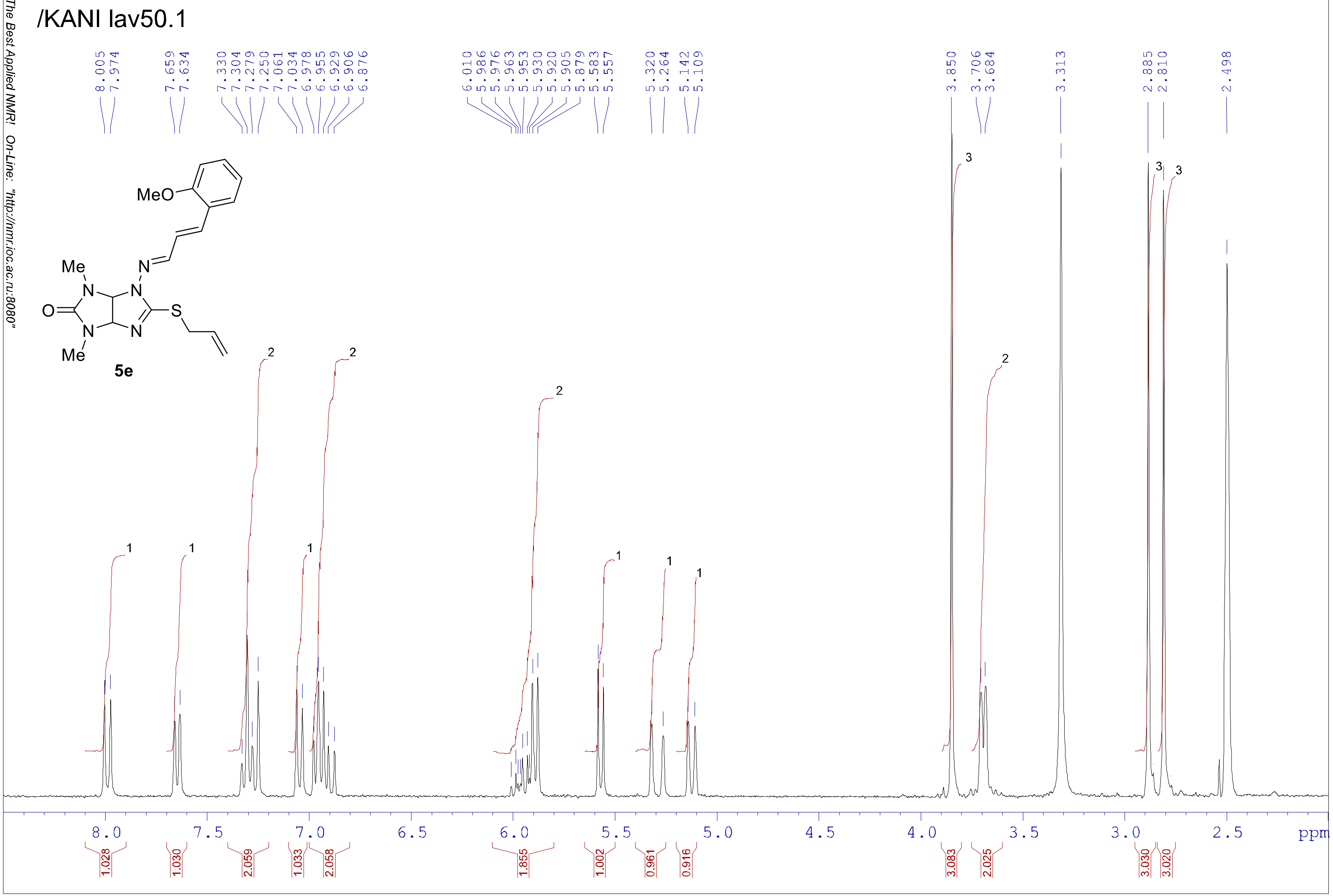

© Zelinsky Institute of Organic Chemistry, Moscow; Bruker AM300 SF=75.47 MHz {13C} SI=32K SW=18114 O1=8301 PW=14.3 AQ=0.900 RD=1.00 NS=130 SR=37.76 TE=301K 11 March 2020 Opr: Daeva E.D.; Solv: DMSO-d6;

The Best Applied NMR! On-Line: "http://nmr.ioc.ac.ru:8080"

/KANI lav50.1

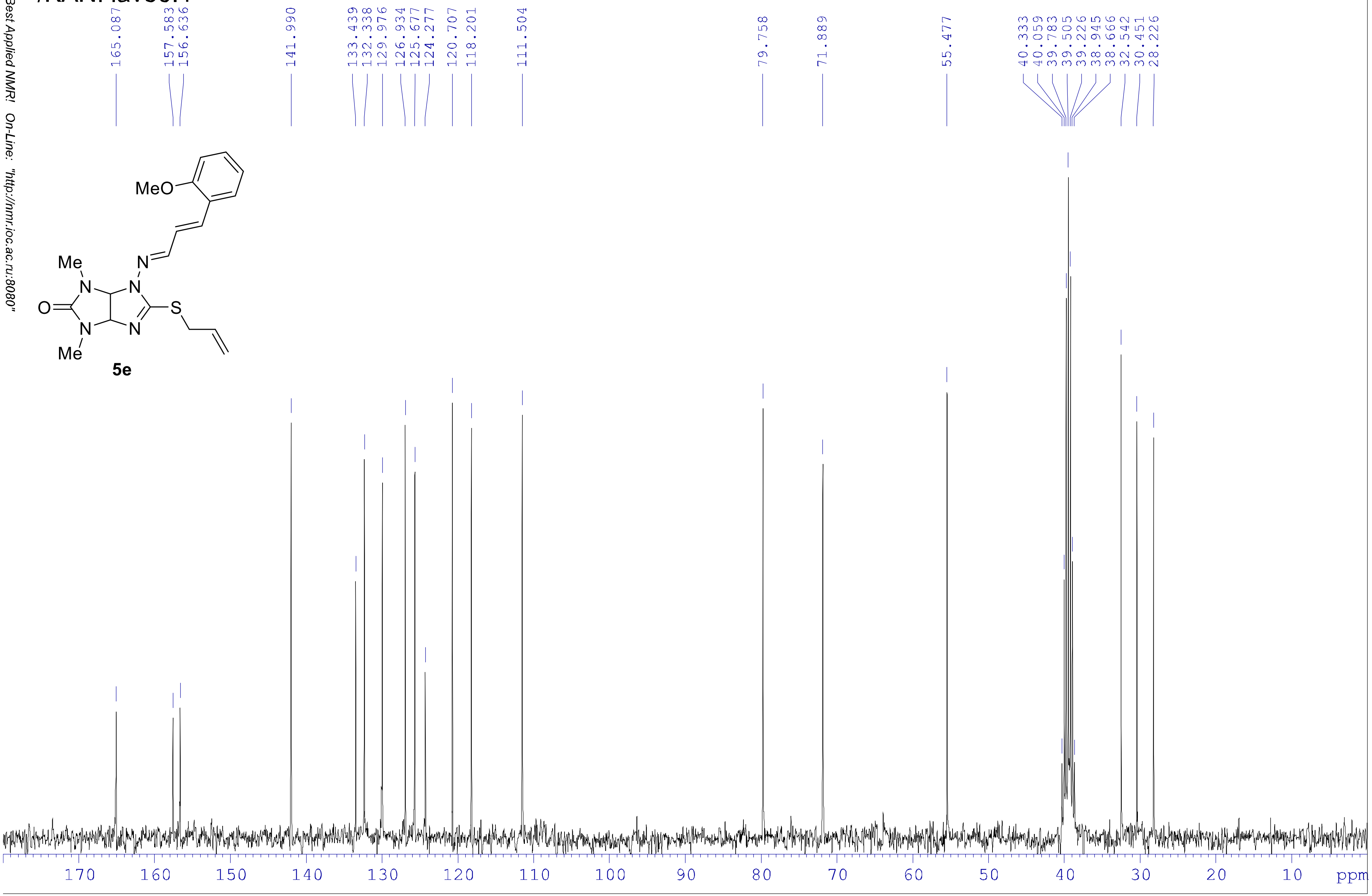

5e

© Zelinsky Institute of Organic Chemistry, Moscow; Bruker AM300 SF=300.13 MHz {1H} SI=32K SW=6002 O1=2401 PW=9.0 AQ=1.352 RD=0.00 NS=1 SR=1.69 TE=300K 10 March 2020 Opr: Daeva E.D.; Solv: DMSO-d6;

/KANI lav51.1

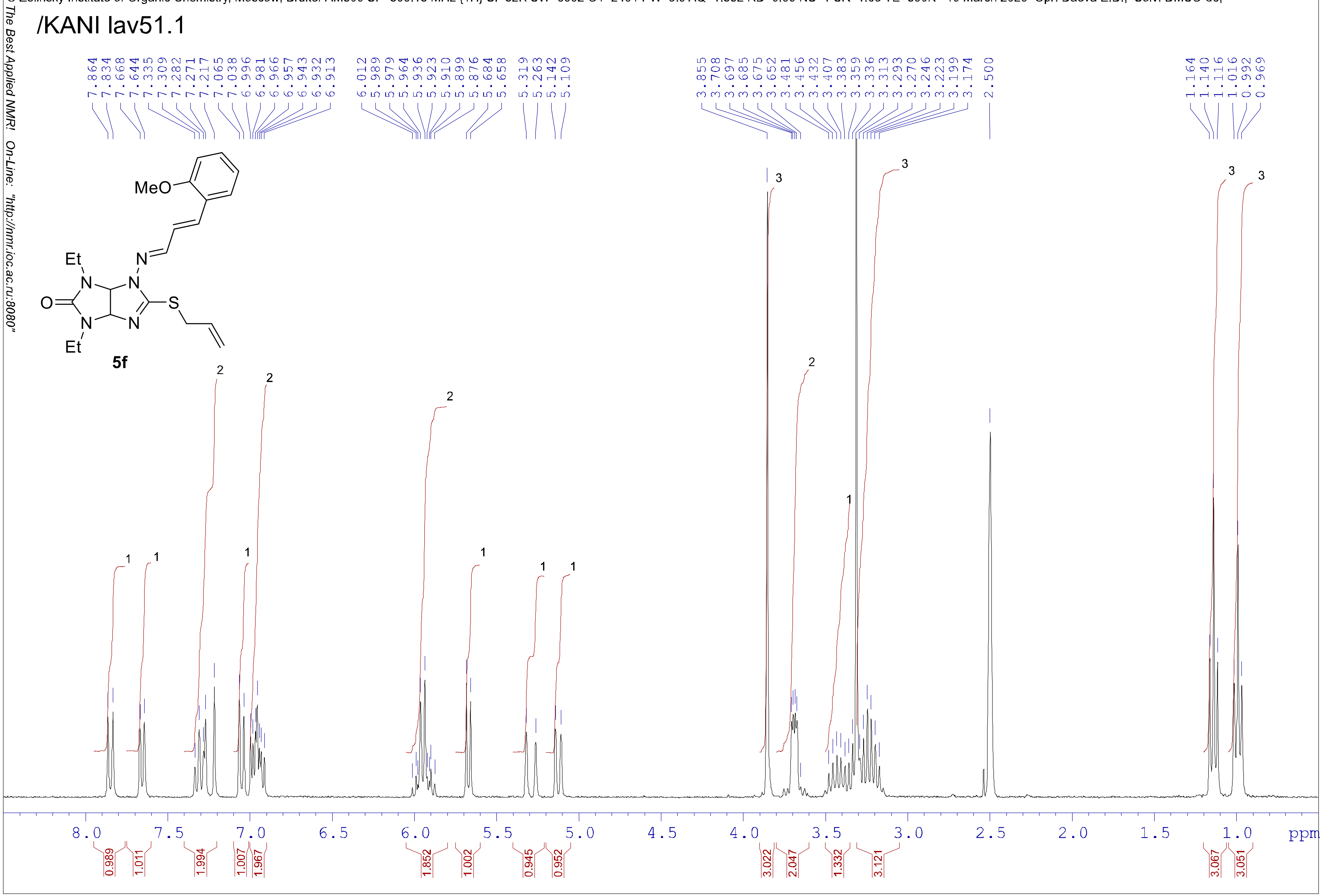

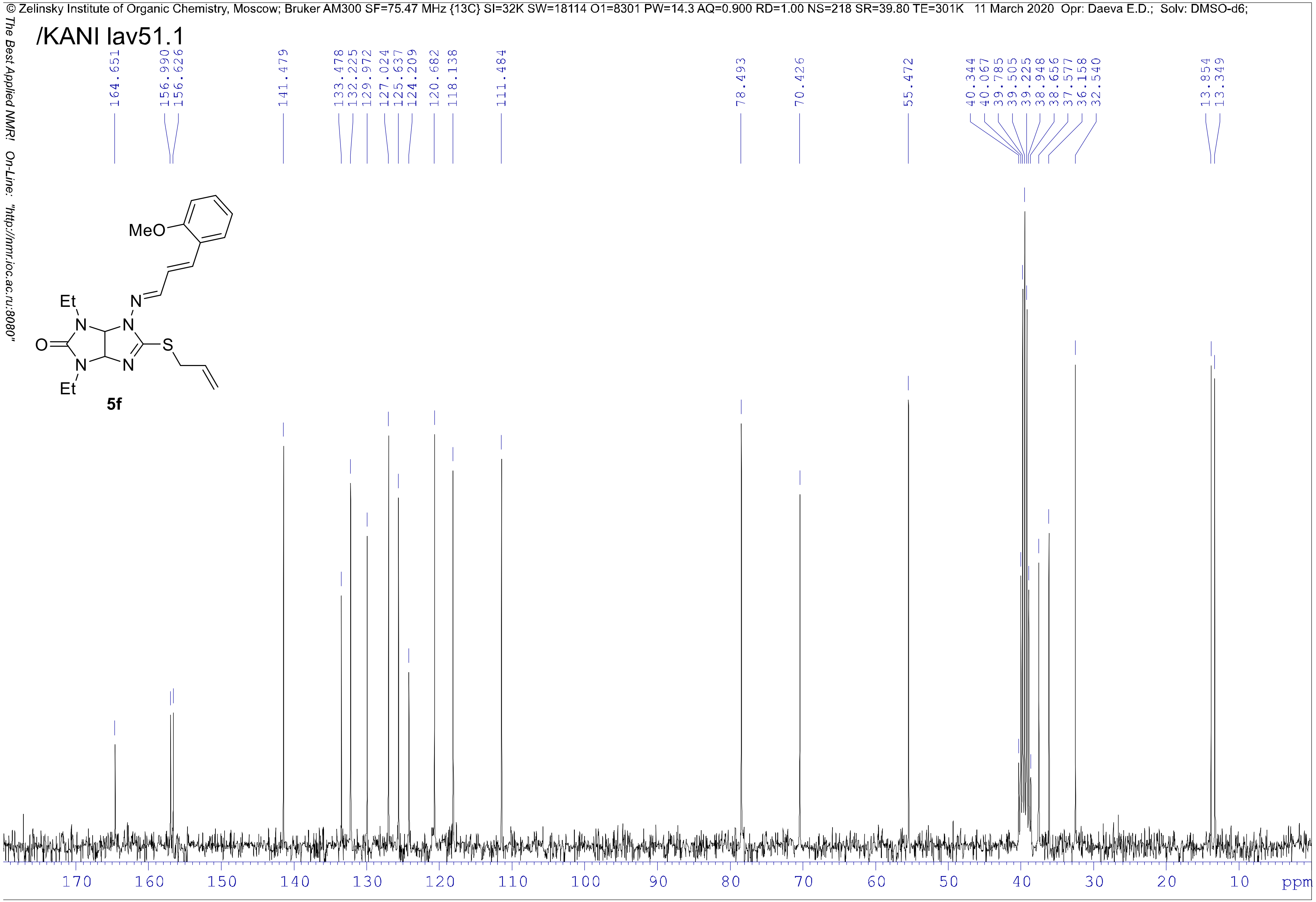
© Zelinsky Institute of Organic Chemistry, Moscow; Bruker AM300 SF=75.47 MHz {13C} SI=32K SW=18114 O1=8301 PW=14.3 AQ=0.900 RD=1.00 NS=218 SR=39.80 TE=301K 11 March 2020 Opr: Daeva E.D.; Solv: DMSO-d6;
The Best Applied NMR! On-Line: "http://nmr.ioc.ac.ru:8080"
/KANI lav51.1
164.651
156.990
156.626
141.479
133.478
132.225
129.972
127.024
125.637
124.209
120.682
118.138
111.484
78.493
70.426
55.472
40.344
40.067
39.785
39.505
39.225
38.948
38.656
37.577
36.158
32.540
13.854
13.349
MeO
Et
N
O
S
5f
170 160 150 140 130 120 110 100 90 80 70 60 50 40 30 20 10 ppm

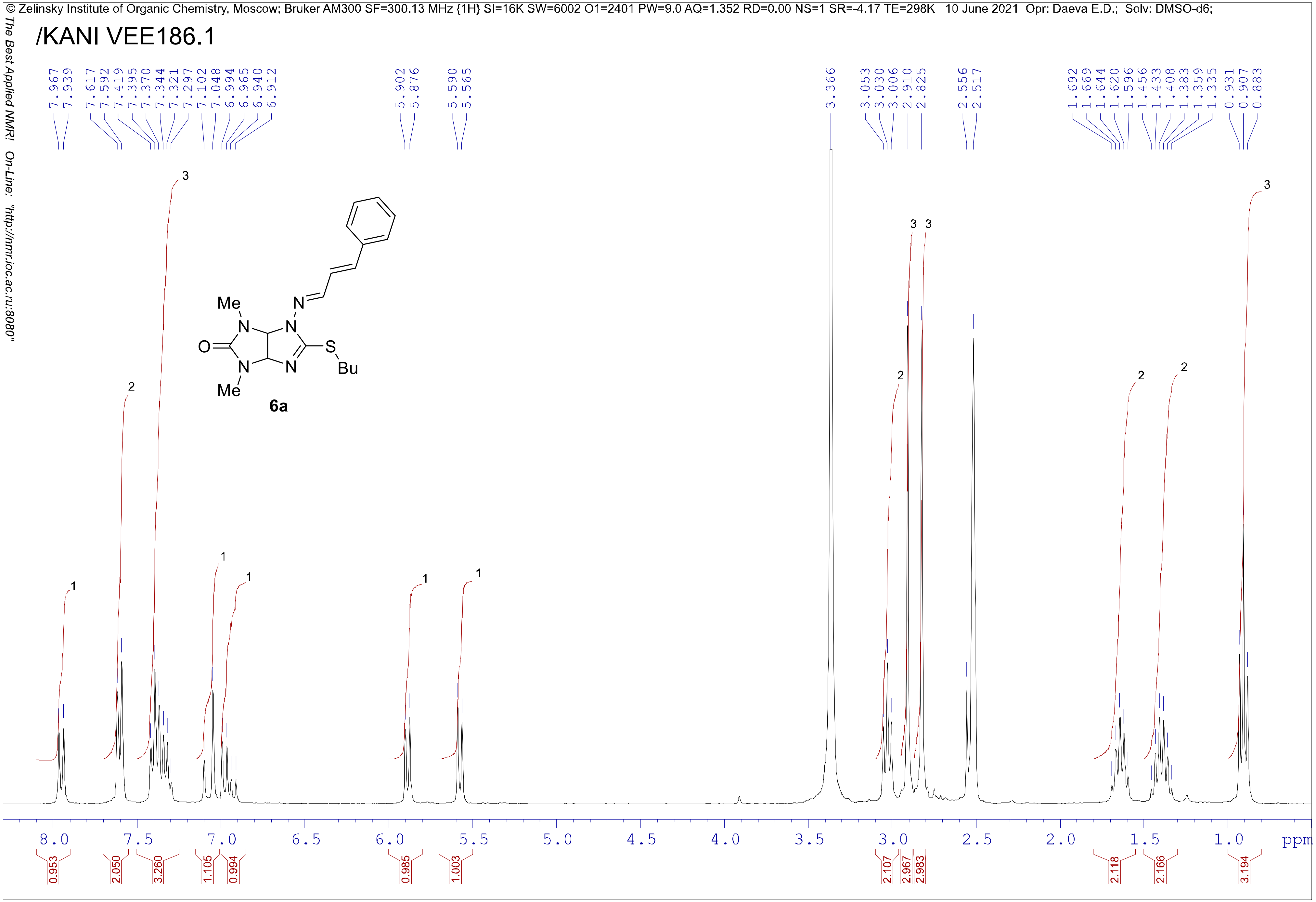
© Zelinsky Institute of Organic Chemistry, Moscow; Bruker AM300 SF=300.13 MHz {1H} SI=16K SW=6002 O1=2401 PW=9.0 AQ=1.352 RD=0.00 NS=1 SR=-4.17 TE=298K 10 June 2021 Opr: Daeva E.D.; Solv: DMSO-d6;
/KANI VEE186.1
The Best Applied NMR! On-Line: "http://nmr.ioc.ac.ru:8080"
6a
ppm

© Zelinsky Institute of Organic Chemistry, Moscow; Bruker AM300 SF=75.47 MHz {13C} SI=32K SW=18114 O1=8301 PW=14.3 AQ=0.901 RD=1.00 NS=215 SR=36.41 TE=297K 11 June 2021 Opr: Daeva E.D.; Solv: DMSO-d6;

The Best Applied NMR! On-Line: "http://nmr.ioc.ac.ru:8080"

/KANI VEE186.1

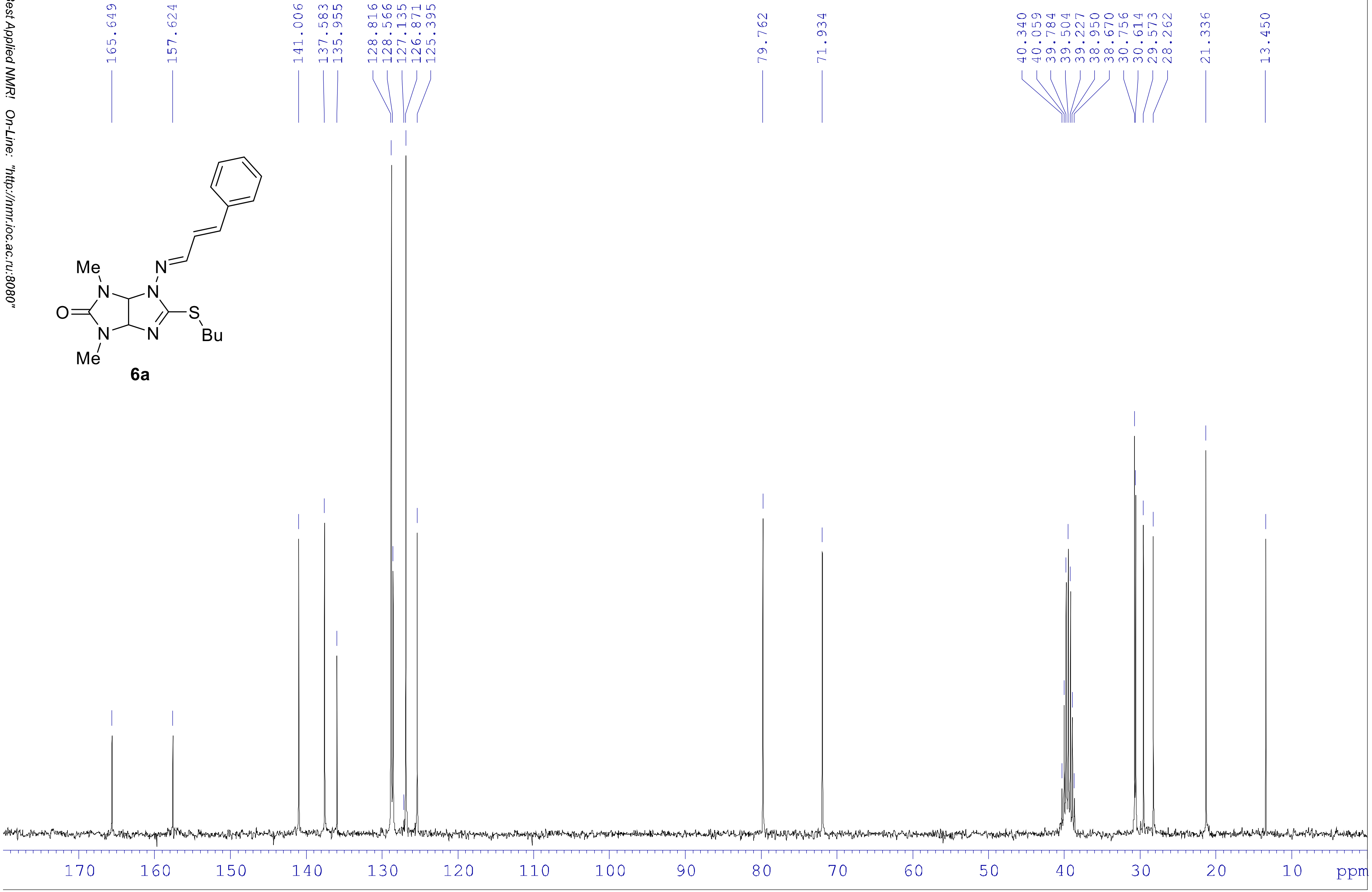

© Zelinsky Institute of Organic Chemistry, Moscow; Bruker AV300 SF=300.13 MHz {1H} SI=64K SW=6010 O1=2101 PW=15.0 AQ=2.715 RD=0.10 NS=1 SR=0.00 TE=300K 26 July 2021 Opr: NMR User; Solv: DMSO-d6;

The Best Applied NMR! On-Line: "http://nmr.ioc.ac.ru:8080"

/KANI VEE207.1 Gazieva-00765

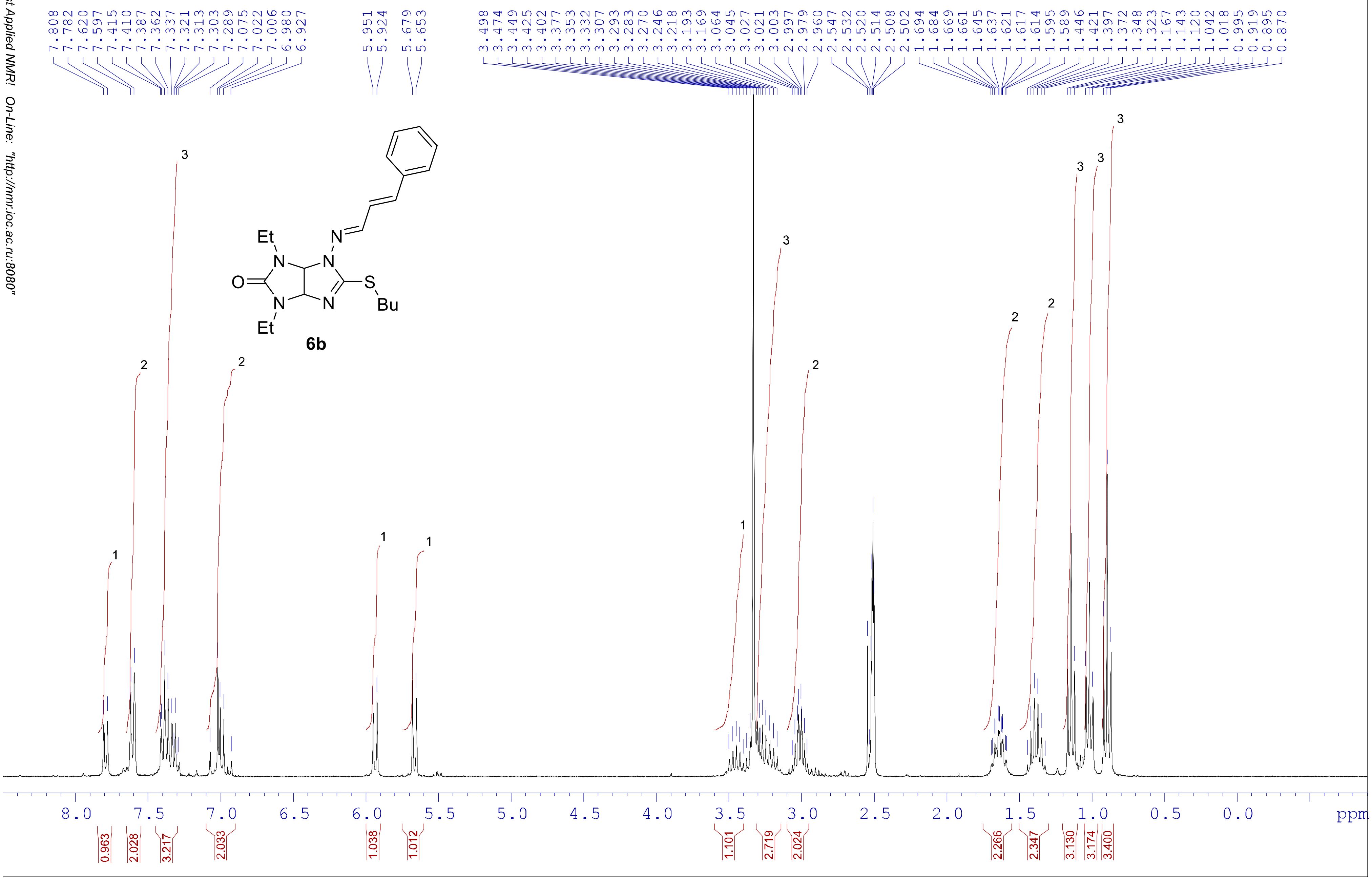

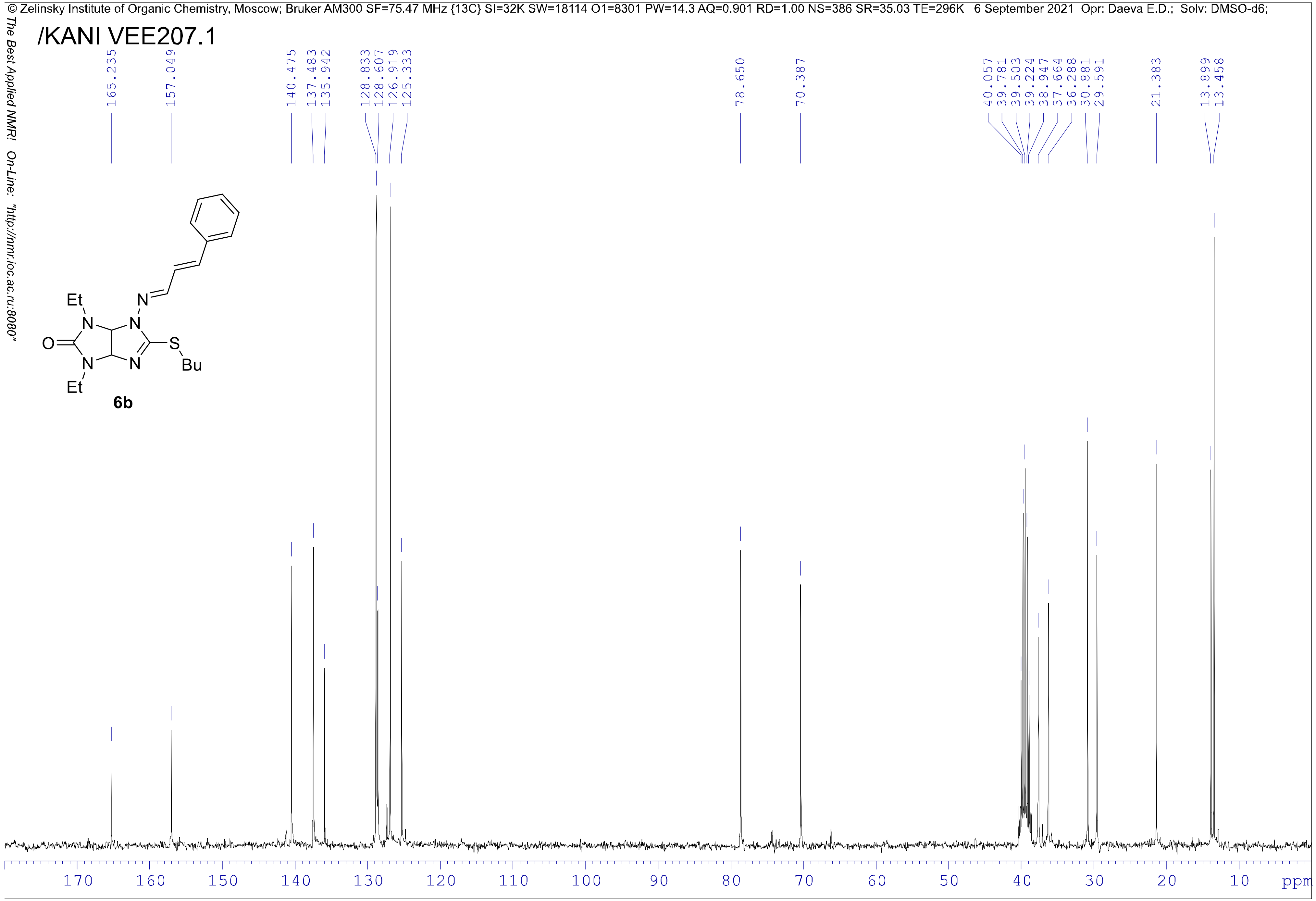
© Zelinsky Institute of Organic Chemistry, Moscow; Bruker AM300 SF=75.47 MHz {13C} SI=32K SW=18114 O1=8301 PW=14.3 AQ=0.901 RD=1.00 NS=386 SR=35.03 TE=296K 6 September 2021 Opr: Daeva E.D.; Solv: DMSO-d6;
The Best Applied NMR! On-Line: "http://nmr.ioc.ac.ru:8080"
/KANI VEE207.1
165.235
157.049
140.475
137.483
135.942
128.833
128.607
126.919
125.333
78.650
70.387
40.057
39.781
39.503
39.224
38.947
37.664
36.288
30.881
29.591
21.383
13.899
13.458
Et
N
N
N
O
S
Bu
N
Et
6b
170
160
150
140
130
120
110
100
90
80
70
60
50
40
30
20
10
ppm

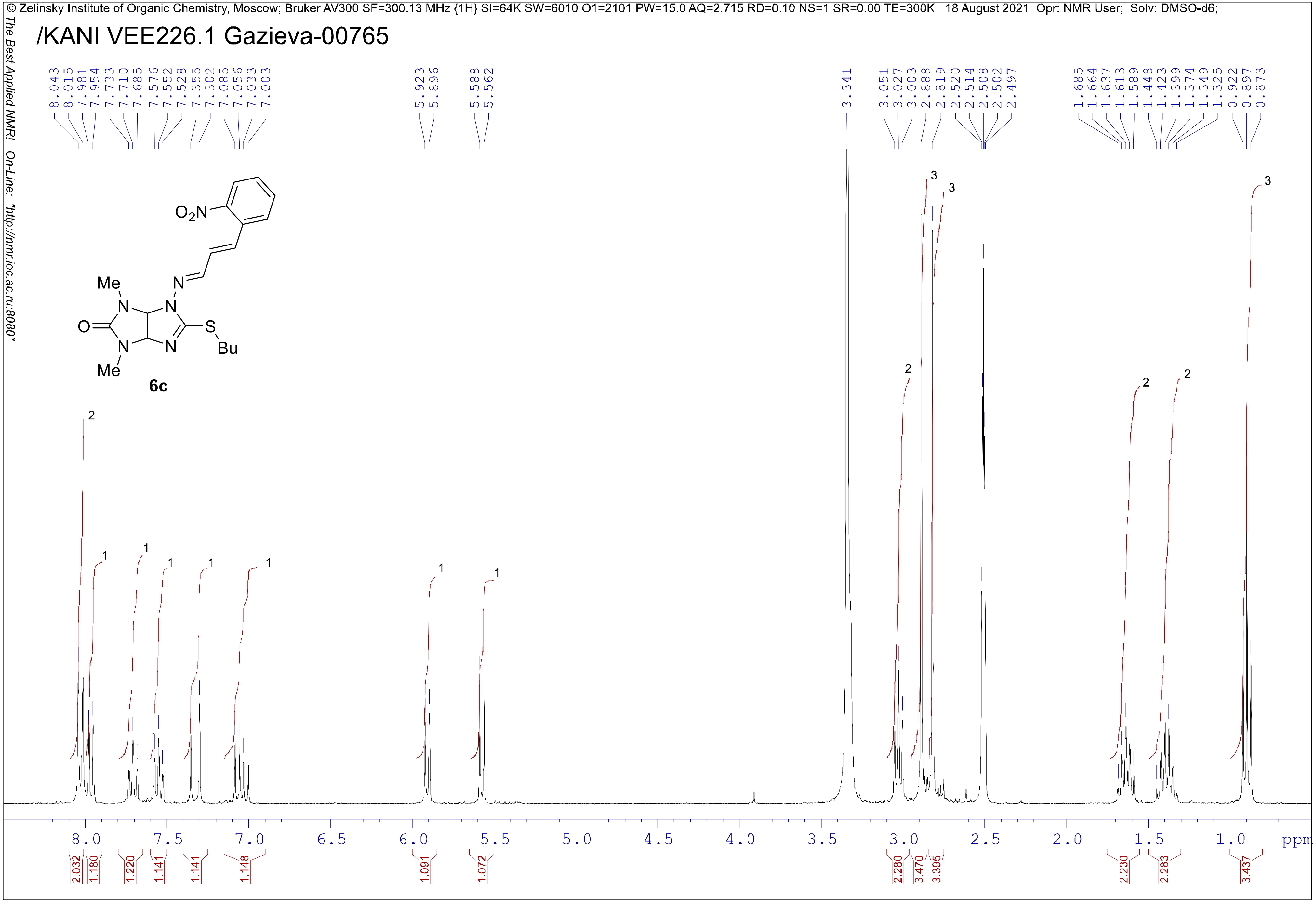
© Zelinsky Institute of Organic Chemistry, Moscow; Bruker AV300 SF=300.13 MHz {1H} SI=64K SW=6010 O1=2101 PW=15.0 AQ=2.715 RD=0.10 NS=1 SR=0.00 TE=300K 18 August 2021 Opr: NMR User; Solv: DMSO-d6;
/KANI VEE226.1 Gazieva-00765
The Best Applied NMR! On-Line: "http://nmr.ioc.ac.ru:8080"
8.043 8.015 7.981 7.954 7.733 7.710 7.685 7.576 7.552 7.528 7.355 7.302 7.085 7.056 7.033 7.003
5.923 5.896
5.588 5.562
3.341
3.051 3.027 3.003 2.888 2.819 2.520 2.514 2.508 2.502 2.497
1.685 1.664 1.637 1.613 1.589 1.448 1.423 1.399 1.374 1.349 1.325 0.922 0.897 0.873
O_2N
Me
N
O
S
Bu
6c
2.032 1.180 1.220 1.141 1.141 1.148 1.091 1.072 2.280 3.470 3.395 2.230 2.283 3.437
8.0 7.5 7.0 6.5 6.0 5.5 5.0 4.5 4.0 3.5 3.0 2.5 2.0 1.5 1.0 ppm

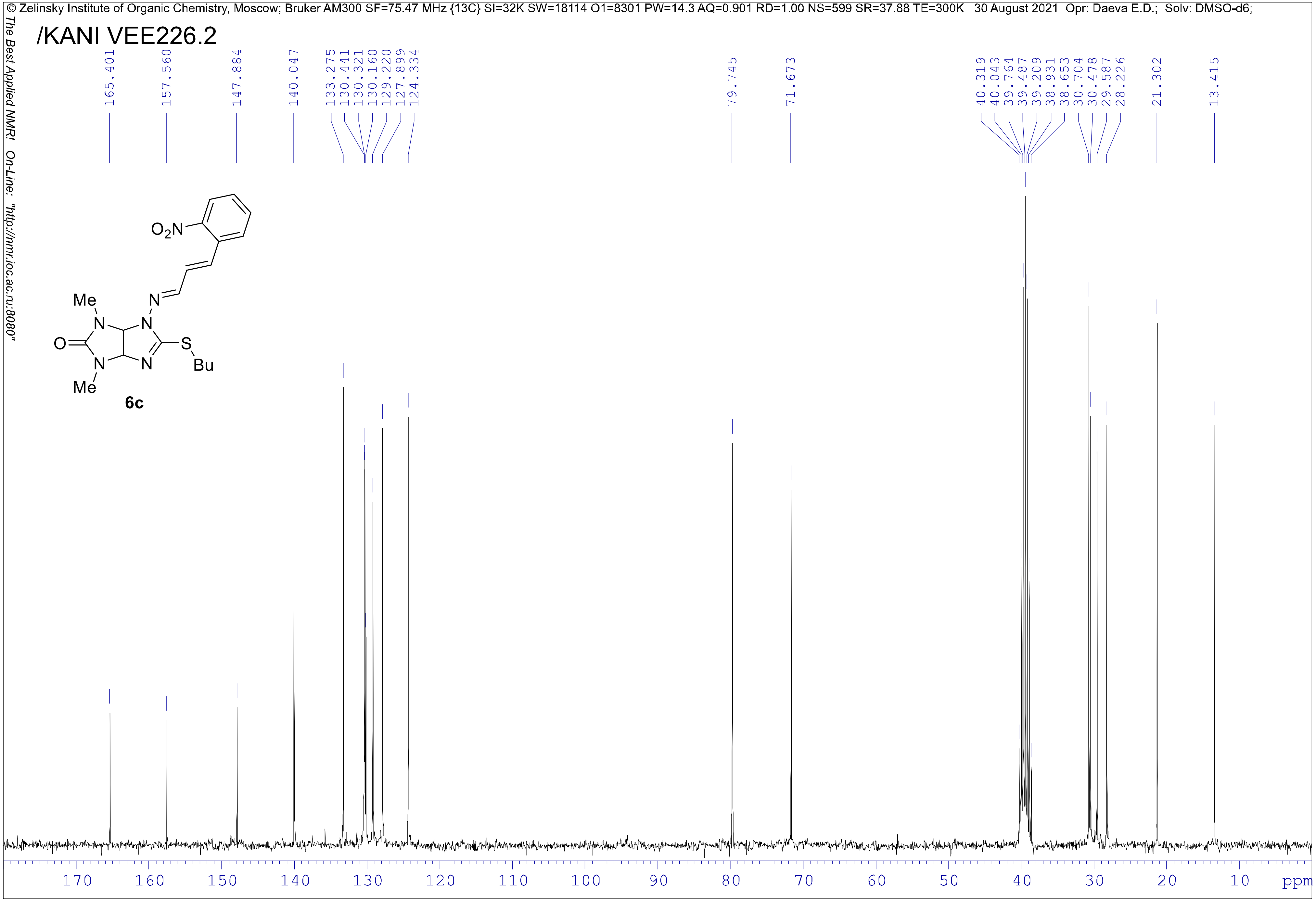
© Zelinsky Institute of Organic Chemistry, Moscow; Bruker AM300 SF=75.47 MHz {13C} SI=32K SW=18114 O1=8301 PW=14.3 AQ=0.901 RD=1.00 NS=599 SR=37.88 TE=300K 30 August 2021 Opr: Daeva E.D.; Solv: DMSO-d6;
/KANI VEE226.2
The Best Applied NMR! On-Line: "http://nmr.ioc.ac.ru:8080"
165.401
157.560
147.884
140.047
133.275
130.441
130.321
130.160
129.220
127.899
124.334
79.745
71.673
40.319
40.043
39.764
39.487
39.209
38.931
38.653
30.704
30.478
29.587
28.226
21.302
13.415
O2N
N
Me
N
N
O
N
N
S
Bu
Me
6c
170 160 150 140 130 120 110 100 90 80 70 60 50 40 30 20 10 ppm

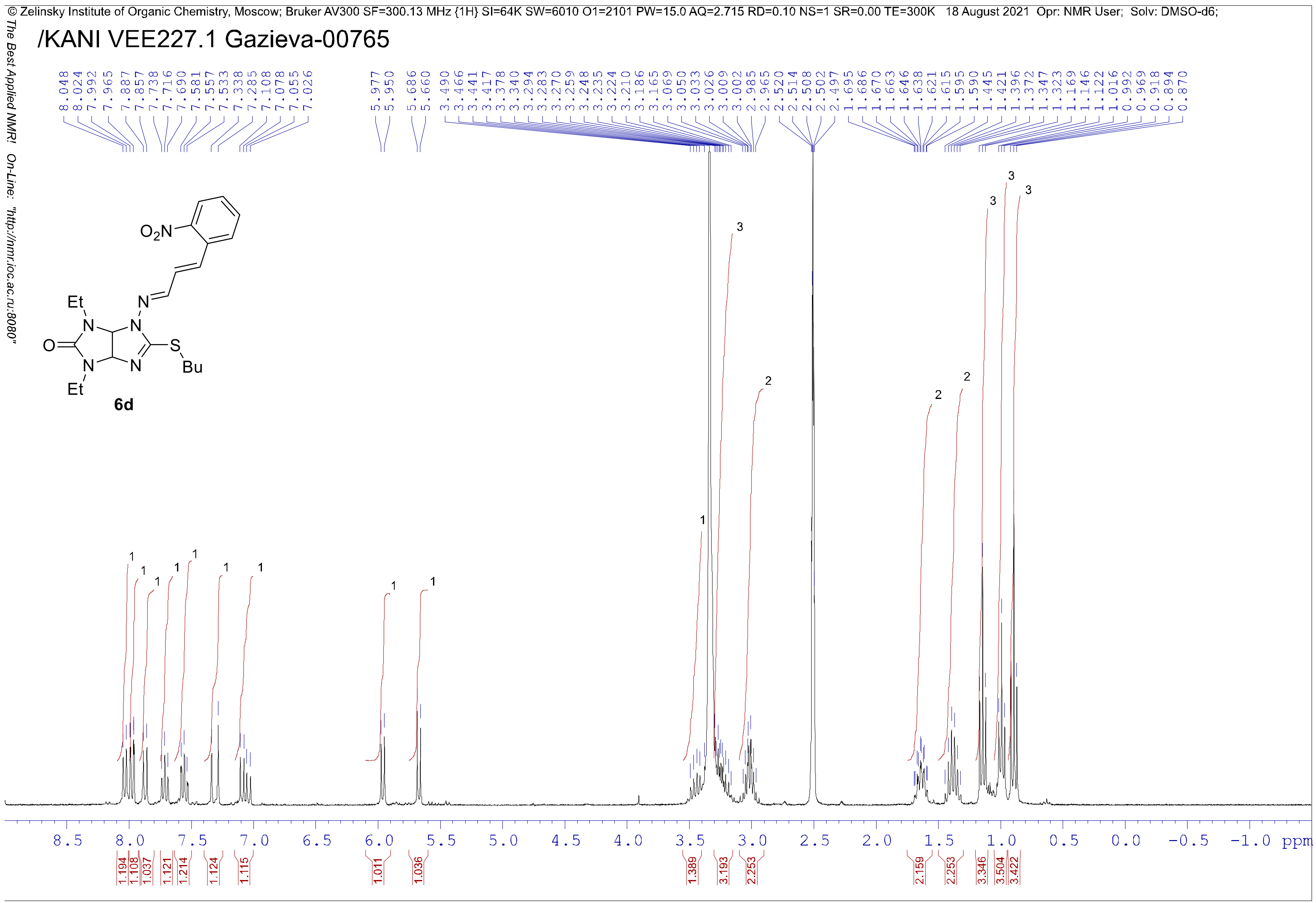
© Zelinsky Institute of Organic Chemistry, Moscow; Bruker AV300 SF=300.13 MHz {1H} SI=64K SW=6010 O1=2101 PW=15.0 AQ=2.715 RD=0.10 NS=1 SR=0.00 TE=300K 18 August 2021 Opr: NMR User; Solv: DMSO-d6;
/KANI VEE227.1 Gazieva-00765
The Best Applied NMR! On-Line: "http://nmr.ioc.ac.ru:8080"
8.048 8.024 7.992 7.965 7.887 7.857 7.738 7.716 7.690 7.581 7.557 7.533 7.338 7.285 7.108 7.078 7.055 7.026
5.977 5.950 5.686 5.660
3.490 3.466 3.441 3.417 3.378 3.340 3.294 3.283 3.270 3.259 3.248 3.235 3.224 3.210 3.186 3.165 3.069 3.050 3.033 3.026 3.009 3.002 2.985 2.965
2.520 2.514 2.508 2.502 2.497
1.695 1.686 1.670 1.663 1.646 1.638 1.621 1.615 1.595 1.590 1.445 1.421 1.396 1.372 1.347 1.323 1.169 1.146 1.122 1.016 0.992 0.969 0.918 0.894 0.870
O_2N
Et
N
O
S
Bu
6d
1.194 1.108 1.037 1.121 1.214 1.124 1.115 1.011 1.036 1.389 3.193 2.253 2.159 2.253 3.346 3.504 3.422
8.5 8.0 7.5 7.0 6.5 6.0 5.5 5.0 4.5 4.0 3.5 3.0 2.5 2.0 1.5 1.0 0.5 0.0 -0.5 -1.0 ppm

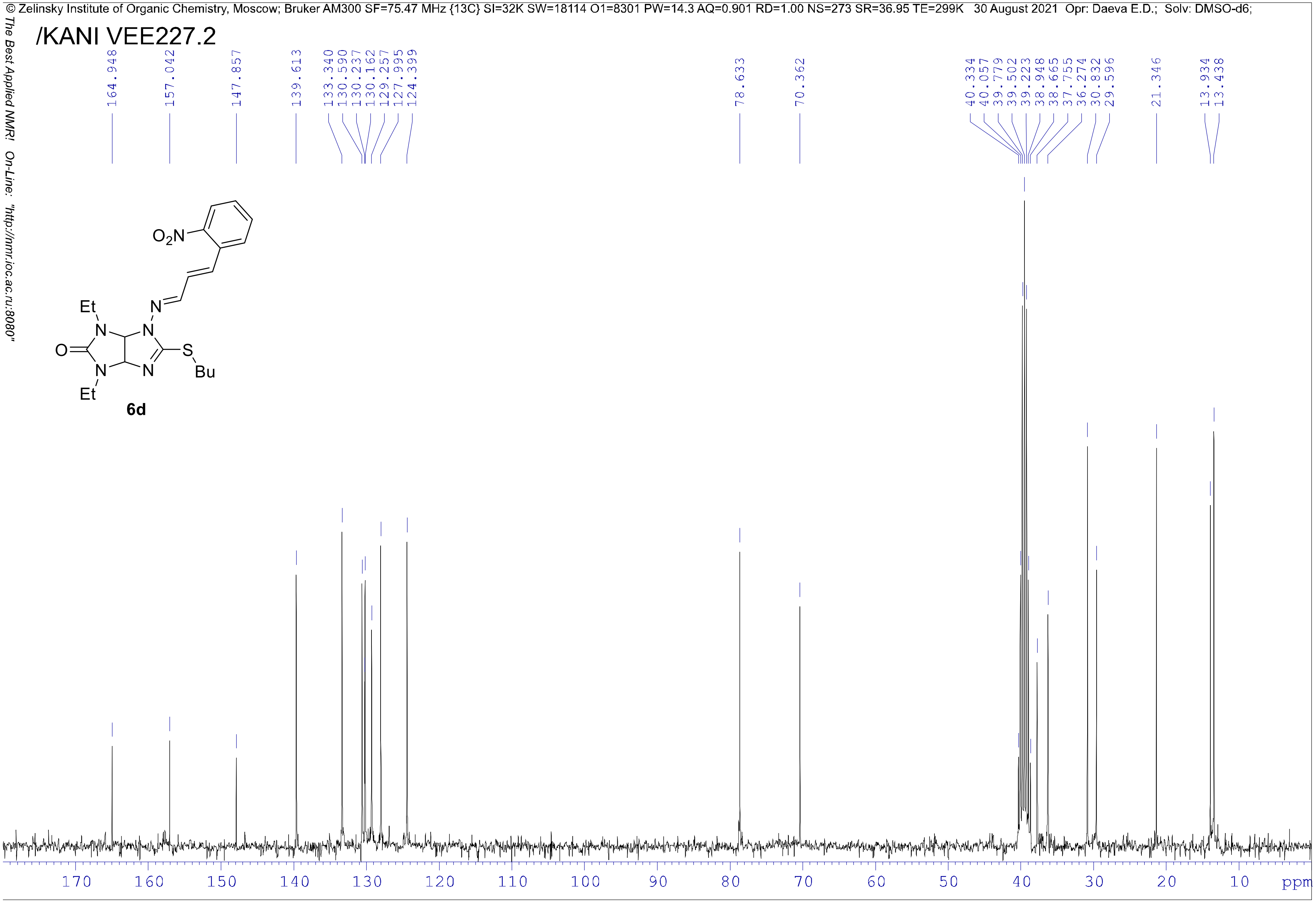
© Zelinsky Institute of Organic Chemistry, Moscow; Bruker AM300 SF=75.47 MHz {13C} SI=32K SW=18114 O1=8301 PW=14.3 AQ=0.901 RD=1.00 NS=273 SR=36.95 TE=299K 30 August 2021 Opr: Daeva E.D.; Solv: DMSO-d6;
The Best Applied NMR! On-Line: "http://nmr.ioc.ac.ru:8080"
/KANI VEE227.2
164.948
157.042
147.857
139.613
133.340
130.590
130.237
130.162
129.257
127.995
124.399
78.633
70.362
40.334
40.057
39.779
39.502
39.223
38.948
38.665
37.755
36.274
30.832
29.596
21.346
13.934
13.438
O_2N
N
Et
N
N
O
S
N
N
Bu
Et
6d
170 160 150 140 130 120 110 100 90 80 70 60 50 40 30 20 10 ppm

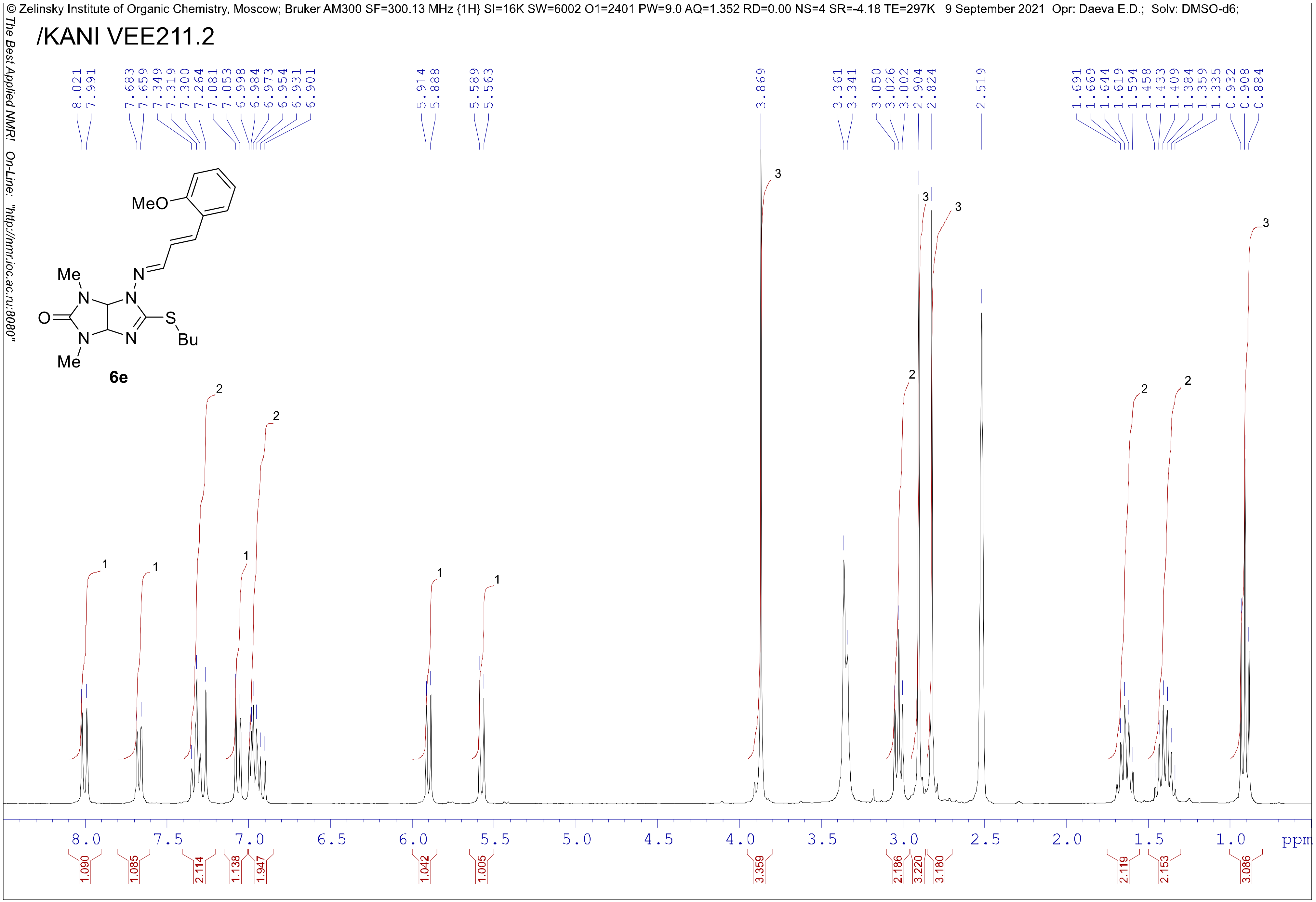

© Zelinsky Institute of Organic Chemistry, Moscow; Bruker AM300 SF=300.13 MHz {1H} SI=16K SW=6002 O1=2401 PW=9.0 AQ=1.352 RD=0.00 NS=4 SR=-4.18 TE=297K 9 September 2021 Opr: Daeva E.D.; Solv: DMSO-d6;
/KANI VEE211.2
The Best Applied NMR! On-Line: "http://nmr.ioc.ac.ru:8080"
8.021 7.991 7.683 7.659 7.349 7.319 7.300 7.264 7.081 7.053 6.998 6.984 6.973 6.954 6.931 6.901 5.914 5.888 5.589 5.563 3.869 3.361 3.341 3.050 3.026 3.002 2.904 2.824 2.519 1.691 1.669 1.644 1.619 1.594 1.458 1.433 1.409 1.384 1.359 1.335 0.932 0.908 0.884
MeO
Me
N
O
S
Bu
6e
1.090 1.085 2.114 1.138 1.947 1.042 1.005 3.359 2.186 3.220 3.180 2.119 2.153 3.086
8.0 7.5 7.0 6.5 6.0 5.5 5.0 4.5 4.0 3.5 3.0 2.5 2.0 1.5 1.0 ppm

© Zelinsky Institute of Organic Chemistry, Moscow; Bruker AM300 SF=75.47 MHz {13C} SI=32K SW=18114 O1=8301 PW=14.3 AQ=0.901 RD=1.00 NS=132 SR=35.33 TE=296K 9 September 2021 Opr: Daeva E.D.; Solv: DMSO-d6;

The Best Applied NMR! On-Line: "http://nmr.ioc.ac.ru:8080"

/KANI VEE211.2

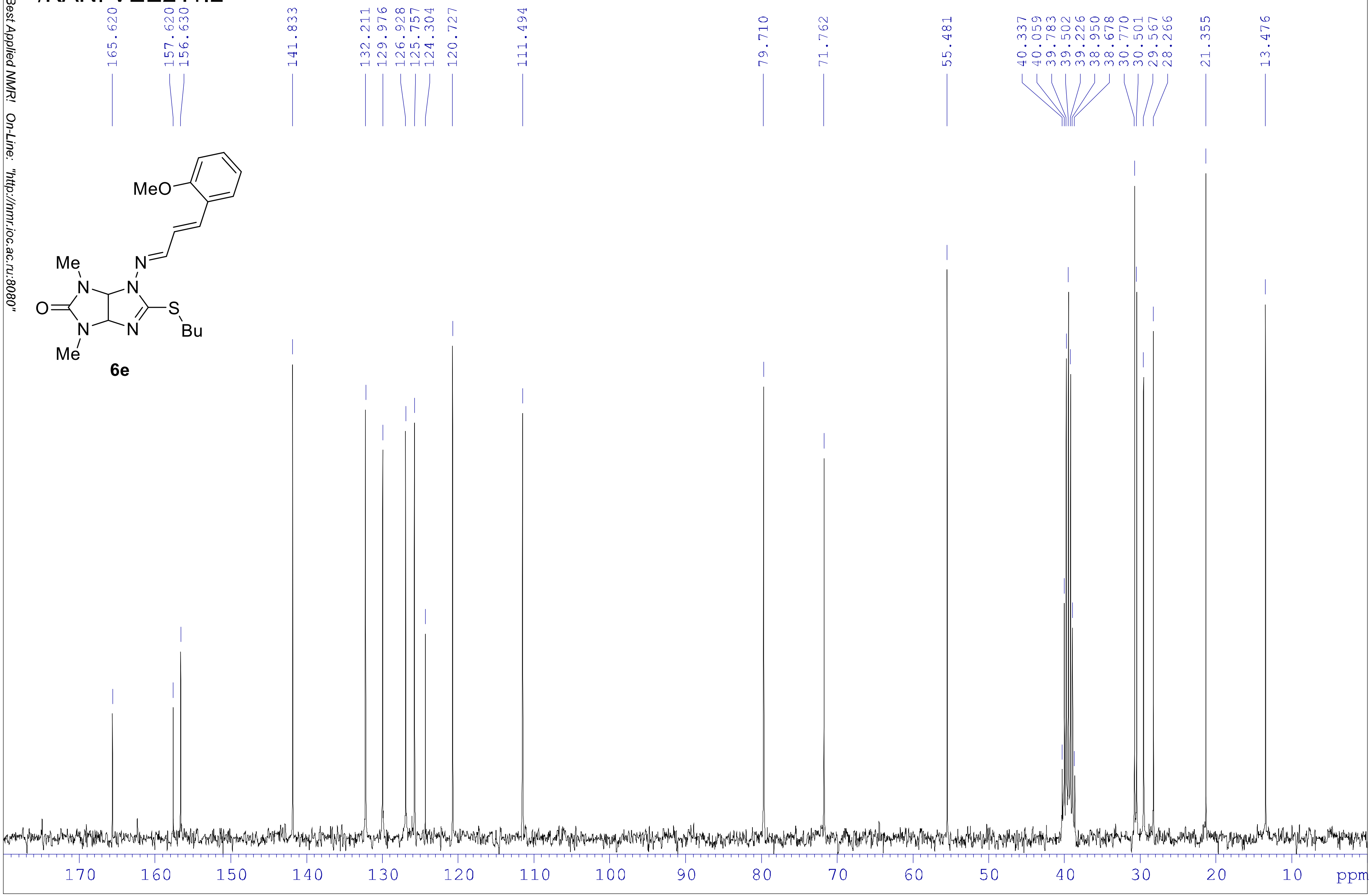

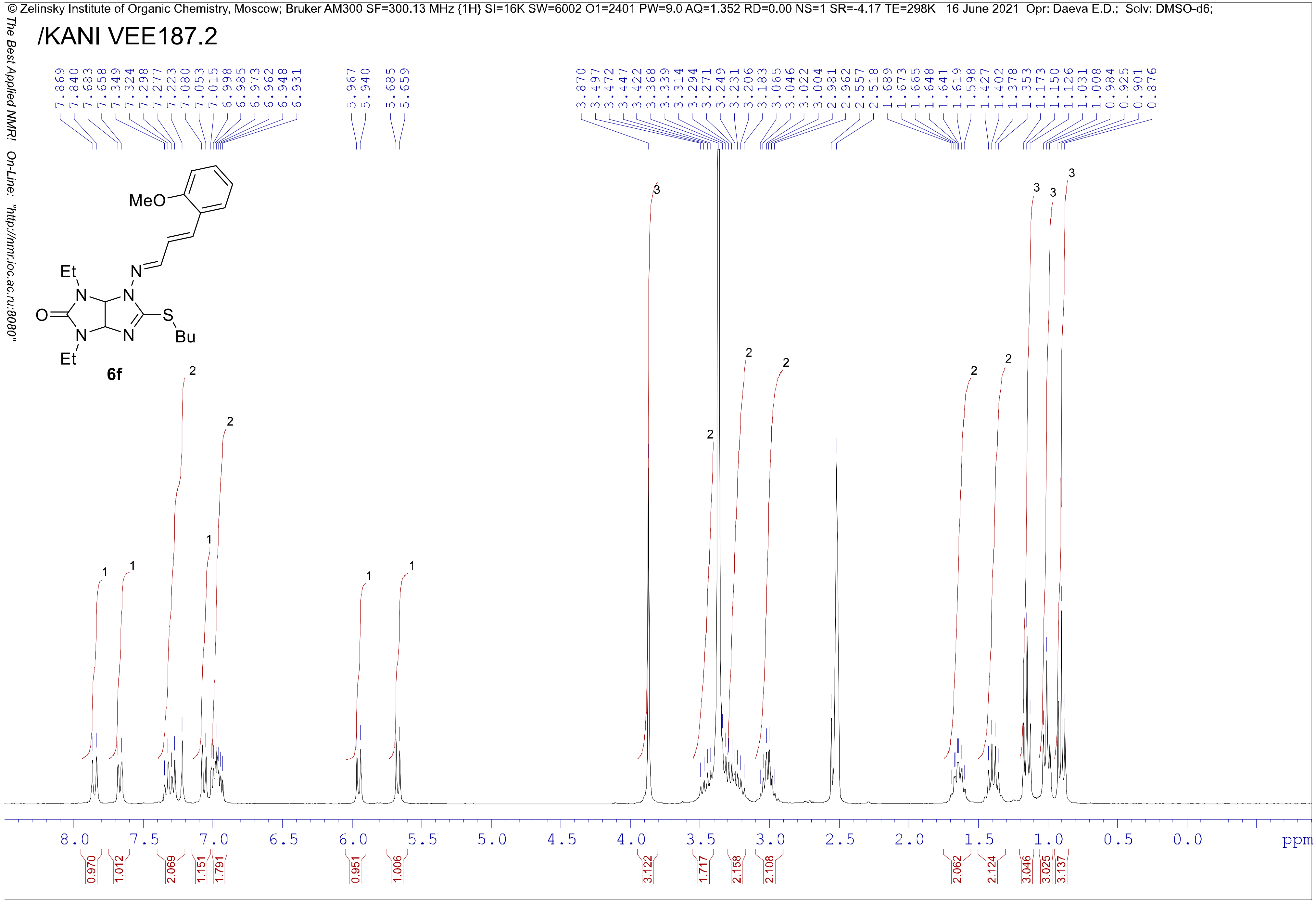
© Zelinsky Institute of Organic Chemistry, Moscow; Bruker AM300 SF=300.13 MHz {1H} SI=16K SW=6002 O1=2401 PW=9.0 AQ=1.352 RD=0.00 NS=1 SR=-4.17 TE=298K 16 June 2021 Opr: Daeva E.D.; Solv: DMSO-d6;
/KANI VEE187.2
The Best Applied NMR! On-Line: "http://nmr.ioc.ac.ru:8080"
6f
ppm

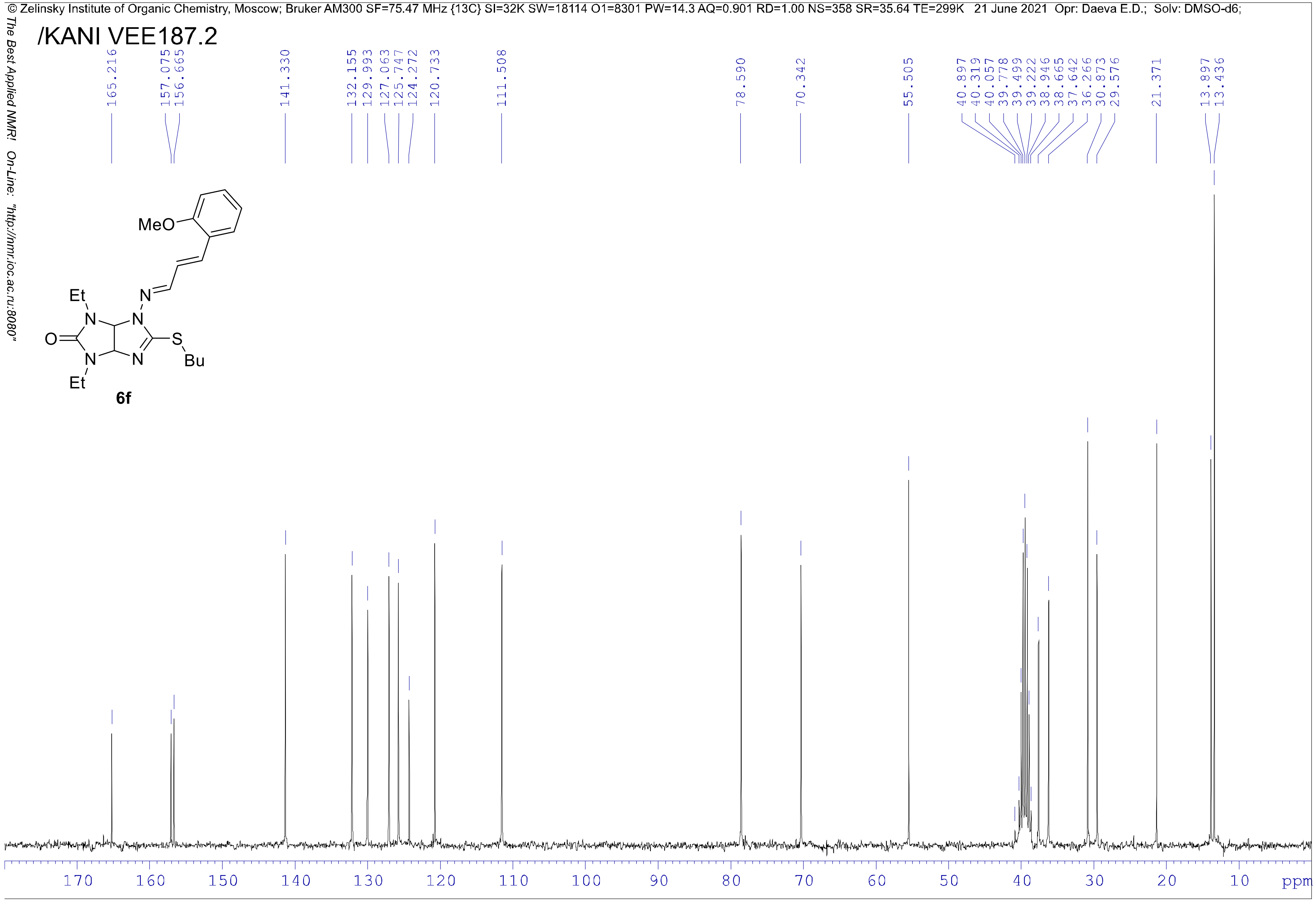
© Zelinsky Institute of Organic Chemistry, Moscow; Bruker AM300 SF=75.47 MHz {13C} SI=32K SW=18114 O1=8301 PW=14.3 AQ=0.901 RD=1.00 NS=358 SR=35.64 TE=299K 21 June 2021 Opr: Daeva E.D.; Solv: DMSO-d6;
The Best Applied NMR! On-Line: "http://nmr.ioc.ac.ru:8080"
/KANI VEE187.2
165.216
157.075
156.665
141.330
132.155
129.993
127.063
125.747
124.272
120.733
111.508
78.590
70.342
55.505
40.897
40.319
40.057
39.778
39.499
39.222
38.946
38.665
37.642
36.266
30.873
29.576
21.371
13.897
13.436
MeO
Et
N
O
S
Bu
6f
170 160 150 140 130 120 110 100 90 80 70 60 50 40 30 20 10 ppm

© Zelinsky Institute of Organic Chemistry, Moscow; Bruker AV300 SF=300.13 MHz {1H} SI=64K SW=6010 O1=2101 PW=15.0 AQ=2.715 RD=0.10 NS=1 SR=0.00 TE=300K 24 August 2021 Opr: NMR User; Solv: DMSO-d6;

/KANI VEE220.2 Gazieva-00765

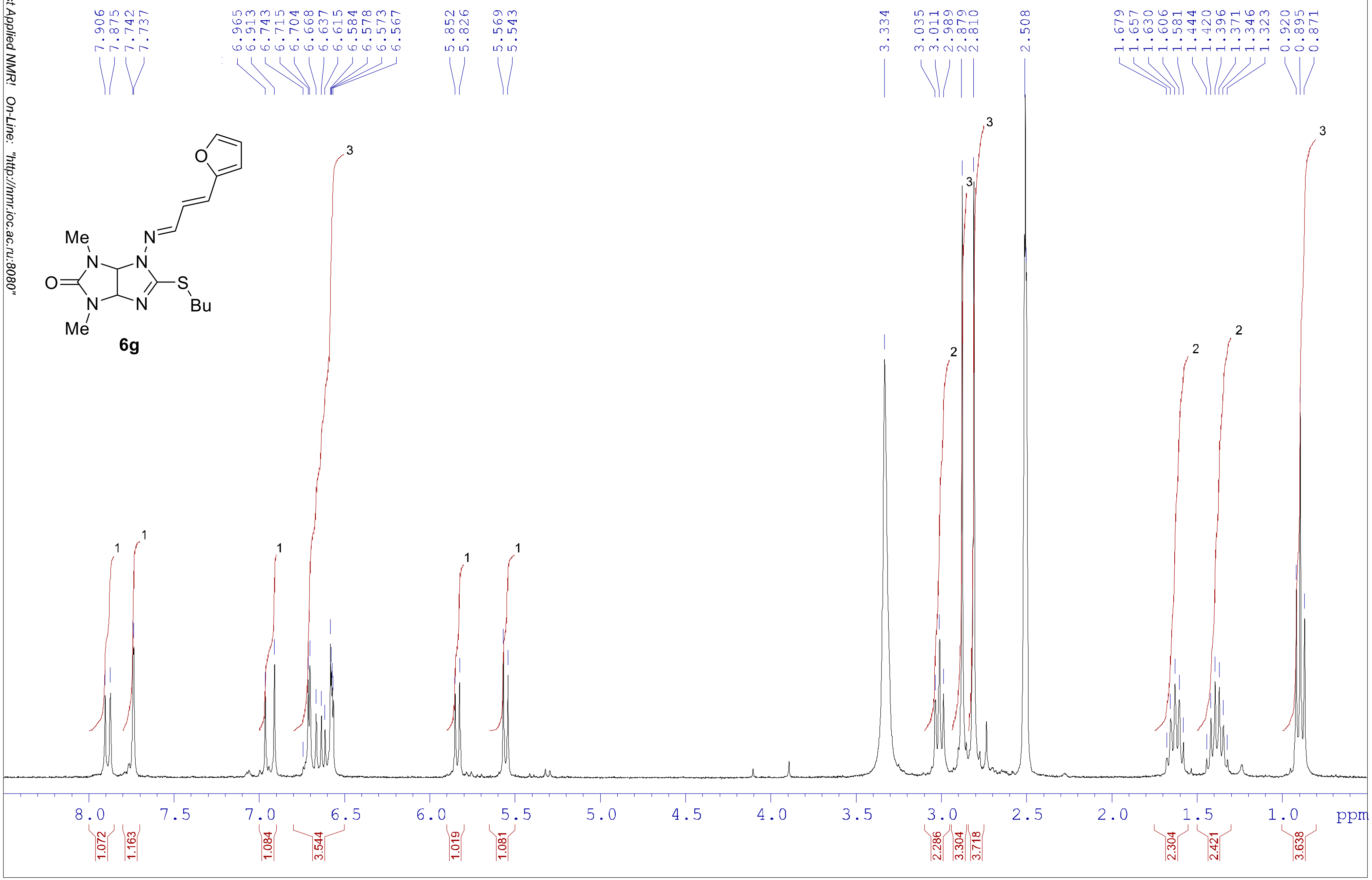

The Best Applied NMR! On-Line: "http://nmr.ioc.ac.ru:8080"

© Zelinsky Institute of Organic Chemistry, Moscow; Bruker AM300 SF=75.47 MHz {13C} SI=32K SW=18114 O1=8301 PW=14.3 AQ=0.901 RD=1.00 NS=235 SR=36.70 TE=300K 30 August 2021 Opr: Daeva E.D.; Solv: DMSO-d6;

The Best Applied NMR! On-Line: "http://nmr.ioc.ac.ru:8080"

/KANI VEE220.3

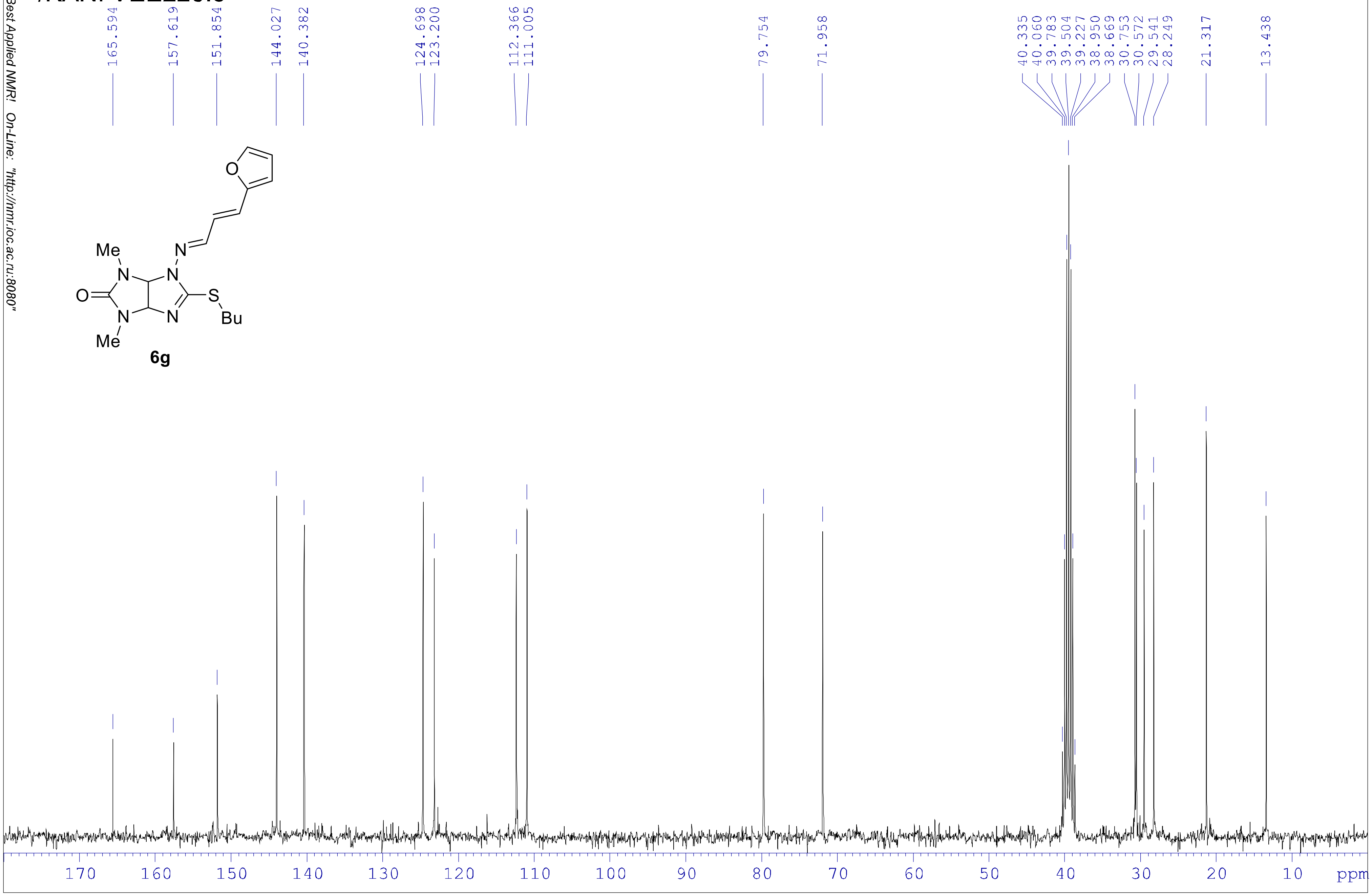

© Zelinsky Institute of Organic Chemistry, Moscow; Bruker AM300 SF=300.13 MHz {1H} SI=16K SW=6002 O1=2401 PW=9.0 AQ=1.352 RD=0.00 NS=4 SR=-4.18 TE=297K 9 September 2021 Opr: Daeva E.D.; Solv: DMSO-d6;

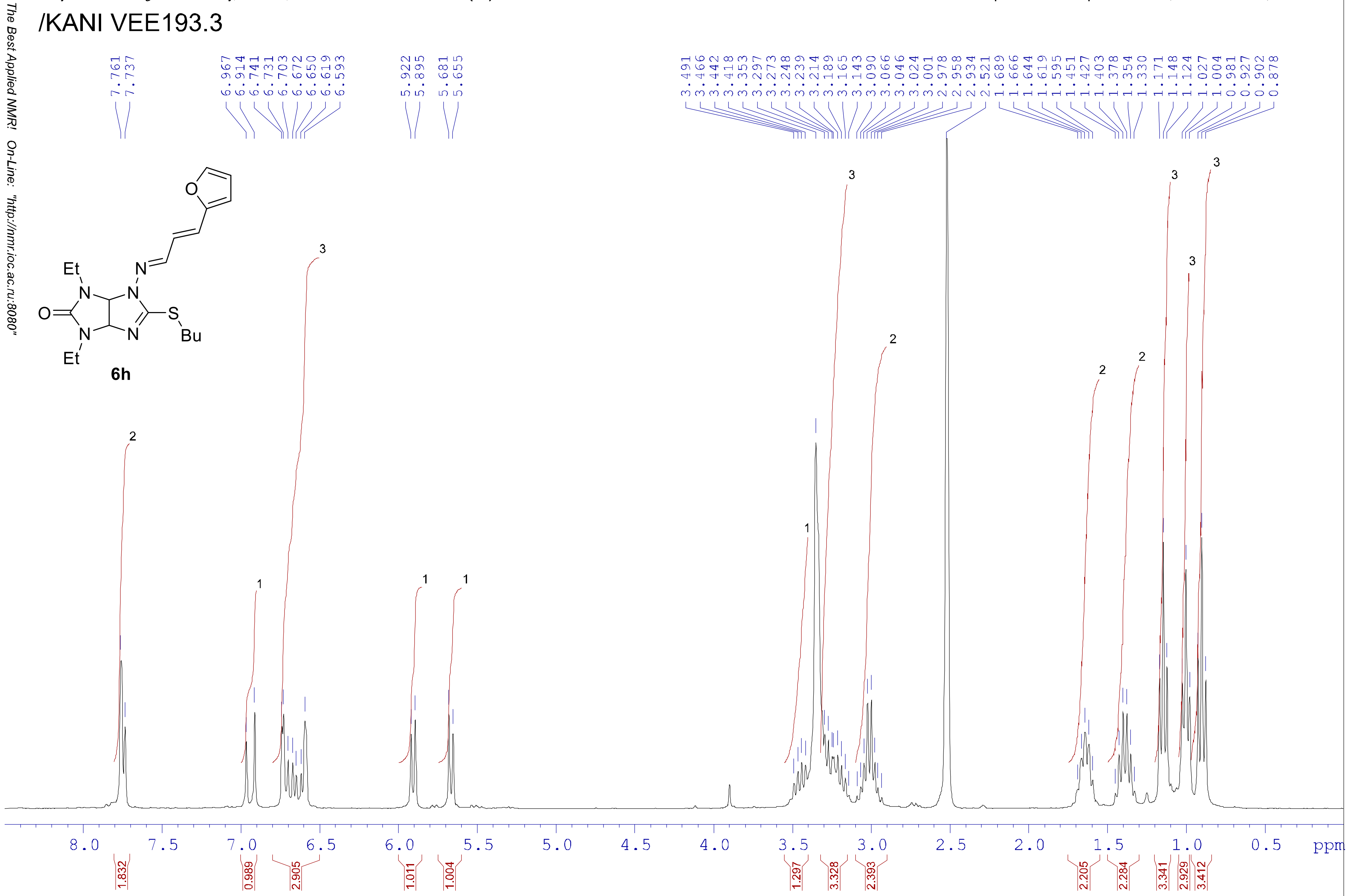

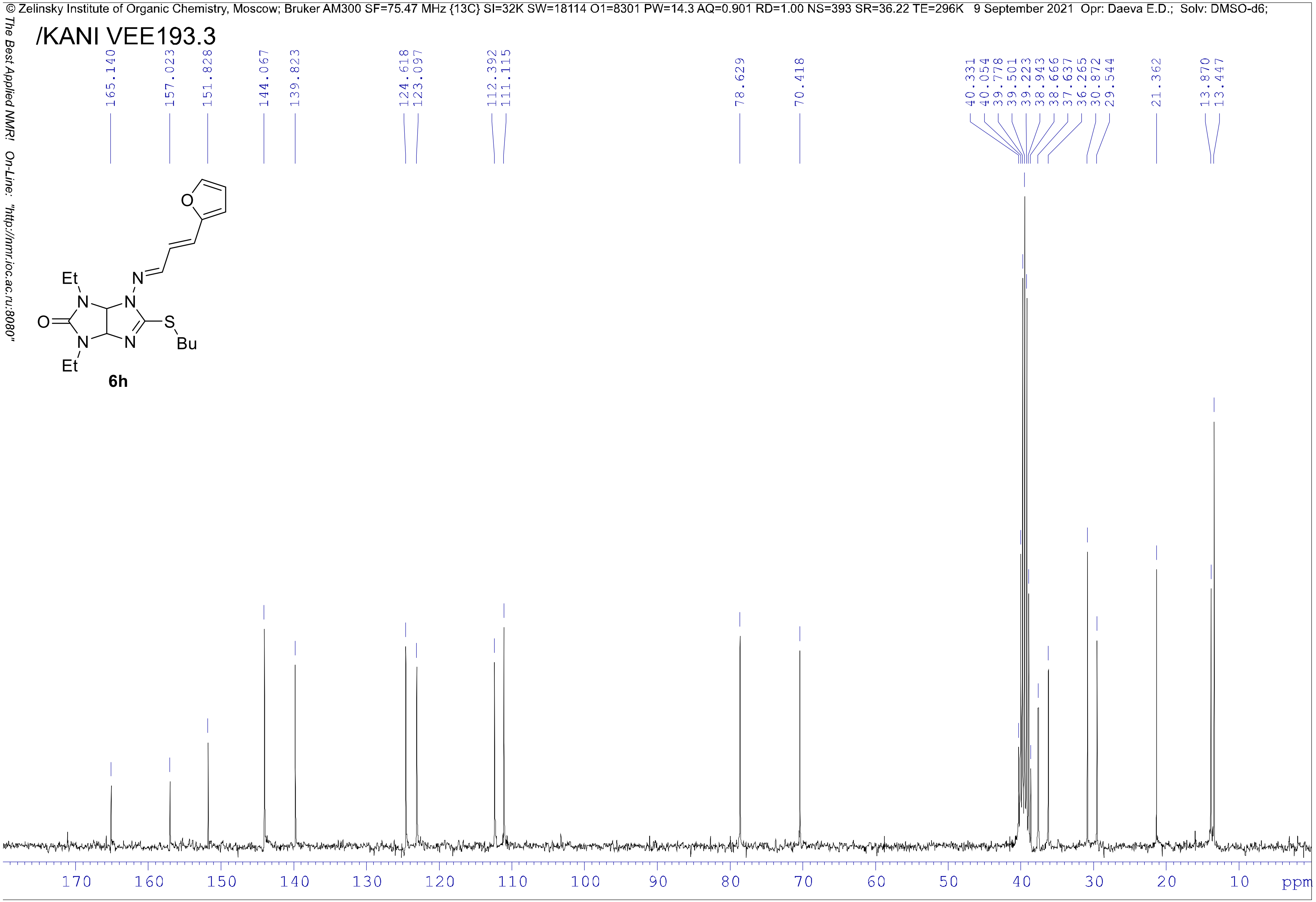

© Zelinsky Institute of Organic Chemistry, Moscow; Bruker AM300 SF=75.47 MHz {13C} SI=32K SW=18114 O1=8301 PW=14.3 AQ=0.901 RD=1.00 NS=393 SR=36.22 TE=296K 9 September 2021 Opr: Daeva E.D.; Solv: DMSO-d6;
The Best Applied NMR! On-Line: "http://nmr.ioc.ac.ru:8080"
/KANI VEE193.3
165.140
157.023
151.828
144.067
139.823
124.618
123.097
112.392
111.115
78.629
70.418
40.331
40.054
39.778
39.501
39.223
38.943
38.666
37.637
36.265
30.872
29.544
21.362
13.870
13.447
Et
N
O
Bu
S
6h
170 160 150 140 130 120 110 100 90 80 70 60 50 40 30 20 10 ppm

LC-MS data of compound 4f

```
=====================================================================
Injection Date   : 20/01/2020 11:07:53            Seq. Line :  24
Sample Name      : ZNA868F1                        Location : P1-C-11
Acq. Operator    : 4                                    Inj :   1
Acq. Instrument  : Instrument 1                  Inj Volume : 3 µl
Different Inj Volume from Sequence !      Actual Inj Volume : 1 µl
Acq. Method      : C:\HPCHEM\1\METHODS\1PH.M
Last changed     : 17/01/2020 09:50:42  by 4
Analysis Method  : C:\HPCHEM\1\METHODS\1PH'.M
Last changed     : 30/10/2019 14:02:01  by 4
Column: ONYX MONOLITHIC, C18 50x4.6mm |3.75ml/min| Columns Reg Valve
Gradient: "A"->@2.0min->"B"(Hold 0.6min)->@0.2min->"A"->PostRun
=====================================================================

Instrument Conditions     :    At Start           At Stop
Pressure                  :     141.7             92.3   bar
Flow                      :       3.750            3.750 ml/min

Detector Lamp Burn Times: Current On-Time  Accumulated On-Time
DAD 1, UV Lamp            :       1.79          73335.0   h
DAD 1, Visible Lamp       :      OFF            1040.3   h

Solvent Description       :
PMP1, Solvent A           : 0.1%TFA, 2.5%AcN in H2O
PMP1, Solvent B           : 0.1%TFA in AcN
PMP1, Solvent C           : --NOT USED--
PMP1, Solvent D           : --NOT USED--
=====================================================================
MSD parameters
Tune file name                :    C:\HPCHEM\1\1956ATUN\atunes.tun
Ionization mode               :    API-ES

MSD Instrument Conditions     :      At Start         At Stop
Quad Temp                     :            99              99 C
Gas Temp                      :           350             350 C
RoughVac                      :             2               2 Torr
HighVac                       :      6.4E-006        6.4E-006 Torr
CapCur                        :            39             588 nA
ChamCur                       :      9.8E-001        2.0E-001 µA
DryingGas                     :            11              11 l/min
Neb Pres                      :            50              50 psig
Turbo1Spd                     :           100             101 %
Turbo1Pwr                     :           105             108 W
RF Drive                      :      0.0E-001        0.0E-001 %
Qd TpDrv                      :             8               8 %
Gas TpDrv                     :            35              35 %
Neb PrDrv                     :            48              48 %
Gas FlDrv                     :            63              64 %
DelaySens                     :     -9.9E-002        0.0E-001 V
Aux Input                     :      0.0E-001        0.0E-001 V
Other Det                     :      0.0E-001        0.0E-001 V
=====================================================================
MSD tuning (calibration) parameters

Ionization polarity           :    Positive
Skim1                         :    Not Applicable
Skim2                         :    8.0 V
Ion Energy                    :    5.0 V
Lens1                         :    1.7 V
Lens2                         :    17 V
Iris                          :    -200 V
HED                           :    10000 V
Width Gain                    :    -627
Width Offset                  :    Variable
                                   Mass          :   Value
                                   --------------------------
                                   118.08        :   -58
                                   622.03        :   -58
                                   922.01        :   -58
                                   --------------------------
Mass Gain                     :    -74.95
Mass Offset                   :    0.608
Quad DC                       :    0.00 V
Octopole Peak                 :    650 V
Octopole Knee                 :    Not Applicable
```

```
Lens2DC                          :    Not Applicable
L2RFEn                           :    Not Applicable
L2RFPh                           :    Not Applicable
L2RFAmp                          :    Not Applicable
Mass Filter                      :    Gaussian
Mass Filter Width                :    0.30 Da
Time Filter                      :    Gaussian
Time Filter Width                :    0.030 minutes
```

===

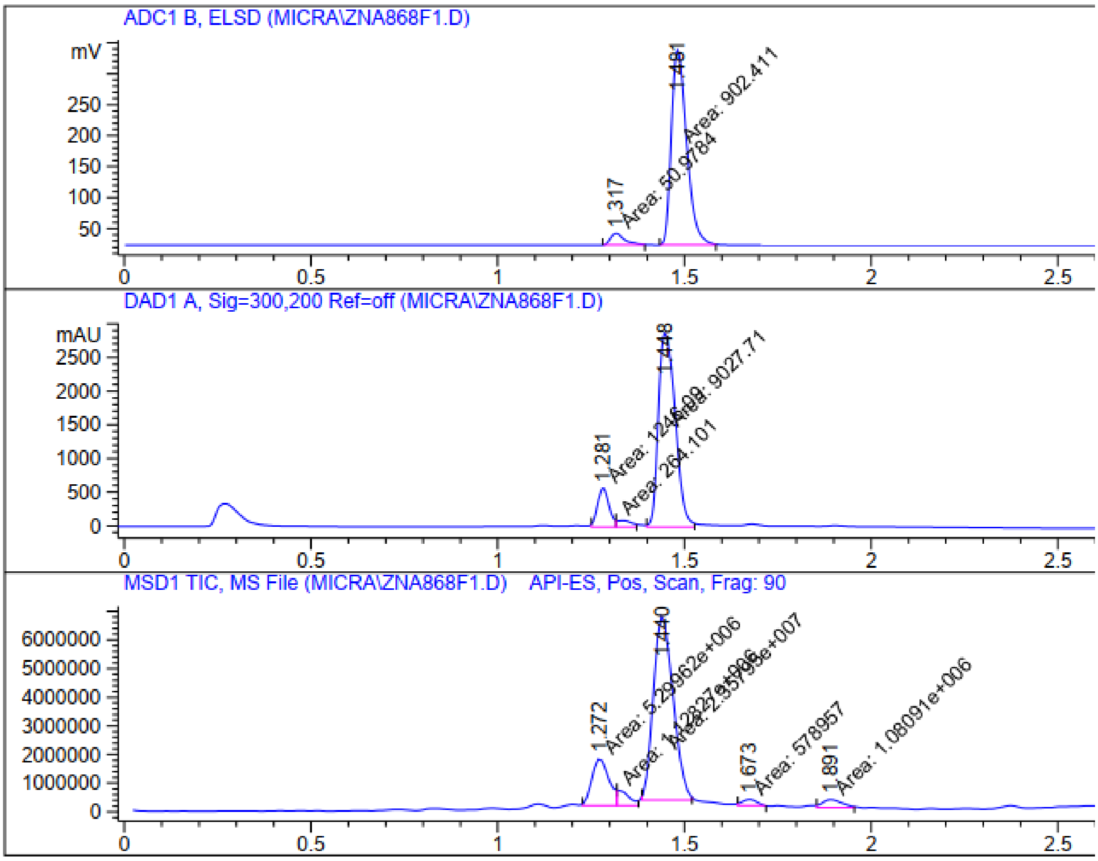

===

Area Percent Report

===

```
Sorted By                :      Signal
Multiplier               :      1.0000
Dilution                 :      1.0000
Use Multiplier & Dilution Factor with ISTDs
```

Signal 1: ADC1 B, ELSD

Peak #	RetTime [min]	Type	Width [min]	Area [mV*s]	Height [mV]	Area %
1	1.317	MM	0.0446	50.97838	19.05589	5.3471
2	1.481	MM	0.0477	902.41071	315.13181	94.6529
Totals :				953.38909	334.18770	

Signal 2: DAD1 A, Sig=300,200 Ref=off

Peak #	RetTime [min]	Type	Width [min]	Area [mAU*s]	Height [mAU]	Area %
1	1.281	MM	0.0356	1246.09180	583.36029	11.8249
2	1.316	MM	0.0390	264.10107	99.33130	2.5062
3	1.448	MM	0.0518	9027.70703	2906.34717	85.6689
Totals :				1.05379e4	3589.03876	

Signal 3: MSD1 TIC, MS File

Peak #	RetTime [min]	Type	Width [min]	Area	Height	Area %
1	1.272	MM	0.0531	5.29962e6	1.66350e6	16.7353
2	1.318	MM	0.0348	1.12827e6	5.40972e5	3.5629
3	1.440	MM	0.0605	2.35795e7	6.49316e6	74.4602
4	1.673	MM	0.0427	5.78957e5	2.25952e5	1.8283
5	1.891	MM	0.0596	1.08091e6	3.02501e5	3.4133

Totals : 3.16672e7 9.22609e6

==

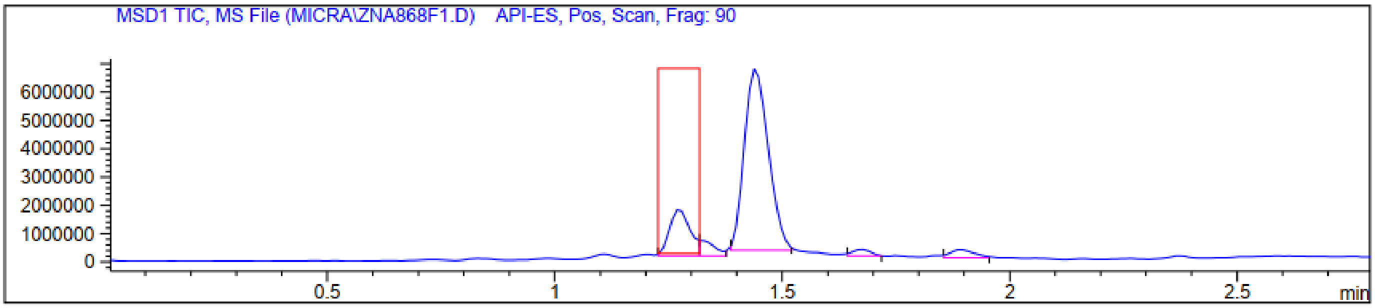

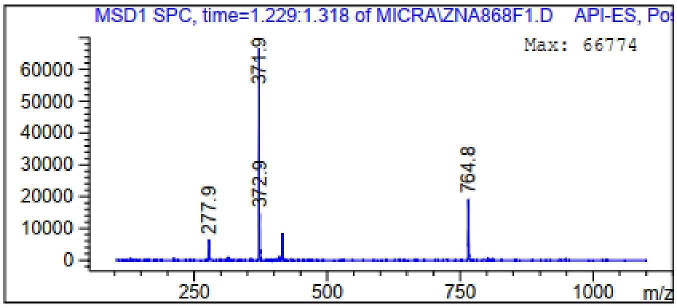

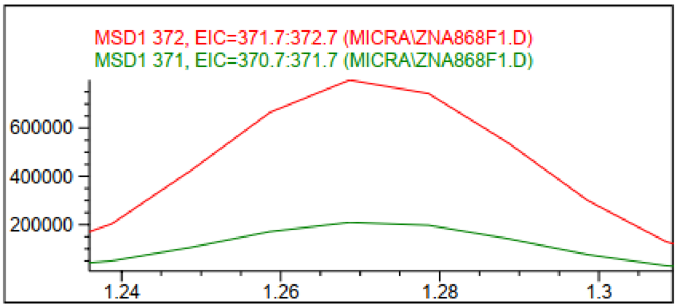


```
Peak #1 at  1.272 min ( 1.227 to  1.318 min)
-> The analysis found only one component, indicating a pure peak. <-

Component 1:  Peak at Scan 126.4. Top ions are  372 371 765
```

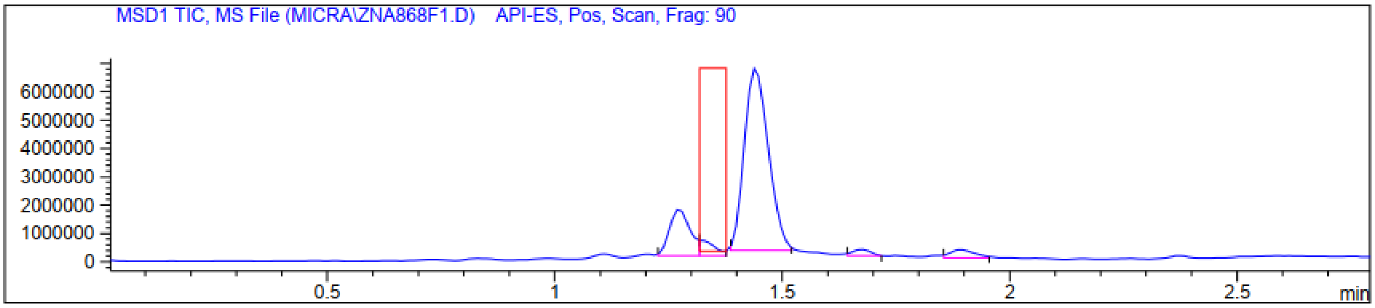

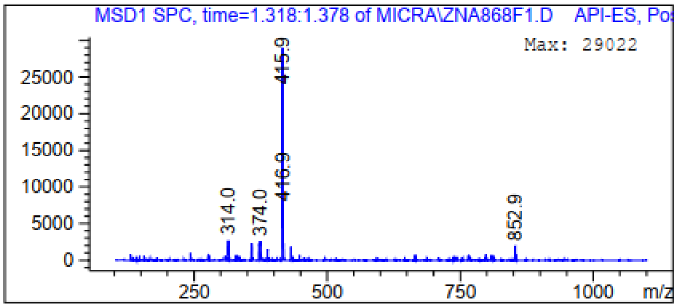

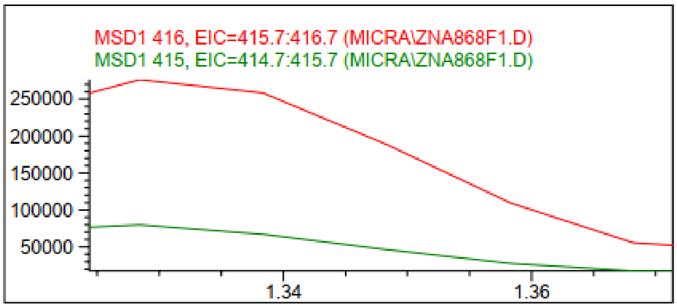


```
Peak #2 at  1.318 min ( 1.318 to  1.377 min)
-> The analysis found only one component, indicating a pure peak. <-

Component 1:  Peak at Scan 132.0. Top ions are  416 415 417
```

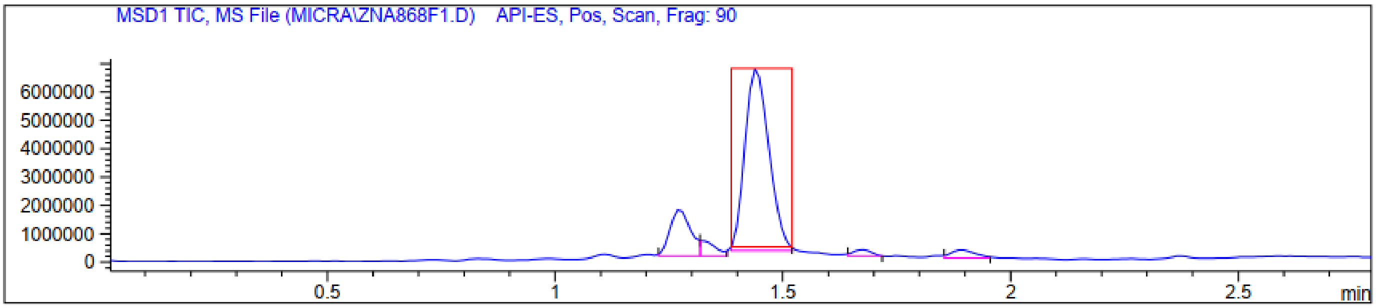

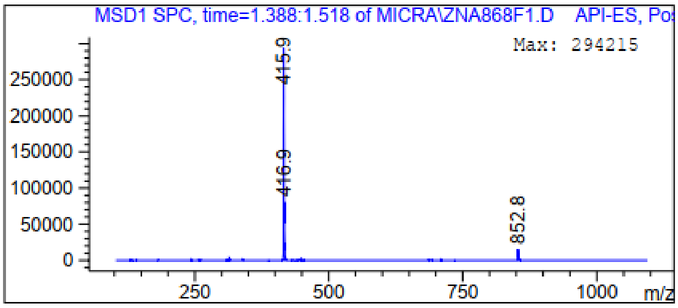

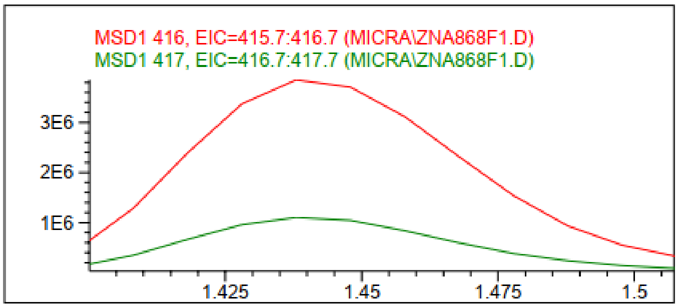

Peak #3 at 1.440 min (1.387 to 1.521 min)
-> The analysis found only one component, indicating a pure peak. <-

Component 1: Peak at Scan 143.3. Top ions are 416 417 415

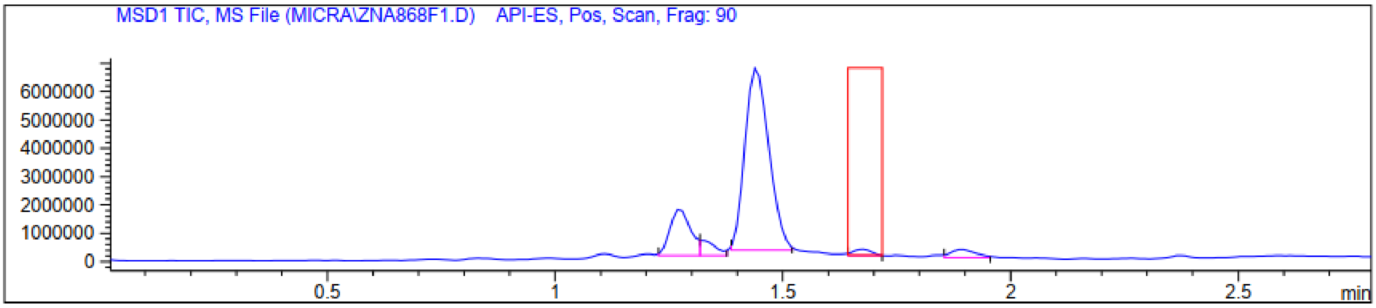

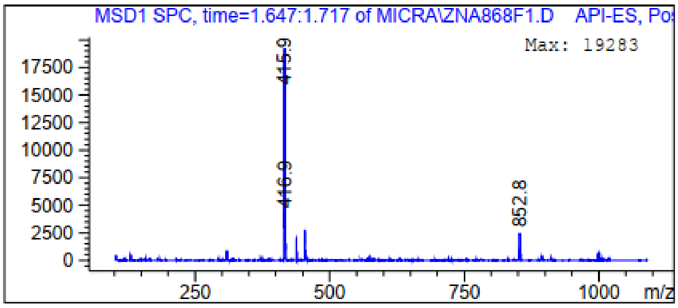

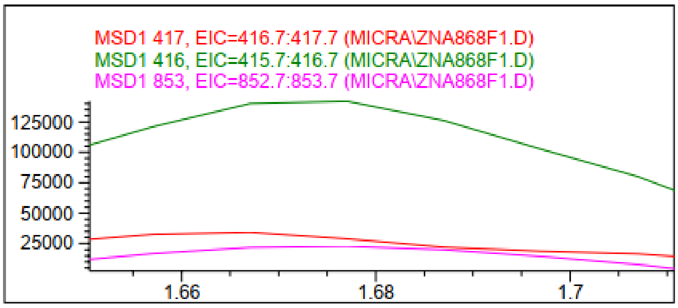

Peak #4 at 1.673 min (1.643 to 1.718 min)
-> The analysis found 2 components, indicating an impure peak. <-

Component 1: Peak at Scan 165.7. Top ions are 417
Component 2: Peak at Scan 166.7. Top ions are 416 853 454

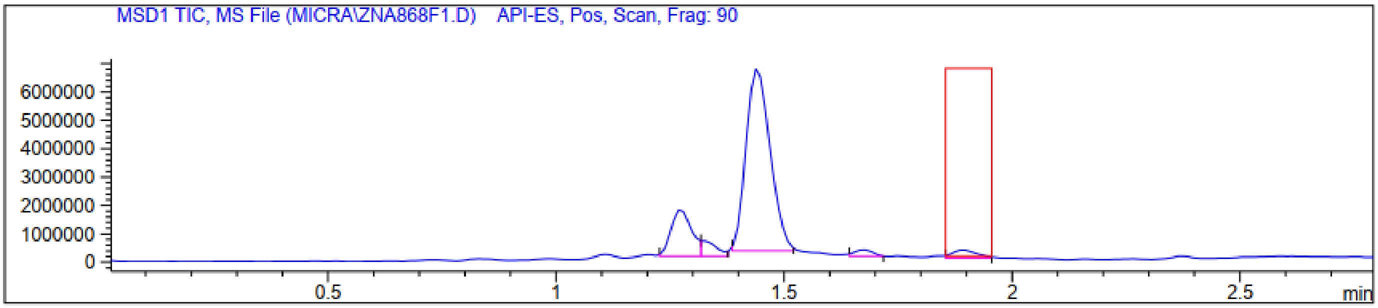

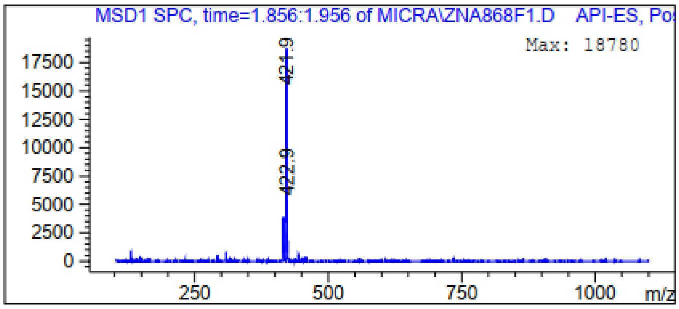

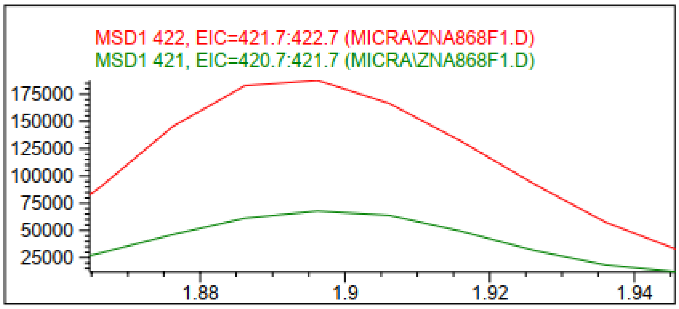


```
Peak #5 at  1.891 min ( 1.855 to  1.956 min)
-> The analysis found only one component, indicating a pure peak. <-

Component 1:  Peak at Scan 188.9. Top ions are  422 421 423
```

*** End of Report ***

Anticancer Screening Data of 60 Cancer Cell Lines of **4b**

Developmental Therapeutics Program	NSC: D-825042 / 1	Conc: 1.00E-5 Molar	Test Date: Sep 14, 2020
One Dose Mean Graph	Experiment ID: 2009OS47	4b	Report Date: Oct 06, 2020

Panel/Cell Line	Growth Percent
Leukemia	
CCRF-CEM	95.54
HL-60(TB)	73.28
K-562	83.34
MOLT-4	85.27
RPMI-8226	81.64
SR	91.80
Non-Small Cell Lung Cancer	
A549/ATCC	88.29
EKVX	96.34
HOP-62	97.93
HOP-92	100.37
NCI-H226	84.65
NCI-H23	94.97
NCI-H322M	96.78
NCI-H460	98.02
NCI-H522	84.06
Colon Cancer	
COLO 205	106.92
HCC-2998	108.45
HCT-116	86.43
HCT-15	96.93
HT29	94.79
KM12	99.10
SW-620	95.41
CNS Cancer	
SF-268	95.74
SF-295	91.78
SF-539	98.13
SNB-19	89.42
SNB-75	88.45
U251	87.16
Melanoma	
LOX IMVI	96.19
MALME-3M	96.11
M14	96.88
MDA-MB-435	95.92
SK-MEL-2	105.51
SK-MEL-28	108.47
SK-MEL-5	96.07
UACC-257	103.56
UACC-62	74.33
Ovarian Cancer	
IGROV1	97.65
OVCAR-3	100.29
OVCAR-4	88.55
OVCAR-5	101.72
OVCAR-8	97.77
NCI/ADR-RES	99.94
SK-OV-3	97.91
Renal Cancer	
786-0	105.18
A498	89.03
ACHN	102.10
CAKI-1	100.50
RXF 393	89.00
SN12C	97.89
TK-10	117.64
UO-31	70.05
Prostate Cancer	
PC-3	72.32
DU-145	99.55
Breast Cancer	
MCF7	96.62
MDA-MB-231/ATCC	92.31
HS 578T	103.00
BT-549	108.57
T-47D	83.32
MDA-MB-468	91.27
Mean	94.44
Delta	24.39
Range	47.59

Mean Growth Percent - Growth Percent

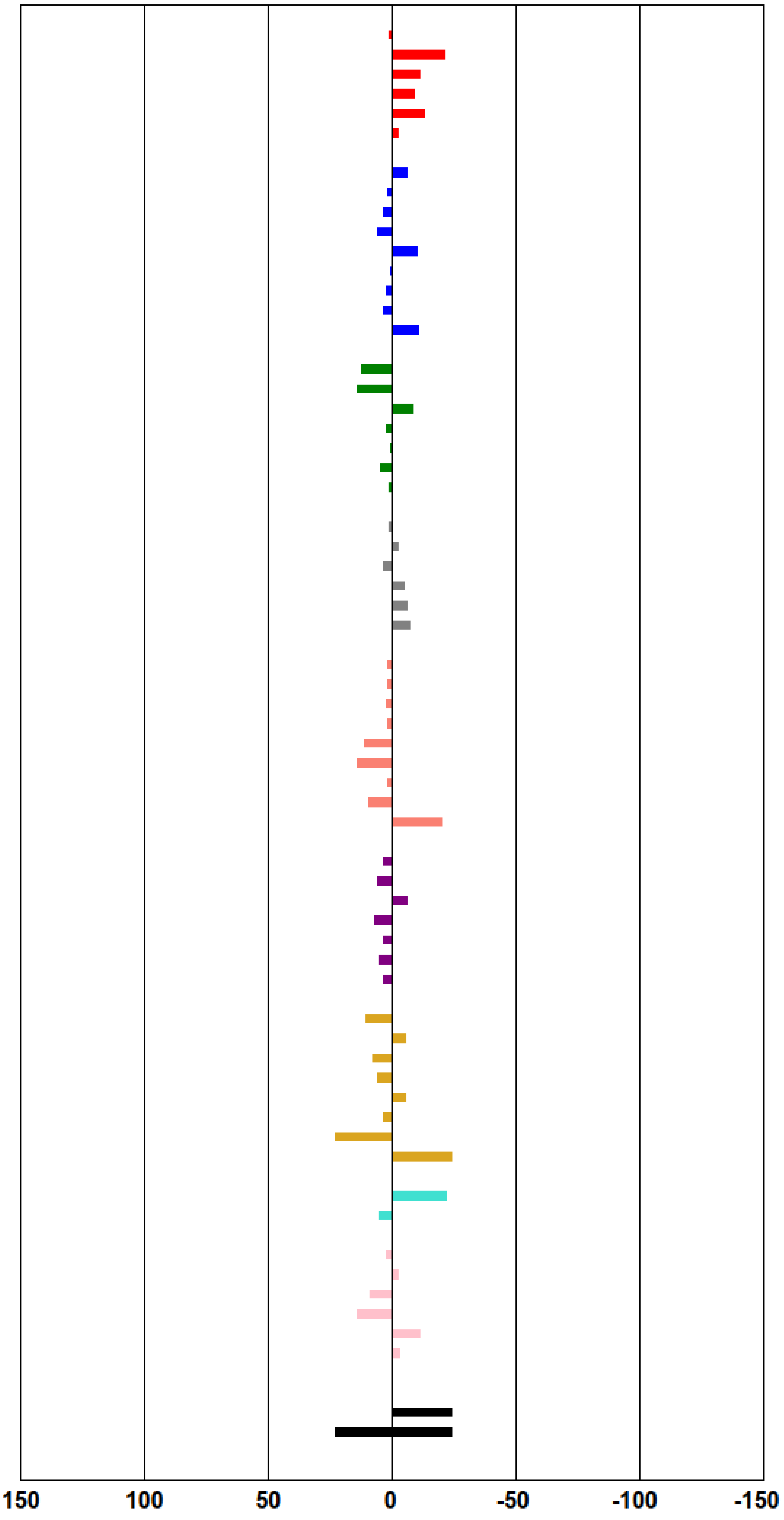

Anticancer Screening Data of 60 Cancer Cell Lines of **5d**

Developmental Therapeutics Program One Dose Mean Graph	NSC: D-829482 / 1	Conc: 1.00E-5 Molar	Test Date: Mar 29, 2021
	Experiment ID: 5d		Report Date: Apr 30, 2021

Panel/Cell Line	Growth Percent
**Leukemia**	
CCRF-CEM	92.13
HL-60(TB)	86.15
K-562	91.81
MOLT-4	90.28
RPMI-8226	85.95
SR	77.50
**Non-Small Cell Lung Cancer**	
A549/ATCC	105.99
EKVX	92.57
HOP-62	98.90
HOP-92	89.44
NCI-H226	94.69
NCI-H23	96.70
NCI-H322M	95.23
NCI-H460	106.90
NCI-H522	76.42
**Colon Cancer**	
COLO 205	114.74
HCC-2998	109.43
HCT-116	94.23
HCT-15	99.90
HT29	91.84
KM12	97.83
SW-620	104.67
**CNS Cancer**	
SF-268	95.33
SF-295	97.69
SF-539	90.24
SNB-19	94.52
SNB-75	99.49
U251	94.54
**Melanoma**	
LOX IMVI	112.33
MALME-3M	99.75
MDA-MB-435	102.48
SK-MEL-2	96.60
SK-MEL-28	101.72
SK-MEL-5	89.12
UACC-257	105.57
UACC-62	79.52
**Ovarian Cancer**	
IGROV1	99.95
OVCAR-3	110.43
OVCAR-4	92.97
OVCAR-5	91.83
OVCAR-8	96.86
NCI/ADR-RES	90.98
SK-OV-3	112.24
**Renal Cancer**	
786-0	98.20
A498	73.83
ACHN	97.79
SN12C	91.10
TK-10	116.54
UO-31	80.90
**Prostate Cancer**	
PC-3	93.33
DU-145	101.99
**Breast Cancer**	
MCF7	89.61
MDA-MB-231/ATCC	93.35
HS 578T	106.40
BT-549	105.69
T-47D	68.34
MDA-MB-468	90.06
Mean	95.69
Delta	27.35
Range	48.20

Mean Growth Percent - Growth Percent

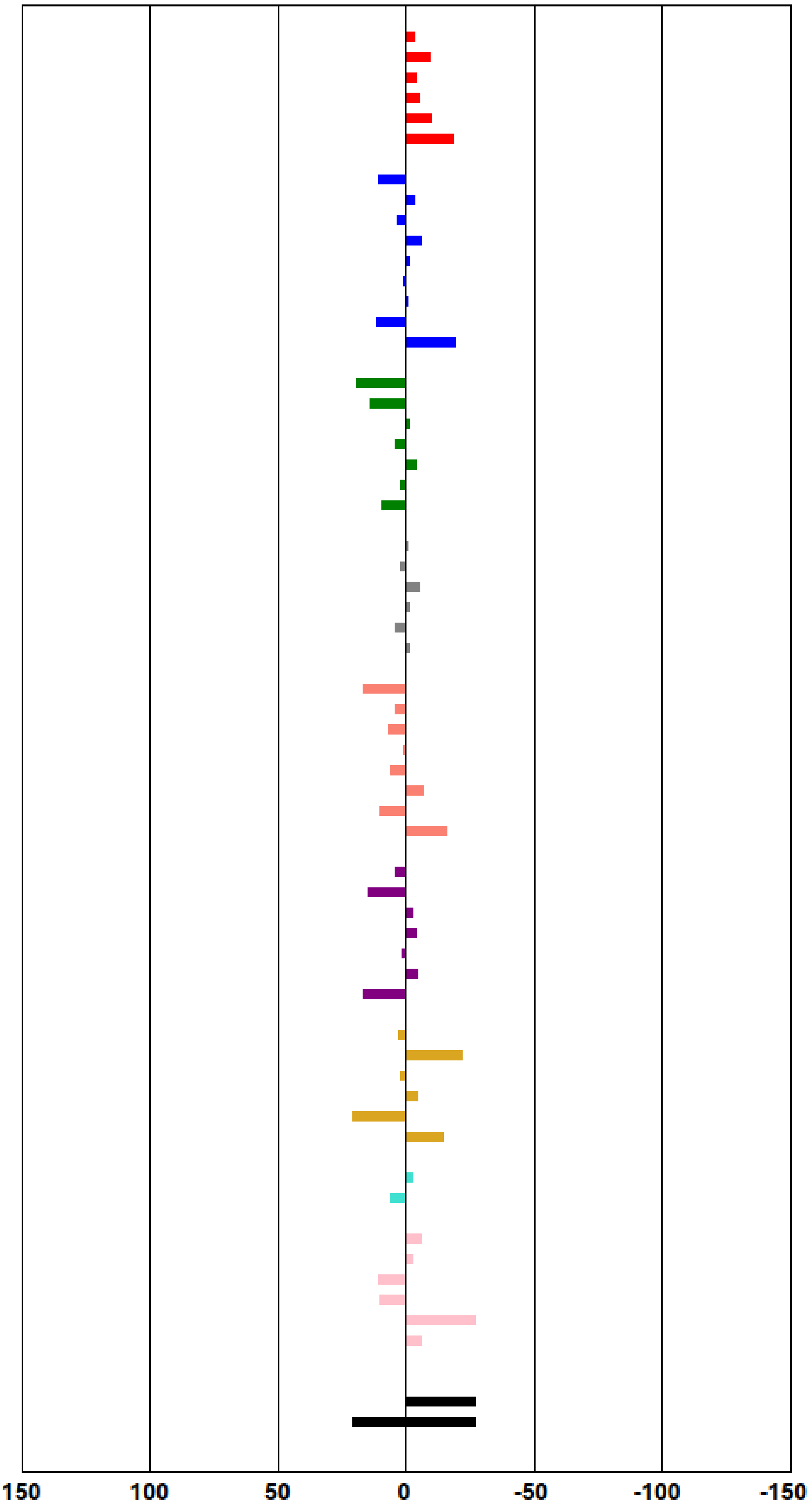

Anticancer Screening Data of 60 Cancer Cell Lines of **5e**

Developmental Therapeutics Program One Dose Mean Graph	NSC: D-828818 / 1	Conc: 1.00E-5 Molar	Test Date: Mar 22, 2021
	Experiment ID:	5e	Report Date: Apr 22, 2021

Panel/Cell Line	Growth Percent
Leukemia	
CCRF-CEM	97.68
HL-60(TB)	73.79
K-562	89.19
MOLT-4	92.40
RPMI-8226	79.23
SR	87.73
Non-Small Cell Lung Cancer	
A549/ATCC	90.84
EKVX	93.30
HOP-62	101.13
HOP-92	91.51
NCI-H226	101.83
NCI-H23	98.98
NCI-H322M	103.78
NCI-H460	98.48
NCI-H522	86.05
Colon Cancer	
COLO 205	110.33
HCC-2998	99.65
HCT-116	104.07
HCT-15	89.98
HT29	102.22
KM12	92.76
SW-620	113.50
CNS Cancer	
SF-268	94.75
SF-295	97.84
SF-539	89.36
SNB-19	90.93
SNB-75	124.64
U251	89.88
Melanoma	
LOX IMVI	89.64
MALME-3M	110.56
MDA-MB-435	91.06
SK-MEL-2	104.24
SK-MEL-28	101.46
SK-MEL-5	92.55
UACC-257	96.19
UACC-62	83.24
Ovarian Cancer	
IGROV1	106.00
OVCAR-3	101.25
OVCAR-4	95.37
OVCAR-5	113.52
OVCAR-8	96.20
NCI/ADR-RES	98.95
SK-OV-3	119.33
Renal Cancer	
786-0	87.46
A498	68.68
ACHN	103.72
CAKI-1	91.15
RXF 393	92.40
SN12C	97.47
TK-10	106.78
UO-31	79.51
Prostate Cancer	
PC-3	83.61
DU-145	100.88
Breast Cancer	
MCF7	90.28
MDA-MB-231/ATCC	89.90
HS 578T	108.46
BT-549	94.03
T-47D	95.24
MDA-MB-468	85.14
Mean	95.93
Delta	27.25
Range	55.96

Mean Growth Percent - Growth Percent

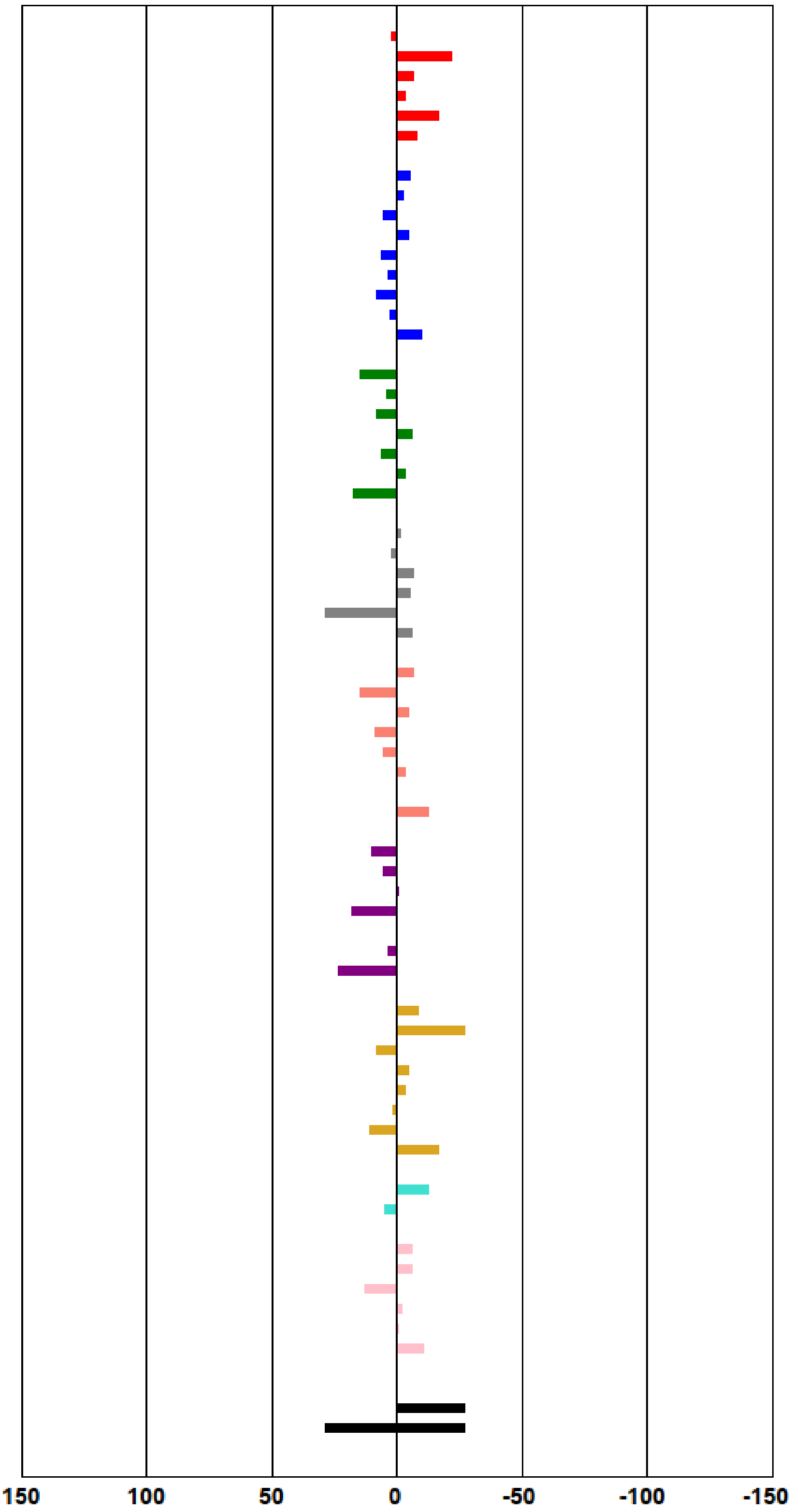

Anticancer Screening Data of 60 Cancer Cell Lines of **5f**

Developmental Therapeutics Program One Dose Mean Graph	NSC: D-827906 / 1	Conc: 1.00E-5 Molar	Test Date: Jan 25, 2021
	Experiment ID: **5f**		Report Date: Feb 12, 2021

Panel/Cell Line	Growth Percent
**Leukemia**	
CCRF-CEM	63.47
HL-60(TB)	45.58
K-562	28.03
MOLT-4	70.63
RPMI-8226	53.71
SR	16.95
**Non-Small Cell Lung Cancer**	
A549/ATCC	69.81
EKVX	83.73
HOP-62	80.75
HOP-92	68.36
NCI-H226	78.27
NCI-H23	83.96
NCI-H322M	101.71
NCI-H460	84.30
NCI-H522	57.23
**Colon Cancer**	
COLO 205	83.14
HCC-2998	97.78
HCT-116	73.69
HCT-15	50.80
HT29	70.26
KM12	57.81
SW-620	69.74
**CNS Cancer**	
SF-268	89.22
SF-295	58.51
SF-539	78.08
SNB-19	79.85
SNB-75	70.74
U251	74.21
**Melanoma**	
LOX IMVI	68.85
MALME-3M	72.18
M14	72.63
MDA-MB-435	2.79
SK-MEL-2	98.70
SK-MEL-28	84.27
SK-MEL-5	65.53
UACC-257	82.97
UACC-62	54.61
**Ovarian Cancer**	
IGROV1	68.95
OVCAR-3	54.59
OVCAR-4	79.90
OVCAR-5	104.92
OVCAR-8	83.51
NCI/ADR-RES	53.31
SK-OV-3	99.80
**Renal Cancer**	
786-0	98.53
A498	55.08
ACHN	74.70
CAKI-1	49.55
RXF 393	79.29
SN12C	97.49
TK-10	122.21
UO-31	68.30
**Prostate Cancer**	
PC-3	62.44
DU-145	92.17
**Breast Cancer**	
MCF7	42.50
MDA-MB-231/ATCC	82.50
HS 578T	82.40
BT-549	79.85
T-47D	68.88
MDA-MB-468	29.57
Mean	71.22
Delta	68.43
Range	119.42

Mean Growth Percent - Growth Percent

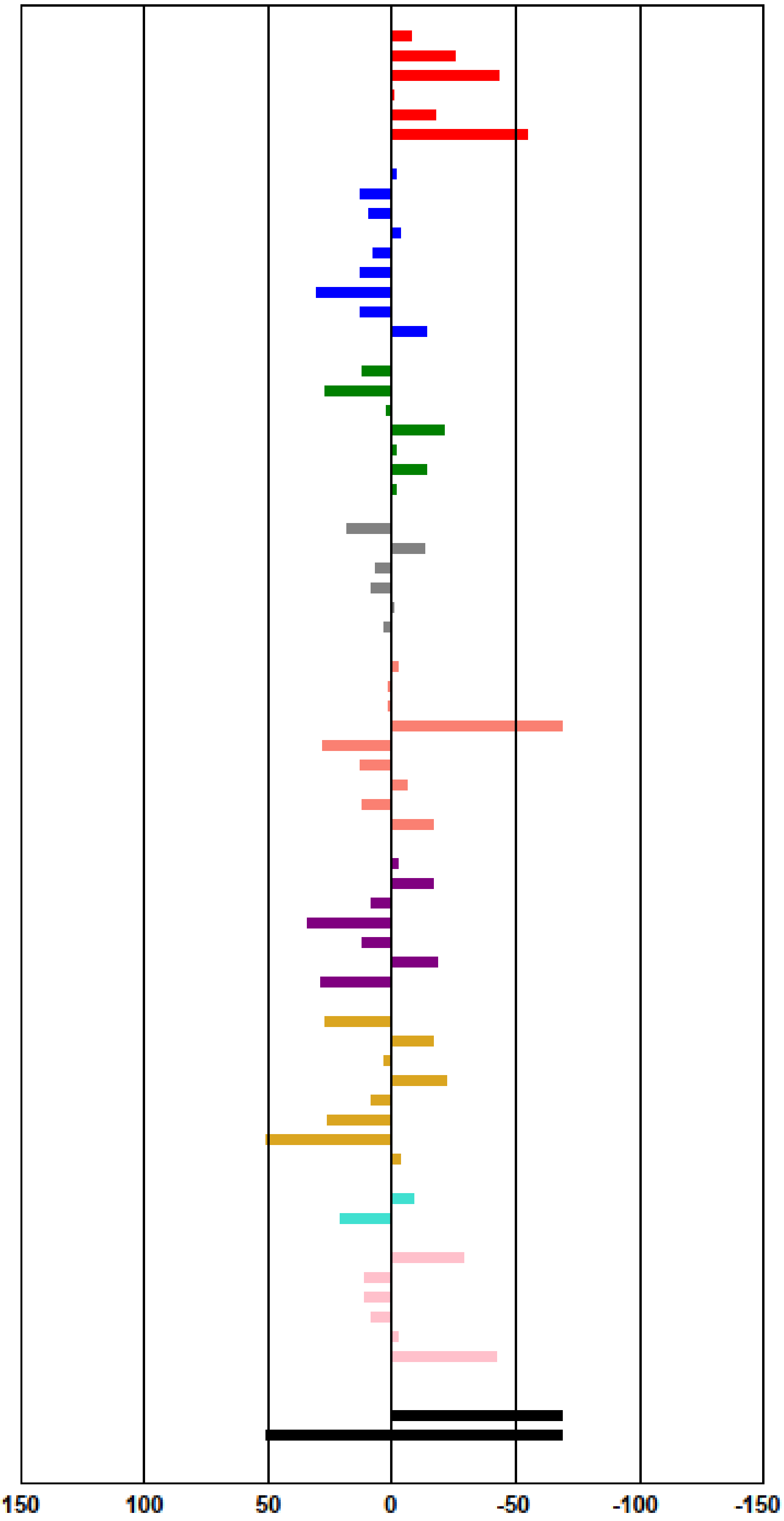

Supplement: Supplementary file 1 [file ijms-24-05756-s001.zip › ijms-2286177-supplementary.pdf]
